# A H_2_O_2_/HBr system – several directions but one choice: oxidation–bromination of secondary alcohols into mono- or dibromo ketones†

**DOI:** 10.1039/c8ra04885a

**Published:** 2018-08-10

**Authors:** Gennady I. Nikishin, Nadezhda I. Kapustina, Liubov L. Sokova, Oleg V. Bityukov, Alexander O. Terent'ev

**Affiliations:** N. D. Zelinsky Institute of Organic Chemistry of the Russian Academy of Sciences 47 Leninsky prosp. Moscow 119991 Russian Federation terentev@ioc.ac.ru

## Abstract

In this work we found that a H_2_O_2_–HBr(aq) system allows synthesis of α-monobromo ketones and α,α′-dibromo ketones from aliphatic and secondary benzylic alcohols with yields up to 91%. It is possible to selectively direct the process toward the formation of mono- or dibromo ketones by varying the amount of hydrogen peroxide and hydrobromic acid. The convenience of application, simple equipment, multifaceted reactivity, and compliance with green chemistry principles make the application of the H_2_O_2_–HBr(aq) system very attractive in laboratories and industry. The proposed oxidation–bromination process is selective in spite of known properties of ketones to be oxidized by the Baeyer–Villiger reaction or peroxidated with the formation of compounds with the O–O moiety in the presence of hydrogen peroxide and Bronsted acids.

## Abstract 2

Convenience of application, multifaceted reactivity, and compliance with green chemistry principles: H_2_O_2_–HBr(aq) system for preparation of bromo ketones with yields up to 91%.

## Graphical abstract

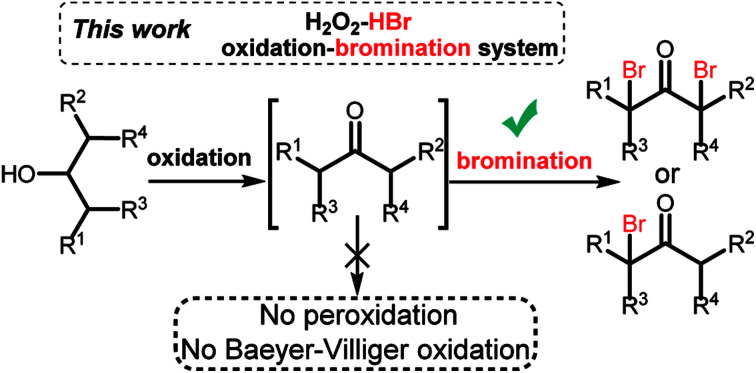

## Introduction

A wide variety of methods for the oxidation of secondary alcohols into ketones is regularly replenished with new oxidants and oxidizing systems. Hydrogen peroxide attracts increased attention as a cheap and environment-friendly oxidant. For the oxidation of secondary alcohols with H_2_O_2_, catalysts of different chemical nature were used: Br_2_,^1^ HBr,^1–6^ NaBr,^7,8^ BiBr_3_,^9^ FeBr_3_,^10^ MgBr_2_/[bmim] BF_4_,^11^ Mn(iii) complex,^12^ Co(ii) complex,^13^ Zn polyoxometalate,^14^ Na_2_WO_4_/PTC,^15–18^ [bmim]_4_[W_10_O_23_] in ionic liquids,^19^ W/phosphate,^20^ Keggin complexes,^21,22^ Al/W-polyoxoanions,^23^ W/Sc/P-salt.^24^

The H_2_O_2_–HBr(aq) system is known in organic chemistry for its application in the following processes: oxidation of secondary and primary alcohols to ketones^1–4^ and esters,^1,25^ respectively, bromination of olefins,^26,27^ acetylenes,^27^ ketones,^4,28^ arenes^26,27,29,30^ and heterocyclic compounds.^31^ This oxidation–bromination system is characterized by the use of inexpensive reagents, low environmental impact and the absence of organic wastes, which makes it a good alternative to existing oxidation and bromination methods.^32^ The H_2_O_2_–HBr(aq) system is also of interest for more extensive practical application.^33^ In our previous studies, this system was applied for the homo- and cross-condensation of alcohols into esters,^34^ the bromination of alkyl phenyl ketones into the aromatic ring and side-chain position.^35^

**Scheme 1:**
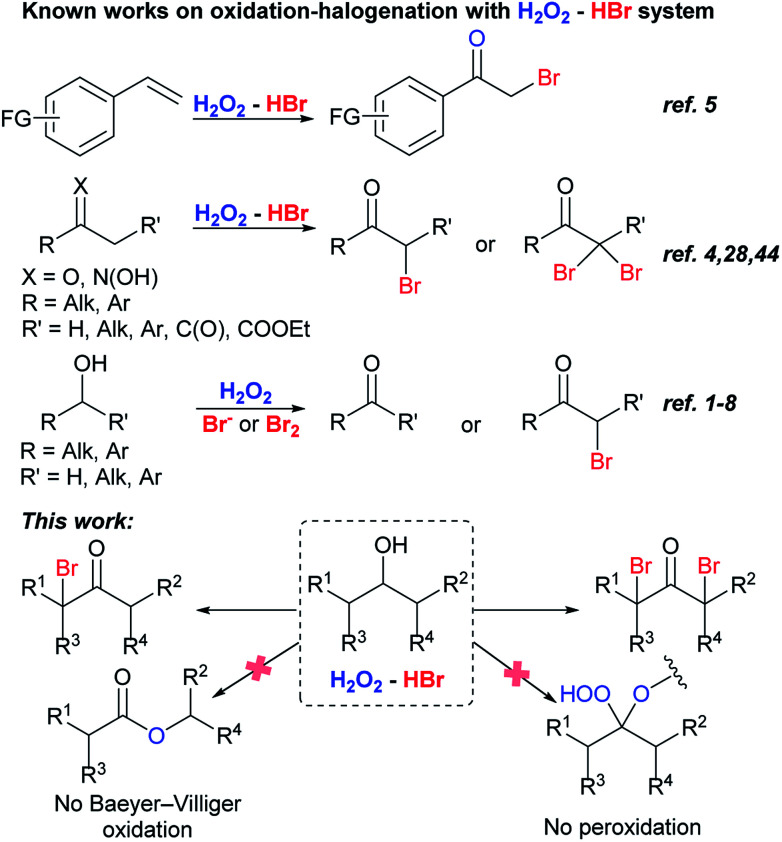
Oxidation-halogenation with H_2_O_2_–HBr(aq) system.

Bromo ketones are involved in organic chemistry as multipurpose reagents.^36,37^ They can be easily transformed into unsaturated ketones and ketones containing functional groups.^38,39^ Favorskii rearrangement of α-bromo ketones leads to the formation of esters.^40^ α,α′-Dibromo ketones are the precursors of 1,2-dialkyl cyclopropenones,^41,42^ and divinyl ketones,^43^ the latter ones are starting reagents for the synthesis of cyclopentenones by Nazarov reaction.^40^ H_2_O_2_–HBr(aq) system was used for the oxidative bromination of styrenes,^5^ ketones^4,28^ and oximes^44^ into bromo ketones. It is especially noteworthy that bromo ketones were not detected in the reactions with various substrates including alcohols under the action bromine containing systems: H_2_O_2_/HBr or hydrobromic acid sodium salt^1–8^ and H_2_O_2_/Br_2_.^1^ There is only one report of the oxidation of secondary alcohols into α-bromo ketones, but α,α′-dibromo ketones were not obtained (Scheme 1).^4^

This paper describes dibromo- and monobromo ketones synthesis by one-pot oxidation–bromination of secondary alcohols with H_2_O_2_–HBr(aq) (Scheme 1). The process is more experimentally simple, low-cost, and complies with green chemistry standards unlike the sole method proposed for the similar transformation, which is based on the oxidation–bromination of secondary alcohols with Ce(iv)/LiBr system.^45^ Change of hydrogen peroxide and hydrobromic acid amount can selectively direct the reaction routes towards the formation of mono- or dibromo ketones.

It is important to note that in the reaction conditions we didn't detect the formation of traditional Baeyer–Villiger oxidation products^46–48^ or expected peroxides,^49–61^ which are products of a reaction between C-atom of carbonyl group and hydrogen peroxide. Possible explanation of these reactions failure can be based on the higher rate of ketone bromination in competition with peroxidation and (or) following transformations. Steric hindrance in mono- and dibrominated ketones prevents the attack of hydrogen peroxide on the electrophilic carbon atom of the carbonyl group. To the best of our knowledge the only one example of α-brominated ketone peroxidation exists: 3-bromo-2,2-dihydroperoxy-1,7,7-trimethyl-bicyclo[2.2.1]heptane was obtained with H_2_O_2_–AlCl_3_ × 6H_2_O system.^62^

## Results and discussion

**Scheme 2:**
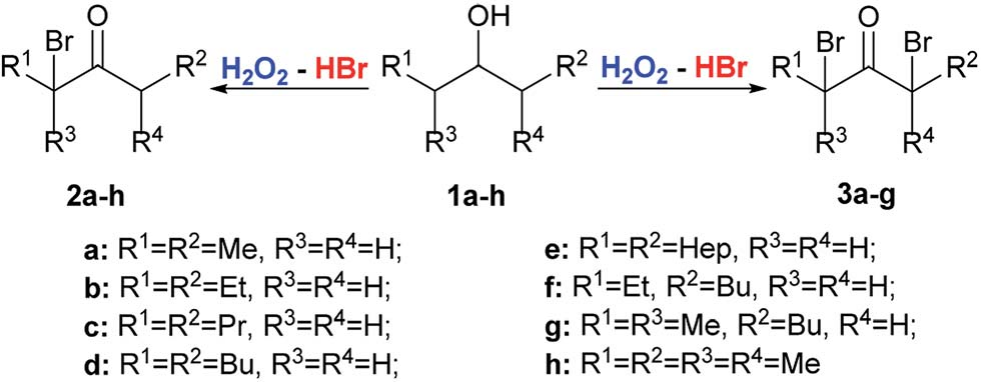
Oxidation–bromination of secondary alcohols 1a–h with the formation of α-bromoketones 2a–h or α,α′-dibromo ketones 3a–g.

**Scheme 3:**
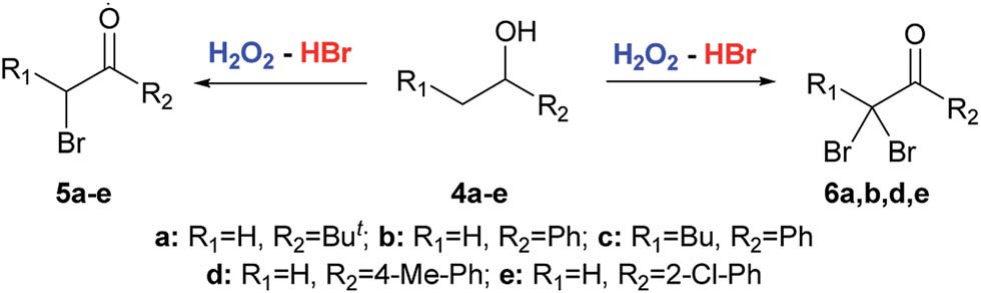
Oxidation–bromination of secondary alcohols 4a–e bearing only one α-CH_2_ group near the carbonyl group with the formation of α-bromoketones 5a–e or α,α-dibromo ketones 6a,b,d,e.

The starting substrates for the oxidation–bromination were symmetrical 1a–e, 1h and unsymmetrical aliphatic alcohols 1f-g and benzylic alcohols 4a–e (Schemes 2 and 3).

Depending on the experimental conditions and the structure of the alcohol 1a–h, it is possible to change the direction of the reaction, towards the formation of either α-bromo ketones 2a–h (molar ratio of 1a–h : HBr : H_2_O_2_ = 1 : 1.2 : 10) or α,α′-dibromo ketones 3a–g (molar ratio of 1a–h : HBr : H_2_O_2_ = 1 : 6 : 15) (Scheme 2).

α-Bromo ketones 5a–e (molar ratio of 4a–e : HBr : H_2_O_2_ = 1 : 1.2 : 10) and α,α-dibromo ketones 6a,b,d,e (molar ratio of 4a–e : HBr : H_2_O_2_ = 1 : 6:15) can be selectively prepared in the oxidation–bromination reactions with starting alcohols 4a–e bearing only one α-CH_2_ group near the carbonyl group (Scheme 3).

The influence of the experimental conditions on the selectivity of oxidation–bromination and on the yield of α-bromo ketone 2a, α,α′-dibromo ketone 3a, and ketone 7a was determined using pentanol-3 1a as an example. Generally, the reaction was carried out by the addition of 35% aqueous solution of H_2_O_2_ in acetonitrile for 6–10 hours to the solution of pentanol-3 1a and 48% HBr(aq) in acetonitrile or water (Table 1).

**Table 1:** Oxidation and oxidation–bromination of 1a with the H_2_O_2_–HBr(aq) system^a^

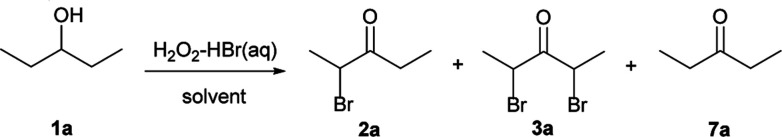							
Entry	Molar ratio: mole HBr and H_2_O_2_/mole 1a		Solvent	*τ*, *h*	Yield, %		
	HBr	H_2_O_2_			2a	3a	7a
1	1.2	5	CH_3_CN	6	38	Trace	55
2	1.2	10	CH_3_CN	6	80	Trace	Trace
3	1.4	10	CH_3_CN	6	76	13	—
4	2	10	CH_3_CN	6	60	35	Trace
5	2.5	10	CH_3_CN (50% aq.)	6	14	44	33
6	2.5	10	H_2_O	6	38	43	Trace
7	2.5	15	CH_3_CN	6	Trace	80	—
8	6	15	CH_3_CN	10	Trace	90	—
9	1.2	10	MeOH	6	40	—	52
10	1.2	10	THF	6	31	—	47
11	1.2	10	DCE	6	71	—	20

a General procedure: to a solution of alcohol 1a (1 mmol, 88.2 mg) and HBr (48% aq., 1.2–6 mmol, 0.136–0.679 ml) in 1 ml of a solvent at 65–70 °C (for entry 9 and 10, 60 °C) and vigorous stirring a solution of H_2_O_2_ (35% aq., 10–15 mmol, 0.860–1.290 ml) was added portionwise (0.2–0.3 ml) during 0.6–10 h. Yields were determined by GC analysis.

According to the obtained data, the highest yield of mono bromo ketone 2a was achieved with a 10-fold molar excess of H_2_O_2_ and 1.2 equivalents of HBr (entry 2). 5-Fold molar excess of H_2_O_2_ is insufficient for selective preparation of 2a. An increase of the HBr amount from 1.2 to 2.5 equivalents (entries 3–5) resulted in a decrease in the selectivity of products 2a, 3a, and 7a formation. When water was used as the solvent (entry 5), a mixture of monobromo ketones 2a and dibromo ketones 3a was also formed. The best yield of dibromo ketone 3a was achieved with a 15-fold molar excess of H_2_O_2_ (entries 7, 8). An increase of the HBr amount up to 6 equivalents and the reaction time up to 10 hours (entry 8) afforded the product 3a with the maximum yield (90%). In the best conditions for 2a preparation (entry 2), using of MeOH, THF, and DCE as a reaction media leads to the synthesis of 2a and 7a mixtures (entries 9–11).

Results of the optimization (Table 1) demonstrate that the reaction direction is mainly influenced by the molar ratio of the secondary alcohol, hydrogen peroxide, and hydrobromic acid. In the optimized reaction conditions (entry 2), a number of monobromo ketones 2a–h and 5a–e were prepared (Table 2). Optimized conditions (entry 7, Table 1) were also used for the synthesis of dibromo ketones 3a–g and 6a,b,d,e (Table 3).

**Table 2:** Oxidation–bromination of secondary alcohols 1a–h and 4a–e by the H_2_O_2_–HBr(aq) system to monobromo ketones 2a–h and 5a–e^a^

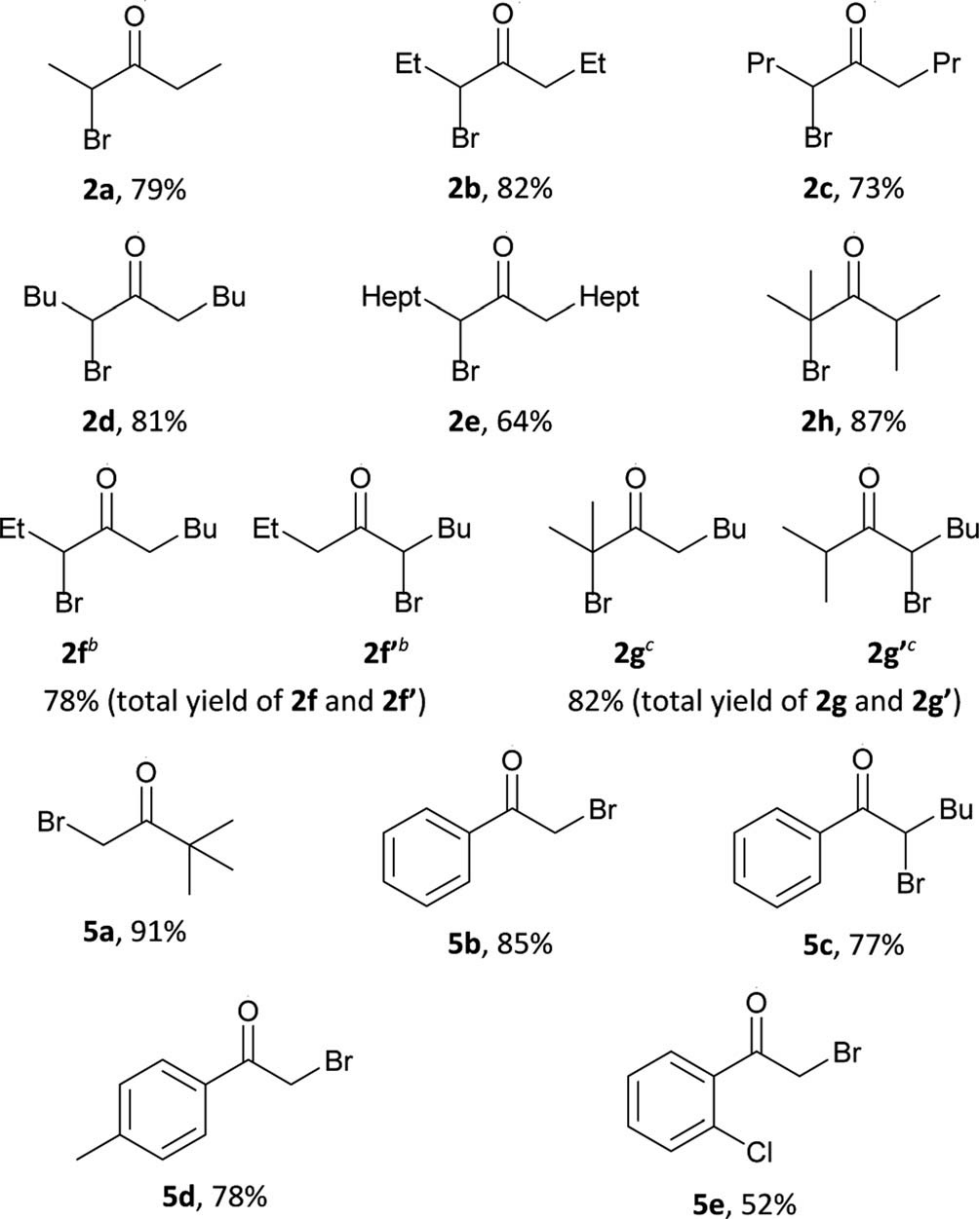

a General procedure: to a solution of alcohol 1a–h, 4a–e (1 mmol, 88.2–256.5 mg) and HBr (48% aq., 1.2 mmol, 0.136 ml) in CH_3_CN (1 ml) at 65–70 °C and vigorous stirring, a solution of H_2_O_2_ (35% aq.,10 mmol, 0.860 ml) in CH_3_CN (1 ml) was added portionwise (0.2–0.3 ml) for 6 hours.

b The ratio of isomers 2f : 2f′ ∼ 1 : 1 according to NMR data.

c The ratio of isomers 2g : 2g′ ∼ 2 : 1 according to NMR data.

**Table 3:** Oxidation–bromination of secondary alcohols 1a–h and 4a–e by the H_2_O_2_–HBr(aq) system to dibromo ketones 3a–g and 6a,b,d,e^a^

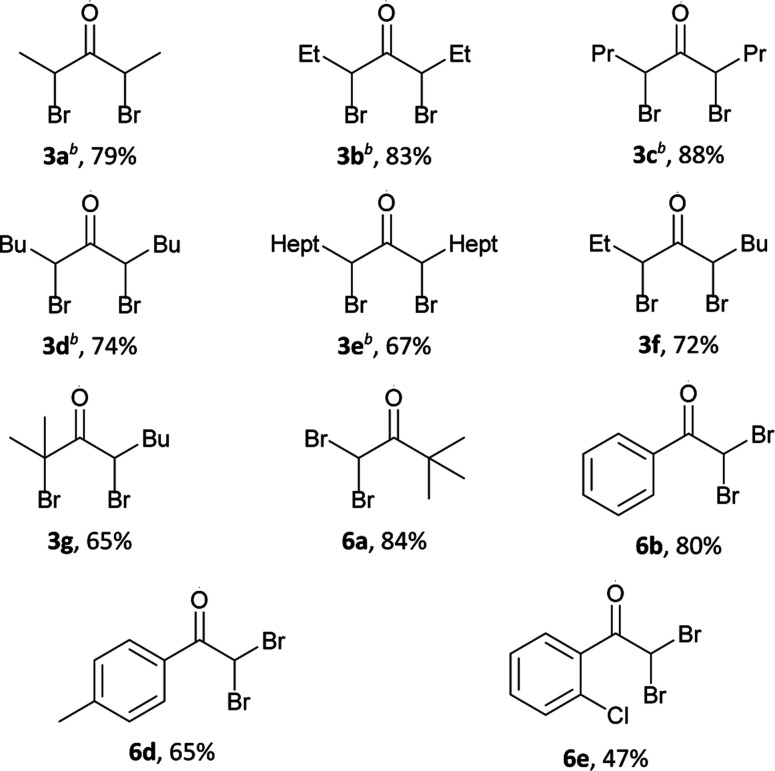

a General procedure: to a solution of alcohol 1a–h, 4a–c (1 mmol, 88.2–256.5 mg) and HBr (48% aq., 6 mmol, 0.679 ml) in CH_3_CN (1 ml) at 65–70 °C and vigorous stirring, a solution of H_2_O_2_ (35% aq., 15 mmol, 1.290 ml) in CH_3_CN (1 ml) was added portionwise (0.2–0.3 ml) for 10 hours.

b The ratio of diastereoisomers meso : rac = 1 : 3 according to NMR data.

Alcohols of unbranched structure 1a–f are easily converted into α-bromo ketones (Table 2) and into α,α′-dibromo ketones (Table 3) with good yields. Diisopropyl carbinol 1h is converted only to the corresponding α-bromo ketone 2h (Table 2), the formation of α,α′-dibromo ketone from diisopropyl carbinol 1h is probably prevented by the steric hindrance. In the case of isopropyl amyl carbinol 1g, two isomeric mono bromides, 2-bromo-2-methyloctan-3-one 2g and 4-bromo-2-methyloctan-3-one 2g′ are formed in a ratio of 1 : 1 (Table 2). Oxidation–bromination of propyl amyl carbinol 1f also results in two monobromo ketones, 3-bromononan-4-one 2f and 5-bromononan-4-one 2f′ (∼1 : 1) (Table 2). Methyl *tert*-butyl carbinol 4a and methyl phenyl carbinol 4b are converted to α,α-dibromo ketone 6a and α,α-dibromo ketone 6b (Table 3) under optimized conditions for dibromo ketone synthesis (entry 7, Table 1). Amyl phenyl carbinol 4c undergoes only monobromination with the formation of 5c (Table 2). In the reaction medium symmetric α,α′-dibromo ketones are formed as a mixture of meso- and rac-isomers with a rac-form preference (ratio 1 : 3), as evidenced by ^1^H and ^13^C NMR spectra.

## Conclusions

Thus, we have developed a convenient one-pot method for the synthesis of monobromo- and dibromo ketones by oxidation–bromination of secondary alcohols with the H_2_O_2_/HBr system in acetonitrile. Formation of peroxides and products of Baeyer–Villiger reaction was not detected. Amount and molar ratio of hydrogen peroxide and hydrobromic acid are the key factors for the achievement of a high degree of selectivity. Optimized conditions permits to synthesize α,α′-dibromo- or α,α-dibromo ketones with yields up to 91%. Conversion of unbranched secondary alcohols into α,α′-dibromo ketones is the most effective.

## Experimental

### Experimental for the Table 1

To a solution of alcohol 1a (1 mmol, 88.2 mg) and HBr (48% aqueous) (1.2–6 mmol, 0.136–0.679 ml) in 1 ml of solvent (CH_3_CN, CH_3_CN (50% aq.), H_2_O, DCE) at 65–70 °C (for MeOH (entry 9) and THF (entry 10), 60 °C) and vigorous stirring, a solution of H_2_O_2_ (35% aqueous, 10–15 mmol, 0.860–1.290 ml) was added portionwise (0.2–0.3 ml) during 6–10 h. After the addition of the first portion, brown vapours and a bright orange colour were observed. The next portions of H_2_O_2_ were added after the decolorization of the reaction mixture (a pale-yellow colour), then the reaction mass was cooled, diethyl ether (15 mL) and Na_2_SO_3_ (1 g) were added. The organic layer was decanted and washed with water (5 ml), then dried over Na_2_SO_4_. The solvent was evaporated in a vacuum of a water jet pump (20 mmHg). Yields of 2a, 3a, 7a were determined by GC analysis with heptan-4-one and undecan-6-one as the internal standard.

### Experimental for the Table 2

To a solution of alcohol 1a–h, 4a–e (1 mmol, 88.2–256.5 mg) and HBr (48% aqueous, 1.2 mmol, 0.136 ml) in CH_3_CN (1 ml) at 65–70 °C and vigorous stirring, a solution of H_2_O_2_ (35% aqueous, 10 mmol, 0.860 ml) in CH_3_CN (1 ml) was added portionwise (0.2–0.3 ml) for 6 hours. After the addition of the first portion, brown vapors and a bright orange color were observed. The next portions of H_2_O_2_ were added after the decolorization of the reaction mixture (a pale-yellow color), then the reaction mass was cooled, diethyl ether (15 mL) and Na_2_SO_3_ (1 g) were added. The organic layer was decanted and washed with water (5 ml), then dried over Na_2_SO_4_. The solvent was evaporated in a vacuum of a water jet pump (20 mmHg). The products 2a–h and 5a–e were isolated by column chromatography on silica gel in a solvent system PE : EA (100 : 1).

### Experimental for the Table 3

To a solution of alcohol 1a–h, 4a–e (1 mmol, 88.2–256.5 mg) and HBr (48% aqueous, 6 mmol, 0.679 ml) in CH_3_CN (1 ml) at 65–70 °C and vigorous stirring, a solution of H_2_O_2_ (35% aqueous, 15 mmol, 1.290 ml) in CH_3_CN (1 ml) was added portionwise (0.2–0.3 ml) for 6 hours. After the addition of the first portion, brown vapors and a bright orange color were observed. The next portion of hydrogen peroxide was added after the decolorization of the reaction mixture (a pale-yellow color), then the reaction mass was cooled, diethyl ether (15 mL) and Na_2_SO_3_ (1 g) were added. The organic layer was decanted and washed with water (5 ml) and then dried over Na_2_SO_4_. The solvent was evaporated in a vacuum of a water jet pump (20 mmHg). Products 3a–g and 6a,b,d,e were isolated by column chromatography on silica gel in a solvent system PE : EA (100 : 1).

## Conflicts of interest

There are no conflicts to declare.

## Supplementary Material

RA-008-C8RA04885A-s001
