# Supplementary material for: A H2O2/HBr system – several directions but one choice: oxidation–bromination of secondary alcohols into mono- or dibromo ketones
Source: RSC Adv. 2018 Aug 10;8(50):28632–6. doi: 10.1039/c8ra04885a (PMC9084404; doi:10.1039/c8ra04885a)

Electronic Supplementary Material (ESI) for RSC Advances.
This journal is © The Royal Society of Chemistry 2018

Supporting Information

H_2O_2/HBr system - several directions but one choice: oxidation-bromination of secondary alcohols into mono- or dibromo ketones

Gennady I. Nikishin, Nadezhda I. Kapustina, Liubov L. Sokova, Oleg V. Bityukov, Alexander O. Terent'ev*

N. D. Zelinsky Institute of Organic Chemistry, Russian Academy of Sciences, 47, Leninsky prosp., 119991 Moscow, Russian Federation, Fax: +7 499 1355328

*e-mail: terentev@ioc.ac.ru

Table of contents

General materials and methods S3

Experimental for Table 1 S3

Experimental for Table 2 S3

Experimental for Table 3 S4

Characterization of the products S5

References S13

Copies of ^{1}H, ^{13}C NMR and HRMS spectra of synthesized products S14

General materials and methods

^1H and ^{13}C NMR spectra were recorded on Bruker AVANCE II 300 spectrometer (300.13 and 75.48 MHz, respectively) in $CDCl_3$. Chemical shifts were reported in parts per million (ppm), and the residual solvent peak was used as an internal reference: ^1H ($CDCl_3$ δ=7.26 ppm), ^{13}C ($CDCl_3$ δ=77.16 ppm). Multiplicity was indicated as follows: s (singlet), d (doublet), t (triplet), q (quartet), m (multiplet).

High resolution mass spectra (HR-MS) were measured on a Bruker micrOTOF II instrument using electrospray ionization (ESI). The measurements were performed in a positive ion mode (interface capillary voltage – 4500 V); mass range from m/z 50 to m/z 3000 Da; external calibration with Electrospray Calibrant Solution (Fluka). A syringe injection was used for all acetonitrile solutions (flow rate 3 μL/min). Nitrogen was applied as a dry gas; interface temperature was set at 180 °C.

GC analysis was performed on a Chrom-5 chromatograph with the flame-ionization detector and 3x3000 mm analytical glass columns with 5% SE-30 and 5% FFAP on Chromaton N-AW-HMDS (0.16-0.20 mm). The product yields were determined by an internal standard method with the empirical correlation coefficients.

The TLC analysis was carried out on standard silica gel chromatography plates (DC-Fertigfolien ALUGRAM^R Xtra SIL G/UV_{254}). Column chromatography was performed using silica gel (0.060-0.200 mm, 60 A, CAS 7631-86-9, Acros).

Petroleum ether (PE, 40/70), MeOH, THF, dichloroethane (DCE) were distilled prior to use. MeCN were distilled over P_2O_5. EtOAc were purchased from commercial sources and were used as is.

Pentan-3-ol (**1a**), 2,4-dimethylpentan-3-ol (**1h**), nonan-4-ol (**1f**), 2-methyloctan-3-ol (**1g**), 1-phenylhexan-1-ol (**4c**), HBr (48% solution in water), H_2O_2 (35% aqueous solution), Na_2SO_4, Na_2SO_3 were commercial reagents (Acros, Sigma-Aldrich). Other secondary alcohols were synthesized according to the literature[1].

Experimental for Table 1

To a solution of alcohol **1a** (1 mmol, 88.2 mg) and HBr (48% aqueous, 1.2-6 mmol, 0.136-0.679 ml) in 1 ml of solvent at 65-70 °C and vigorous stirring, a solution of H_2O_2 (35% aqueous, 10-15

mmol, 0.860-1.290 ml) was added portionwise (0.2-0.3 ml) during 6-10 h. After the addition of the first portion, brown vapors and a bright orange color of the reaction mass were observed. The next portions of H_2O_2 were added after the decolorization of the reaction mixture (a pale-yellow color), then the reaction mass was cooled, diethyl ether (15 mL) and Na_2SO_3 (1 g) were added. The organic layer was decanted and washed with water (5 ml), then dried over Na_2SO_4. The solvent was evaporated in a vacuum of a water jet pump (20 mmHg). Yields **2a**, **3a** and **7a** were determined by GLC using heptan-4-one and undecane-6-one as the internal standards.

Experimental for the Table 2

To a solution of alcohol **1a-h**, **4a-e** (1 mmol, 88.2-256.5 mg) and HBr (48% aqueous, 1.2 mmol, 0.136 ml) in CH_3CN (1 ml) at 65-70 °C and vigorous stirring, a solution of H_2O_2 (35% aqueous, 10 mmol, 0.860 ml) in CH_3CN (1 ml) was added portionwise (0.2-0.3 ml) for 6 hours. After the addition of the first portion, brown vapors and a bright orange color of the reaction mass were observed. The next portions of H_2O_2 were added after the decolorization of the reaction mixture (a pale-yellow color), then the reaction mass was cooled, diethyl ether (15 mL) and Na_2SO_3 (1 g) were added. The organic layer was decanted and washed with water (5 ml), then dried over Na_2SO_4. The solvent was evaporated in a vacuum of a water jet pump (20 mmHg). The products **2a-h** and **5a-e** were isolated by column chromatography on silica gel in a solvent system PE: EA (100:1).

Experimental for the Table 3

To a solution of alcohol **1a-h**, **4a-e** (1 mmol, 88.2-256.5 mg) and HBr (48% aqueous, 6 mmol, 0.679 ml) in CH_3CN (1 ml) at 65-70 °C and vigorous stirring, a solution of H_2O_2 (35% aqueous, 15 mmol, 1.290 ml) in CH_3CN (1 ml) was added portionwise (0.2-0.3 ml) for 6 hours. After the addition of the first portion, brown vapors and a bright orange color of the reaction mass were observed. The next portion of hydrogen peroxide was added after the decolorization of the reaction mixture (a pale-yellow color), then the reaction mass was cooled, diethyl ether (15 mL) and Na_2SO_3 (1 g) were added. The organic layer was decanted and washed with water (5 ml) and then dried over Na_2SO_4. The solvent was evaporated in a vacuum of a water jet pump (20 mmHg). The products **3a-g** and **6a,b,d,e** were isolated by column chromatography on silica gel in a solvent system PE: EA (100:1).

Characterization of the products

All the new compounds were characterized using ^{1}H and ^{13}C NMR spectroscopy, HR-MS spectroscopy. ^{1}H and ^{13}C NMR spectra of the known compounds were in agreement with the literature data[2-11].

2-Bromopentan-3-one, 2a[9]

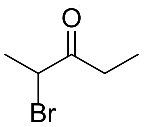

Colorless oil (130 mg, 79%)

^{1}H NMR (300.13 MHz, $CDCl_3$) δ: 1.10 (t, 3H, CH_3, *J* = 7.2 Hz), 1.72 (d, 3H, $\underline{CH_3}$CHBr, *J* = 6.9 Hz), 2.51-2.65 (m, 1H, CH_2), 2.78-2.92 (m, 1H, CH_2), 4.40 (q, CHBr, *J* = 6.9 Hz);

^{13}C NMR (75.47 MHz, $CDCl_3$) δ: 8.17 (CH_3), 20.14 ($\underline{CH_3}$CHBr), 31.95 (CH_2), 47.26 (CHBr), 205.09 (CO).

3-Bromoheptan-4-one, 2b[10]

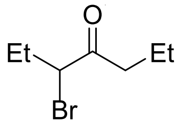

Colorless oil (158 mg, 82%)

^{1}H NMR (300.13 MHz, $CDCl_3$) δ: 0.93 (t, 3H, CH_3, *J* = 7.3 Hz), 1.00 (t, 3H, CH_3, *J* = 7.3 Hz), 1.58-1.70 (m, 2H, CH_2), 1.90-2.07 (m, 2H, $\underline{CH_2}$CHBr), 2.57-2.71 (m, 2H, CH_2CO), 4.16 (dd, 1H, CHBr, *J* = 6.4 Hz, *J* = 8.0 Hz);

^{13}C NMR (75.47 MHz, $CDCl_3$) δ: 11.97 (CH_3), 13.58 (CH_3), 17.39 (CH_2), 26.88 ($\underline{C}H_2$CHBr), 40.90 ($\underline{C}H_2$CO), 55.48 (CHBr), 204.21 (CO).

4-Bromononan-5-one, 2c[11]

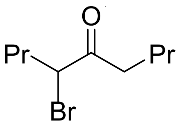

Colorless oil (161 mg, 73%)

^{1}H NMR (300.13 MHz, $CDCl_3$) δ: 0.88-0.95 (m, 6H, 2CH_3), 1.28-1.61 (m, 6H, 3CH_2), 1.84-2.00 (m, 2H, $\underline{CH_2}$CHBr), 2.56-2.75 (m, 2H, CH_2CO), 4.23 (dd, 1H, CHBr, J = 6.5 Hz, J = 8.0 Hz);
^{13}C NMR (75.47 MHz, $CDCl_3$) δ: 13.39 (CH_3), 13.78 (CH_3), 20.60 (CH_2), 22.18 (CH_2), 26.04 (CH_2), 35.39 ($\underline{C}H_2$CHBr), 38.64 ($\underline{C}H_2$CO), 53.53 (CHBr), 204.37 (CO).

5-Bromoundecan-6-one, 2d[7, 10]

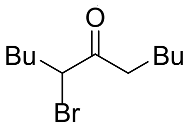

Colorless oil (201 mg, 81%)
^{1}H NMR (300.13 MHz, $CDCl_3$) δ: 0.86-0.92 (m, 6H, 2CH_3), 1.25-1.46 (m, 8H, 4CH_2), 1.55-1.65 (m, 2H, CH_2), 1.85-2.04 (m, 2H, $\underline{CH_2}$CHBr), 2.57-2.72 (m, 2H, CH_2CO), 4.22 (dd, 1H, CHBr, J = 6.7 Hz, J = 7.9 Hz);
^{13}C NMR (75.47 MHz, $CDCl_3$) δ: 13.80 (CH_3), 13.87 (CH_3), 22.10 (CH_2), 22.40 (CH_2), 23.64 (CH_2), 29.47 (CH_2), 31.23 (CH_2), 33.18 ($\underline{C}H_2$CHBr), 38.91 ($\underline{C}H_2$CO), 53.79 (CHBr), 204.40 (CO).

8-Bromoheptadecan-9-one, 2e

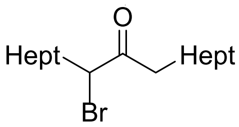

Colorless oil (213 mg, 64%)
^{1}H NMR (300.13 MHz, $CDCl_3$) δ: 0.87 (t, 6H, 2CH_3, J = 6.6 Hz), 1.27 (m, 20H, CH_2), 1.58-1.62 (m, 2H CH_2), 1.88-1.97 (m, 2H, C$\underline{H_2}$CHBr), 2.58-2.72 (m, 2H, CH_2CO), 4.22 (t, 1H, CHBr, J = 7.3 Hz);
^{13}C NMR (75.47 MHz, $CDCl_3$) δ: 14.03 (CH_3), 14.05 (CH_3), 22.58 (CH_2), 22.63 (CH_2), 23.99 (CH_2), 27.36 (CH_2), 28.95 (CH_2), 28.98 (CH_2), 29.08 (CH_2), 29.11 (CH_2), 29.31 (CH_2), 31.68 (CH_2), 31.81 (CH_2), 33.49 (CH_2), 38.95 ($\underline{C}H_2$CO), 53.84 (CHBr), 204.41 (CO);
HRMS (ESI) m/z $[M+Na]^+$: Calcd for $[C_{17}H_{33}BrNaO]^+$: 355.1607. Found: 355.1603.
HRMS-ESI: calculated 355.1607 (^{79}Br) and 357.1587 (^{81}Br) $[C_{17}H_{33}BrNaO]^+$ found 355.1603 (^{79}Br) and 357.1584 (^{81}Br)

2-Bromo-2,4-dimethylpentan-3-one, 2h[10]

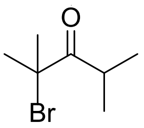

Colorless oil (168 mg, 87%)

^{1}H NMR (300.13 MHz, $CDCl_3$) δ: 1.16 (d, 6H, $2CH_3$, J = 6.6 Hz), 1.85 (s, 6H, $2CH_3$), 3.37-3.50 (m, 1H CH);

^{13}C NMR (75.47 MHz, $CDCl_3$) δ: 20.89, 29.30, 34.54, 64.56, 209.93.

Mixture of 3-bromononan-4-one (2f) and 5-bromononan-4-one (2f')[5] (ratio **2f** : **2f'** according to NMR spectroscopy data ~ 1 : 1)

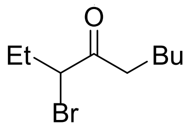

2f

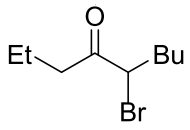

2f'

Colorless oil (172 mg, 78%)

^{1}H NMR (300.13 MHz, $CDCl_3$) δ: 0.86-0.95 (m, 9H, $3CH_3$), 1.00 (t, 3H, CH_3, J = 7.3 Hz), 1.23-1.39 (m, 8H, $4CH_2$), 1.58-1.70 (m, 4H, $2CH_2$), 1.87-2.08 (m, 4H, $2\underline{CH_2}CHBr$), 2.54-2.75 (m, 4H, $2CH_2CO$), 4.17 (dd, H, CHBr, J = 6.4 Hz), 4.21 (dd, H, CHBr, J = 6.3 Hz);

^{13}C NMR (75.47 MHz, $CDCl_3$) δ: 11.97 (CH_3), 13.58 (CH_3), 13.78 (CH_3), 13.87 (CH_3), 17.40 (CH_2), 22.10 (CH_2), 22.40 (CH_2), 23.63 (CH_2), 26.88 (CH_2), 29.46 (CH_2), 31.22 (CH_2), 33.15 (CH_2), 39.00 (CH_2CO), 40.80 (CH_2CO), 53.78 (CHBr), 55.48 (CHBr), 204.23 (CO), 204.34 (CO).

Mixture of 2-bromo-2-methyloctan-3-one (2g) and 4-bromo-2-methyloctan-3-one (2g') (ratio **2g** : **2g'** according to NMR spectroscopy data ~ 2 : 1)

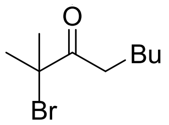

2g

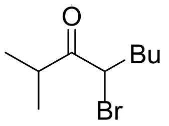

2g'

Colorless oil (181 mg, 82%)

^{1}H NMR (300.13 MHz, $CDCl_3$) δ: 0.89 (t, 4.5H, $2CH_3$, J = 6.8 Hz), 1.12 (d, 1.5H, CH_3, J = 6.9 Hz), 1.16 (d, 1.5H, CH_3, J = 6.7 Hz), 1.26-1.36 (m, 6H, $4CH_2$), 1.56-1.70 (m, 3H, $2CH_2$), 1.84 (s,

6H, $2CH_3$), 2.78 (t, 2H, CH_2CO, J = 7.3 Hz), 2.97-3.07 (m, 0.5H, CHCO), 4.36 (t, 0.5H, CHBr, J = 7.2 Hz);

^{13}C NMR (75.47 MHz, $CDCl_3$) δ: 13.81(CH_3), 13.90 (CH_3), 18.65 (CH_2), 19.36 (CH_2), 22.18 (CH_3), 22.45 (CH_2), 24.44 (CH_2), 29.57 (CH_3), 31.24 (CH_2), 32.98 (CH_2), 36.04 ($\underline{C}H_2CO$), 37.97 ($\underline{C}HCO$), 51.70 (CHBr), 64.03 (CBr), 205.75 (CO), 207.63 (CO).

HRMS-ESI: calculated 243.0355 (^{79}Br) and 245.0335 (^{81}Br) $[C_9H_{17}BrNaO]^+$ found 243.0348 (^{79}Br) and 245.0340 (^{81}Br)

1-Bromo-3,3-dimethylbutan-2-one, 5a[9]

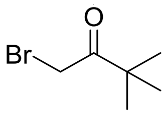

Colorless oil (163 mg, 91%)

^1H NMR (300.13 MHz, $CDCl_3$) δ: 1.21 (s, 9H, CH_3), 4.16 (s, 2H, CH_2);

^{13}C NMR (75.47 MHz, $CDCl_3$) δ: 26.70 ($3CH_3$), 31.60 (CH_2Br), 44.21 ($\underline{C}(CH_3)_3$), 206.03 (CO).

2-Bromo-1-phenylethanone, 5b[8]

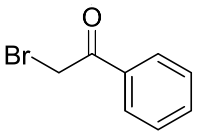

Pale yellow powder (169 mg, 85%); mp = 48-50 °C.

^1H NMR (300.13 MHz, $CDCl_3$) δ: 4.45 (s, 2H, CH_2Br), 7.48 (t, 2H, 2CH, J = 7.3 Hz), 7.60 (t, 1H, CH, J =7.3 Hz), 7.98 (d, 2H, 2CH, J = 7.3 Hz);

^{13}C NMR (75.47 MHz, $CDCl_3$) δ: 30.88 (CH_2Br), 128.83 (2CH), 128.91 (2CH), 133.93 (2CH), 191.25 (CO).

2-Bromo-1-phenylhexan-1-one, 5c[3]

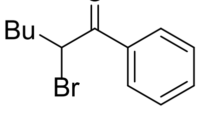

Pale yellow oil (196 mg, 77%)

^1H NMR (300.13 MHz, $CDCl_3$) δ: 0.92 (t, 3H, CH_3, J = 7.3 Hz), 1.33-1.53 (m, 4H, $2CH_2$), 2.07-2.27 (m, 2H, $\underline{CH_2}CHBr$), 5.13 (t, 1H, CHBr, J = 7.3 Hz), 7.48 (t,2H, 2CH, J = 7.3 Hz), 7.59 (t, 1H, CH, J = 7.3 Hz), 8.01 (d, 2H, 2CH, J = 7.3 Hz);

^{13}C NMR (75.47 MHz, $CDCl_3$) δ: 13.84 (CH_3), 22.25 (CH_2), 29.63 (CH_2), 33.22 (CH_2), 47.26 (CHBr), 128.73 (2CH), 128.80 (2CH), 133.61(CH), 134.52 (C), 193.27 (CO).

2-Bromo-1-(p-tolyl)ethan-1-one, 5d[12]

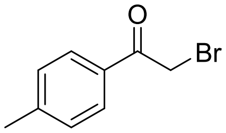

White solid (166 mg, 78%); mp 53-54°C

^{1}H NMR (300.13 MHz, $CDCl_3$) δ: 2.41 (s, 3H, CH_3), 4.41 (s, 2H; CH_2), 7.27 (d, *J* = 8.0 Hz, 2H), 7.86 (d, *J* = 8.0 Hz, 2H);

^{13}C NMR (75.47 MHz, $CDCl_3$) δ: 21.71 (CH_3), 30.90 (CH_2), 129.01 (CH), 129.50 (CH), 131.44 (C), 144.96 (C), 190.90 (CO).

2-Bromo-1-(2-chlorophenyl)ethan-1-one, 5e[13]

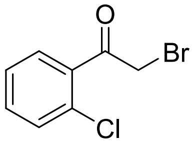

Pale yellow oil (121 mg, 52%)

^{1}H NMR (300.13 MHz, $CDCl_3$) δ: 4.51 (s, 2H; CH_2), 7.34-7.38 (m, 1H), 7.43-7.44 (m, 2H), 7.54-7.56 (m, 1H);

^{13}C NMR (75.47 MHz, $CDCl_3$) δ: 34.45 (CH_2), 127.13 (CH), 130.25 (CH), 130.57 (CH), 131.30 (CH), 132.73 (C), 136.24 (C), 194.01 (CO).

2,4-Dibromopentan-3-one (mixture of *meso*- and *rac*-isomers 1:2), 3a[4, 9]

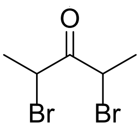

Colorless oil (192 mg, 79%)

^{1}H NMR (300.13 MHz, $CDCl_3$) δ: **pair dl (rac)**: 1.79 (d, 3H, CH_3, *J* = 6.6 Hz), 4.97 (q, 1H, CHBr, *J* = 6.6 Hz), **meso**: 1.86 (d, 3H, CH_3, *J* = 6.6 Hz), 4.76 (q, 1H, CHBr, *J* = 6.6 Hz);

^{13}C NMR (75.47 MHz, $CDCl_3$) δ: **pair dl (rac)**: 19.50 ($2CH_3$), 43.82 (CHBr), 195.99 (CO), **meso**: 21.74 ($2CH_3$), 44.01 (CHBr), 197.99 (CO).

3,5-Dibromoheptan-4-one, 3b[2, 9]

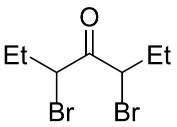

Colorless oil (225 mg, 83%)

^{1}H NMR (300.13 MHz, $CDCl_3$) δ: **pair dl (rac):** 1.03 (t, 6H, $2CH_3$, J = 7.3 Hz), 1.94-2.04 (m, 2H, CH_2), 2.12-2.21 (m, 2H, CH_2), 4.65 (t, 2H, CHBr, J = 7.3 Hz);

^{13}C NMR (75.47 MHz, $CDCl_3$) δ: **pair dl (rac):** 11.88 ($2CH_3$), 26.06 ($2CH_2$), 51.70 (2CHBr), 194.34(CO).

4,6-Dibromononane-5-one, 3c[6, 9]

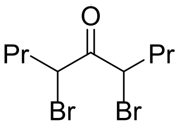

Colorless oil (264 mg, 88%)

^{1}H NMR (300.13 MHz, $CDCl_3$) δ: **pair dl (rac):** 0.97 (t, 6H, CH_3, J = 7.3 Hz), 1.38-1.52 (m, 4H, $2CH_2$), 1.90-2.01 (m, 2H, CH_2), 2.05-2.15 (m, 2H, CH_2), 4.73 (t, 2H, 2CHBr, J = 7.3 Hz);

^{13}C NMR (75.47 MHz, $CDCl_3$) δ: **pair dl (rac):** 13.46 ($2CH_3$), 20.52 ($2CH_2$), 34.56 ($2CH_2$), 49.87 (2CHBr), 194.38 (CO).

5,7-Dibromoundecan-6-one, 3d

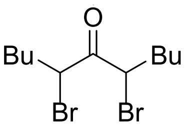

Pale yellow oil (242 mg, 74%)

^{1}H NMR (300.13 MHz, $CDCl_3$) δ: **pair dl (rac):** 0.91 (t, 6H, $2CH_3$, J = 6.6 Hz), 1.38-1.45 (m, 8H, $4CH_2$), 1.92-1.99 (m, 2H, CH_2), 2.08-2.20 (m, 2H, CH_2), 4.71 (t, 2H, 2CHBr, J = 7.3 Hz);

^{13}C NMR (75.47 MHz, $CDCl_3$) δ: **pair dl (rac):** 13.78 ($2CH_3$), 22.16 ($2CH_2$), 29.30 ($2CH_2$), 32.27 ($2CH_2$), 50.15 (2CHBr), 194.38 (CO);

HRMS-ESI: calculated 350.9753 (^{79}Br) and 352.9732 (^{81}Br) $[C_{11}H_{20}Br_2NaO]^+$ found 350.9749 (^{79}Br) and 352.9730 (^{81}Br)

8,10-Dibromoheptadecan-9-one, 3e

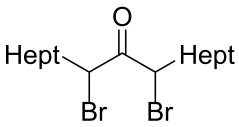

Pale yellow oil (276 mg, 67%)

^{1}H NMR (300.13 MHz, $CDCl_3$) δ: **pair dl (rac):** 0.87 (t, 6H, $2CH_3$, *J* = 6.6 Hz), 1.27-1.39 (m, 20H, $10CH_2$), 1.92-1.98 (m, 2H, CH_2), 2.07-2.14 (m, 2H, CH_2), 4.71 (t, 2H, 2CHBr, *J* = 7.3 Hz);

^{13}C NMR (75.47 MHz, $CDCl_3$) δ: **pair dl (rac):** 14.05 ($2CH_3$), 22.59 ($2CH_2$), 27.19 ($2CH_2$), 28.98 ($2CH_2$), 31.69 ($2CH_2$), 32.55 ($2CH_2$), 50.19 (2CHBr), 194.40 (CO);

HRMS-ESI: calculated 433.0712 (^{79}Br) and 435.0692 (^{81}Br) $[C_{17}H_{32}Br_2NaO]^+$ found 433.0716 (^{79}Br) and 435.0703 (^{81}Br)

3,5-Dibromononan-4-one, 3f

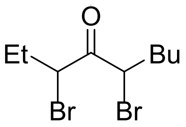

Pale yellow oil (216 mg, 72%)

^{1}H NMR (300.13 MHz, $CDCl_3$) δ: **pair dl (rac):** 0.91 (t, 3H, CH_3, *J* = 6.6 Hz), 1.03 (t, 3H, CH_3, *J* = 7.3 Hz), 1.36-1.39 (m, 4H, $2CH_2$), 1.92-2.04 (m, 2H, $\underline{CH_2}$CHBr), 2.08-2.21 (m, 2H, $\underline{CH_2}$CHBr), 4.65 (t, 1H, CHBr, *J* = 7.3 Hz), 4.71 (t, 1H, CHBr, *J* = 7.3 Hz);

^{13}C NMR (75.47 MHz, $CDCl_3$) δ: **pair dl (rac):** 11.89 (CH_3), 13.80 (CH_3), 22.16 (CH_2), 26.04 (CH_2), 29.30 (CH_2), 32.26 (CH_2), 50.09 (CHBr), 51.74 (CHBr), 194.35 (CO);

HRMS-ESI: calculated 320.9460 (^{79}Br) and 322.9440 (^{81}Br) $[C_9H_{16}Br_2NaO]^+$ found 320.9459 (^{79}Br) and 322.9440 (^{81}Br)

2,4-Dibromo-2-methyloctan-3-one, 3g

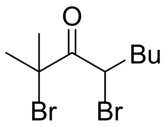

Pale yellow oil (195 mg, 65%)

^{1}H NMR (300.13 MHz, $CDCl_3$) δ: 0.91 (t, 3H, CH_3, *J* = 6.6 Hz), 1.33-1.54 (m, 4H, $2CH_2$), 1.88 (s, 3H CH_3), 2.05-2.10 (m, 5H, CH_2, CH_3), 4.94 (t, 1H, CHBr, *J* = 7.3 Hz);

^{13}C NMR (75.47 MHz, $CDCl_3$) δ: 13.78 (CH_3), 22.07 (CH_2), 29.19 (CH_3), 29.39 (CH_3), 31.00 (CH_2), 34.32 (CH_2), 45.65 (CHBr), 64.02 ($(CH_3)_2$CBr), 198.04 (CO);

HRMS-ESI: calculated 320.9460 (^{79}Br) and 322.9440 (^{81}Br) $[C_9H_{16}Br_2NaO]^+$ found 320.9452 (^{79}Br) and 322.9435 (^{81}Br)

1,1-Dibromo-3,3-dimethylbutan-2-one, 6a[9]

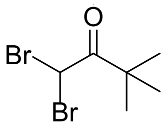

White crystals (216 mg, 84%); mp = 74-75 °C.

^{1}H NMR (300.13 MHz, $CDCl_3$) δ: 1.27 (s 9H, $3CH_3$), 6.32 (s, 1H, $CHBr_2$);

^{13}C NMR (75.47 MHz, $CDCl_3$) δ: 26.79 ($2CH_3$), 37.34 (CH_3), 43.98 ($CHBr_2$), 201.52 (CO).

2,2-Dibromo-1-phenylethanone, 6b[8]

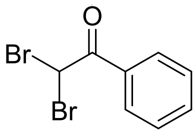

White crystals (222 mg, 80%); mp = 35-36 °C.

^{1}H NMR (300.13 MHz, $CDCl_3$) δ: 6.72 (s, 1H, $CHBr_2$), 7.49 (t, 2H, $2CH_{ar}$, J = 7.3 Hz), 7.62 (t, 1H, CH_{ar}, J = 7.3 Hz), 8.06 (d, 2H, $2CH_{ar}$, J = 7.3 Hz);

^{13}C NMR (75.47 MHz, $CDCl_3$) δ: 39.71($CHBr_2$), 128.88 (2CH), 129.61 (2CH), 134.39 (2CH), 185.88 (CO).

2,2-Dibromo-1-(p-tolyl)ethan-1-one, 6d[8]

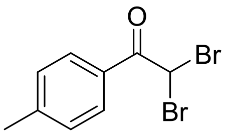

White crystals (190 mg, 65%); mp 97-98°C

^{1}H NMR (300.13 MHz, $CDCl_3$) δ: 2.44 (s, 3H, CH_3), 6.68 (s, 1H; CH), 7.29 (d, J = 8.4 Hz, 2H), 7.98 (d, J = 8.4 Hz, 2H);

^{13}C NMR (75.47 MHz, $CDCl_3$) δ: 21.81 (CH_3), 39.81 (CH), 128.10 (C), 129.64 (CH), 129.82 (C), 145.70 (C), 185.62 (CO).

2,2-Dibromo-1-(2-chlorophenyl)ethan-1-one, 6e[14]

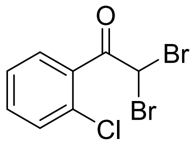

Pale yellow oil (146 mg, 47%)

^{1}H NMR (300.13 MHz, $CDCl_3$) δ: 6.78 (s, 1H; CH_2), 7.35-7.40 (m, 1H), 7.43-7.47 (m, 2H), 7.59-7.62 (m, 1H);

^{13}C NMR (75.47 MHz, $CDCl_3$) δ: 42.05 (CH), 127.20 (CH), 130.45 (CH), 130.90 (CH), 130.95 (C), 132.97 (CH), 134.07 (C), 188.76 (CO).

Pentan-3-one, 7a

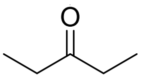

Colorless oil (28 mg, 33%)

^{1}H NMR (300.13 MHz, $CDCl_3$) δ: 0.97 (t, 6H, $2CH_3$, J = 7.3 Hz), 2.35 (t, 4H, $2CH_2$, J = 7.3 Hz).

References

1. L. F. Tietze and T. Eicher, *Reaktionen und Synthesen im organisch-chemischen Praktikum und Forschungslaboratorium*, Wiley-VCH Verlag GmbH & Co. KGaA, 2004.
2. R. M. Munavu, *J. Org. Chem.*, 1980, **45**, 3341-3343.
3. J. Villieras, M. Rambaud, R. Tarhouni and B. Kirschleger, *Synthesis*, 1981, 68-70.
4. J. M. Poirier, *Bull. Soc. Chim. Fr.*, 1982, **2**, 1.
5. J. Barluenga, L. Llavona, M. Yus and J. M. Concellon, *J. Chem. Soc., Perkin Trans. 1*, 1991.
6. M. Parvez, S. M. H. Kabir, T. S. Sorensen, F. Sun and B. Watson, *Can. J. Chem.*, 2002, **80**, 413-417.
7. I. Pravst, M. Zupan and S. Stavber, *Tetrahedron Lett.*, 2006, **47**, 4707-4710.
8. A. O. Terent'ev, S. V. Khodykin, I. B. Krylov, Y. N. Ogibin and G. I. Nikishin, *Synthesis*, 2006, 1087-1092.
9. G. I. Nikishin, L. L. Sokova and N. I. Kapustina, *Russ. Chem. Bull.*, 2010, **59**, 391-395.
10. G. I. Nikishin, L. L. Sokova and N. I. Kapustina, *Russ. Chem. Bull.*, 2013, **62**, 1214-1217.
11. K. Moriyama, T. Hamada, Y. Nakamura and H. Togo, *Chem. Commun.*, 2017, **53**, 6565-6568.
12. A. K. Macharla, R. C. Nappunni, M. R. Marri, S. Peraka and N. Nama, *Tetrahedron Lett.*, 2012, **53**, 191-195.
13. C. Wu, X. Xin, Z. Fu, L. Xie, K. Liu, Z. Wang, W. Li, Z. Yuan and W. He, *Green Chem.*, 2017, **19**, 1983.
14. P.Wu, S. Xu, H. Xu, H. Hu and W. Zhang, *Tetrahedron Lett.*, 2017, **58**, 618-621.

Copies of ^{1}H, ^{13}C NMR and HRMS spectra of synthesized products

^{1}H NMR of 2-bromopentan-3-one, **2a**

Acquisition Time (sec)	1.3518

Frequency (MHz)	300.13	Nucleus	1H	Number of Transients	1	Original Points Count	8124
Points Count	8192	Pulse Sequence	zg	Solvent	CHLOROFORM-D	Sweep Width (Hz)	6009.62
Temperature (degree C)	24.700						

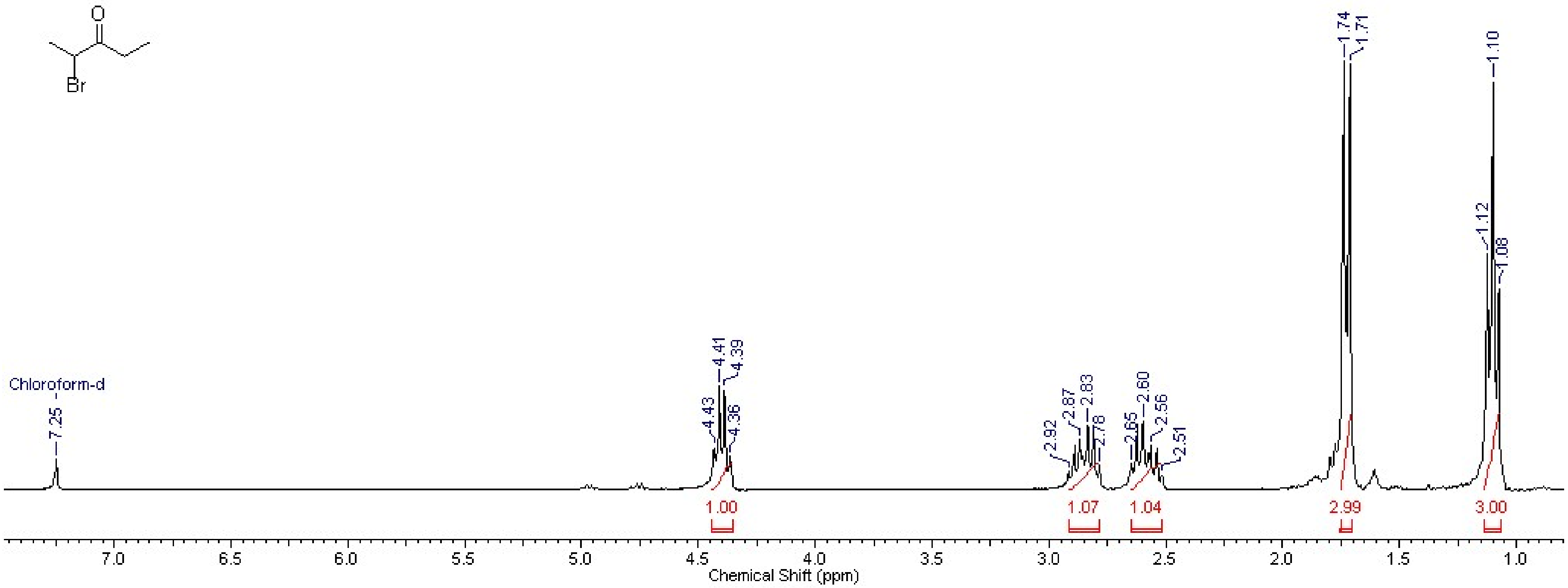

No.	(ppm)	(Hz)	Height	No.	(ppm)	(Hz)	Height
1	1.08	322.7	0.4716	10	2.78	835.5	0.0576
2	1.10	330.0	0.9535	11	2.83	850.2	0.1513
3	1.12	337.3	0.5519	12	2.87	860.5	0.1203
4	1.71	514.2	0.9941	13	2.92	875.1	0.0453
5	1.74	520.8	1.0000	14	4.36	1309.5	0.0819
6	2.51	754.8	0.0363	15	4.39	1316.8	0.2348
7	2.56	769.5	0.1055	16	4.41	1323.4	0.2433
8	2.60	779.8	0.1614	17	4.43	1330.0	0.0996
9	2.65	794.4	0.0636	18	7.25	2176.0	0.0734

No.	Annotation	(ppm)
1	Chloroform-d	7.25

^{13}C NMR of 2-bromopentan-3-one, **2a**

**Acquisition Time (sec)**	0.9006

**Frequency (MHz)**	75.48	**Nucleus**	13C	**Number of Transients**	230	**Original Points Count**	16316
**Points Count**	16384	**Pulse Sequence**	zgpg30	**Solvent**	CHLOROFORM-D	**Sweep Width (Hz)**	18115.94
**Temperature (degree C)**	24.800						

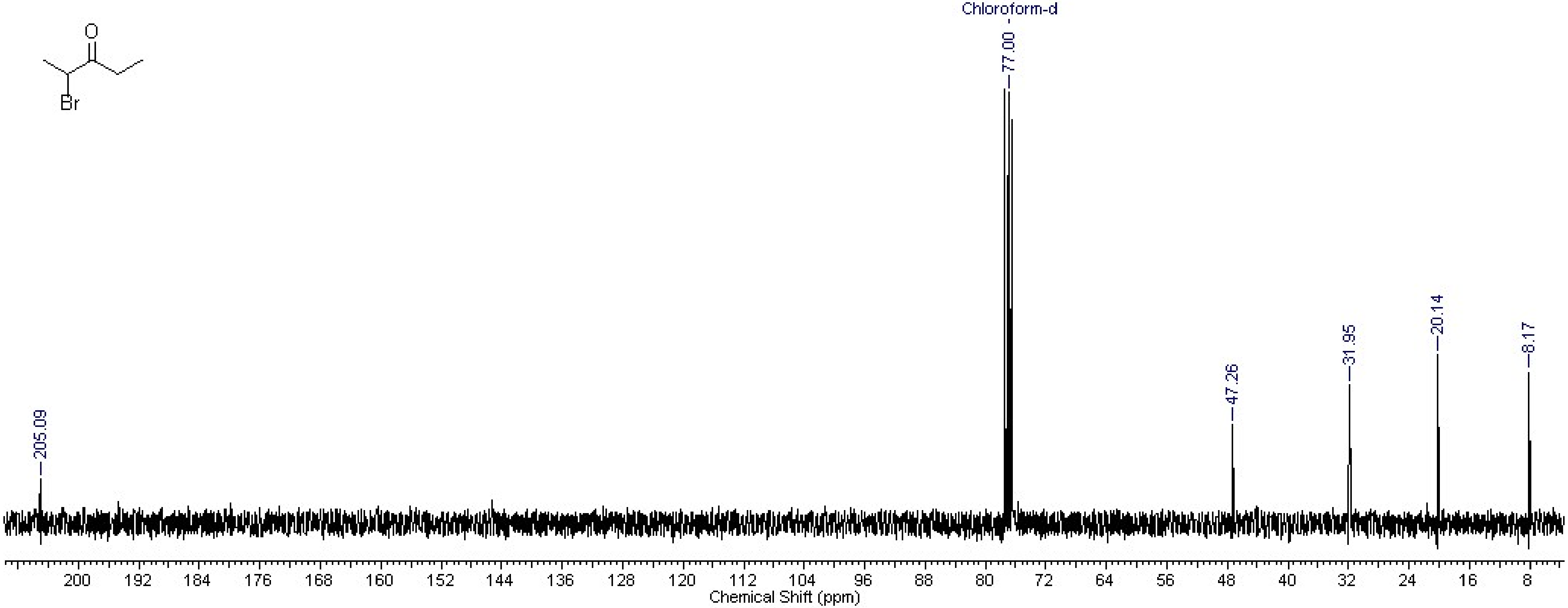

No.	(ppm)	(Hz)	Height
1	8.17	616.7	0.3504
2	20.14	1520.1	0.3913
3	31.95	2411.4	0.3224
4	47.26	3566.9	0.2309
5	77.00	5811.7	0.9903
6	205.09	15479.5	0.1063

No.	Annotation	(ppm)
1	Chloroform-d	77.00

^{1}H NMR of 3-bromoheptan-4-one, **2b**

Acquisition Time (sec)	1.3518

Frequency (MHz)	300.13	***Nucleus***	1H	***Number of Transients***	1	***Original Points Count***	8124
Points Count	8192	***Pulse Sequence***	zg	***Solvent***	CHLOROFORM-D	***Sweep Width (Hz)***	6009.62
Temperature (degree C)	27.900						

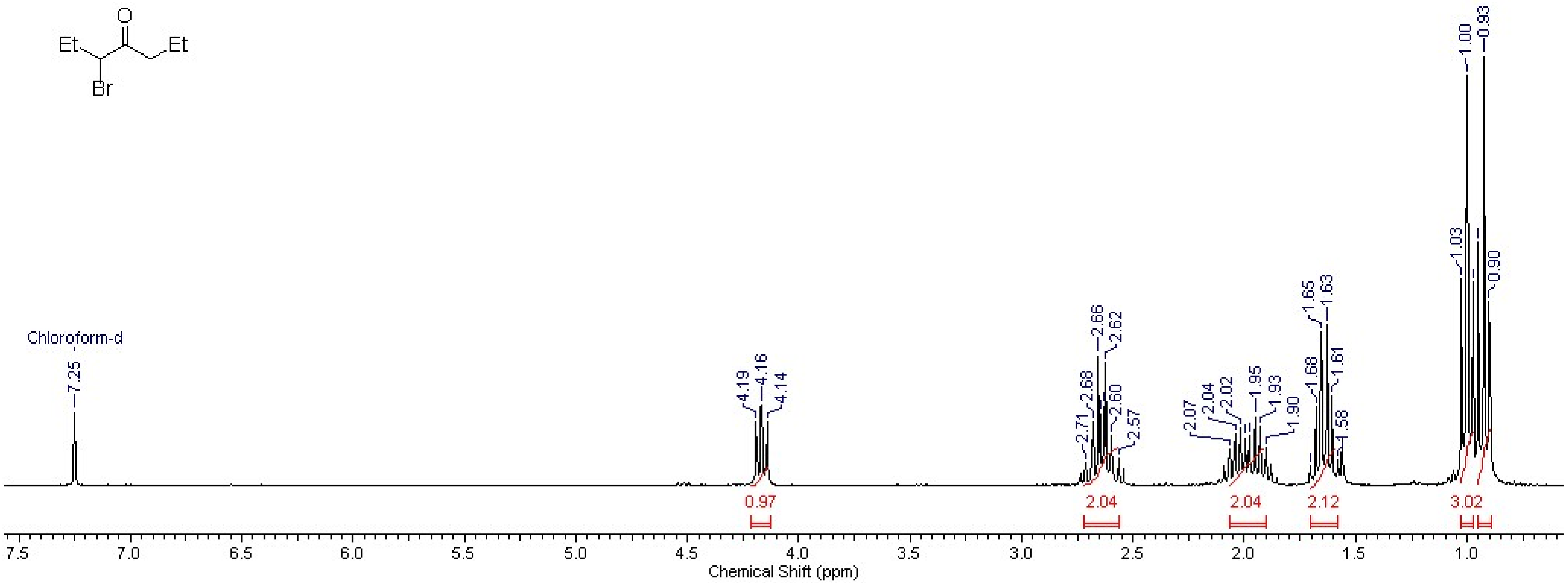

No.	(ppm)	(Hz)	Height
1	0.90	270.6	0.4289
2	0.93	277.9	1.0000
3	0.95	285.3	0.5676
4	0.98	293.3	0.4744
5	1.00	300.7	0.9563
6	1.03	308.0	0.4828
7	1.58	474.5	0.0599
8	1.61	481.9	0.2097
9	1.63	489.2	0.3750
10	1.65	496.6	0.3575
11	1.68	503.9	0.1831
12	1.70	511.2	0.0463
13	1.90	571.4	0.0906
14	1.93	578.7	0.1395
15	1.95	586.1	0.1618
16	1.98	593.4	0.1166
17	2.00	599.3	0.1118
18	2.02	605.9	0.1362
19	2.04	612.5	0.1215
20	2.07	619.8	0.0853
21	2.57	770.2	0.0641
22	2.60	780.5	0.1187
23	2.62	787.1	0.2893
24	2.63	790.0	0.1869
25	2.65	794.4	0.1686
26	2.66	797.4	0.3035
27	2.68	804.7	0.1511
28	2.71	814.2	0.0550
29	4.14	1243.4	0.1482
30	4.16	1250.0	0.1893
31	4.17	1251.5	0.1827
32	4.19	1258.1	0.1507
33	7.25	2176.0	0.1724

No.	Annotation	(ppm)
1	Chloroform-d	7.25

No.	(ppm)	Value	Absolute Value
1	[0.89 ..0.95]	3.000	2.46317e+8
2	[0.97 ..1.03]	3.022	2.48118e+8
3	[1.58 ..1.70]	2.118	1.73930e+8
4	[1.90 ..2.07]	2.044	1.67822e+8
5	[2.56 ..2.72]	2.043	1.67725e+8
6	[4.13 ..4.21]	0.974	8.00050e+7

^{13}C NMR of 3-bromoheptan-4-one, **2b**

Acquisition Time (sec)	0.9006

Frequency (MHz)	75.48	Nucleus	13C	Number of Transients	111	Original Points Count	16316
Points Count	16384	Pulse Sequence	zgpg30	Solvent	CHLOROFORM-D	Sweep Width (Hz)	18115.94
Temperature (degree C)	28.100						

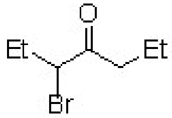

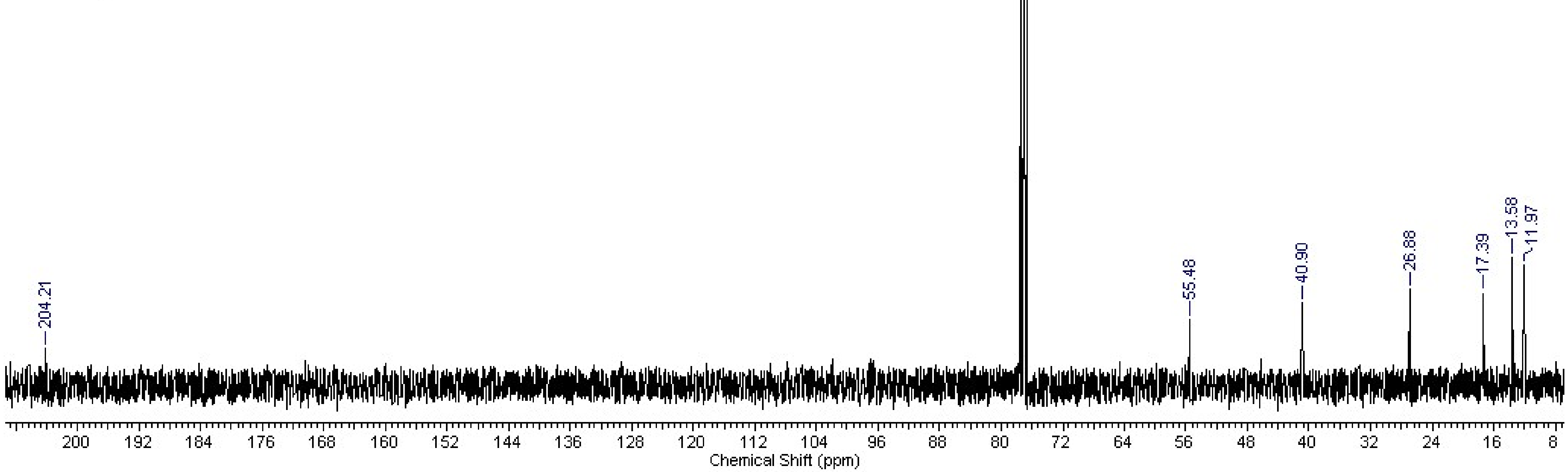

No.	(ppm)	(Hz)	Height
1	11.97	903.1	0.2666
2	13.58	1024.7	0.2838
3	17.39	1312.3	0.2039
4	26.88	2028.8	0.2108
5	40.90	3087.0	0.1821
6	55.48	4187.3	0.1457
7	77.00	5811.7	0.9564
8	204.21	15413.1	0.0807

No.	Annotation	(ppm)
1	Chloroform-d	77.00

^{1}H NMR of 4-bromononan-5-one, **2c**

Acquisition Time (sec)	1.3518

Frequency (MHz)	300.13	Nucleus	1H	Number of Transients	1	Original Points Count	8124
Points Count	65536	Pulse Sequence	zg	Solvent	CHLOROFORM-D	Sweep Width (Hz)	6009.62
Temperature (degree C)	27.500						

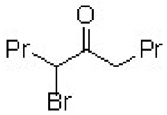

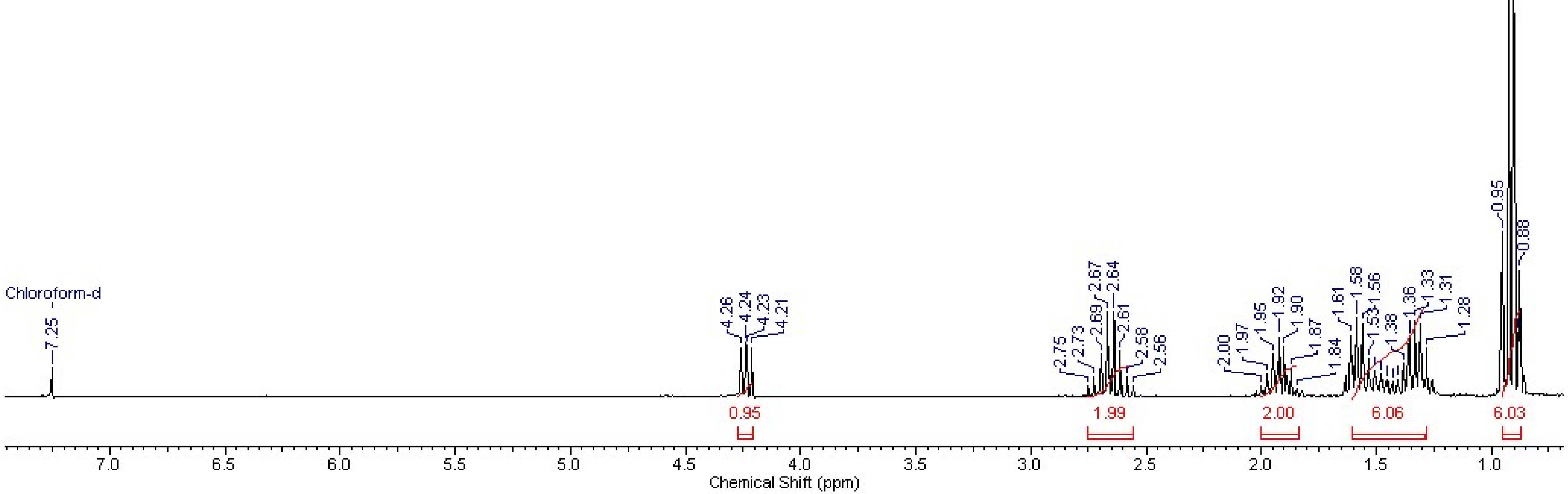

No.	(ppm)	(Hz)	Height	No.	(ppm)	(Hz)	Height	No.	(ppm)	(Hz)	Height	No.	(ppm)	(Hz)	Height
1	0.88	263.1	0.2954	11	1.41	421.8	0.0404	21	1.87	561.1	0.0651	31	2.67	801.0	0.1976
2	0.88	264.7	0.1493	12	1.43	428.9	0.0337	22	1.90	569.9	0.1174	32	2.69	808.3	0.0972
3	0.90	270.5	0.9289	13	1.45	436.1	0.0389	23	1.92	576.2	0.1391	33	2.73	818.1	0.0454
4	0.93	278.1	1.0000	14	1.48	443.6	0.0528	24	1.95	585.5	0.0992	34	2.75	825.5	0.0229
5	0.95	285.6	0.3865	15	1.50	451.2	0.0608	25	1.97	592.0	0.0524	35	4.21	1263.9	0.1148
6	1.28	384.6	0.1120	16	1.53	460.2	0.0883	26	2.00	599.8	0.0254	36	4.23	1270.4	0.1199
7	1.31	391.8	0.1711	17	1.56	467.8	0.1718	27	2.56	767.3	0.0243	37	4.24	1272.0	0.1268
8	1.33	399.5	0.1787	18	1.58	475.6	0.1836	28	2.58	774.5	0.0441	38	4.26	1278.5	0.1151
9	1.36	407.1	0.1377	19	1.61	483.0	0.1428	29	2.61	784.4	0.1049	39	7.25	2176.0	0.0659
10	1.38	414.6	0.0759	20	1.84	552.2	0.0176	30	2.64	791.6	0.1980				

No.	Annotation	(ppm)
1	Chloroform-d	7.25

^{13}C NMR of 4-bromononan-5-one, **2c**

Acquisition Time (sec)	0.9006

Frequency (MHz)	75.48	Nucleus	13C	Number of Transients	156	Original Points Count	16316
Points Count	131072	Pulse Sequence	zgpg30	Solvent	CHLOROFORM-D	Sweep Width (Hz)	18115.94
Temperature (degree C)	27.500						

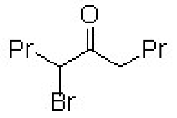

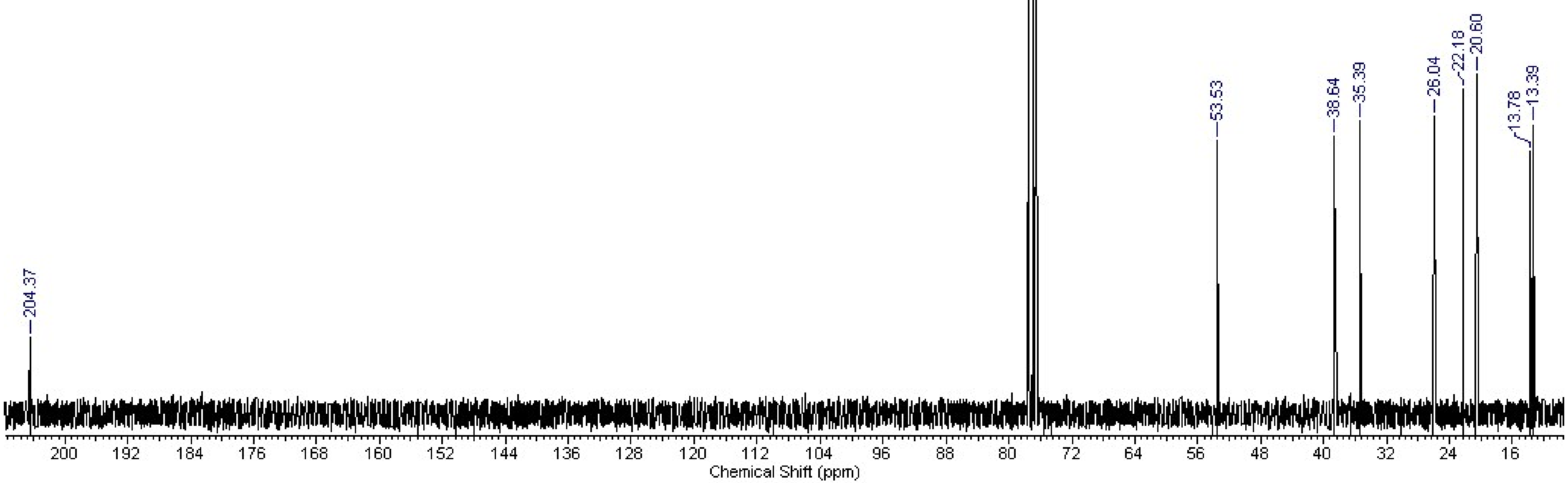

No.	(ppm)	(Hz)	Height
1	13.39	1010.6	0.6440
2	13.78	1039.9	0.5852
3	20.60	1555.1	0.7583
4	22.18	1674.3	0.7229
5	26.04	1965.7	0.6627
6	35.39	2670.7	0.6519
7	38.64	2916.1	0.6180
8	53.53	4040.6	0.6095
9	77.00	5811.7	1.0000
10	204.37	15424.8	0.1687

No.	Annotation	(ppm)
1	Chloroform-d	77.00

^{1}H NMR of 5-bromoundecan-6-one, **2d**

Acquisition Time (sec)	1.3518

Frequency (MHz)	300.13	Nucleus	1H	Number of Transients	1	Original Points Count	8124
Points Count	8192	Pulse Sequence	zg	Solvent	CHLOROFORM-D	Sweep Width (Hz)	6009.62
Temperature (degree C)	27.900						

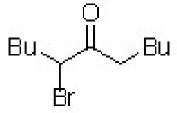

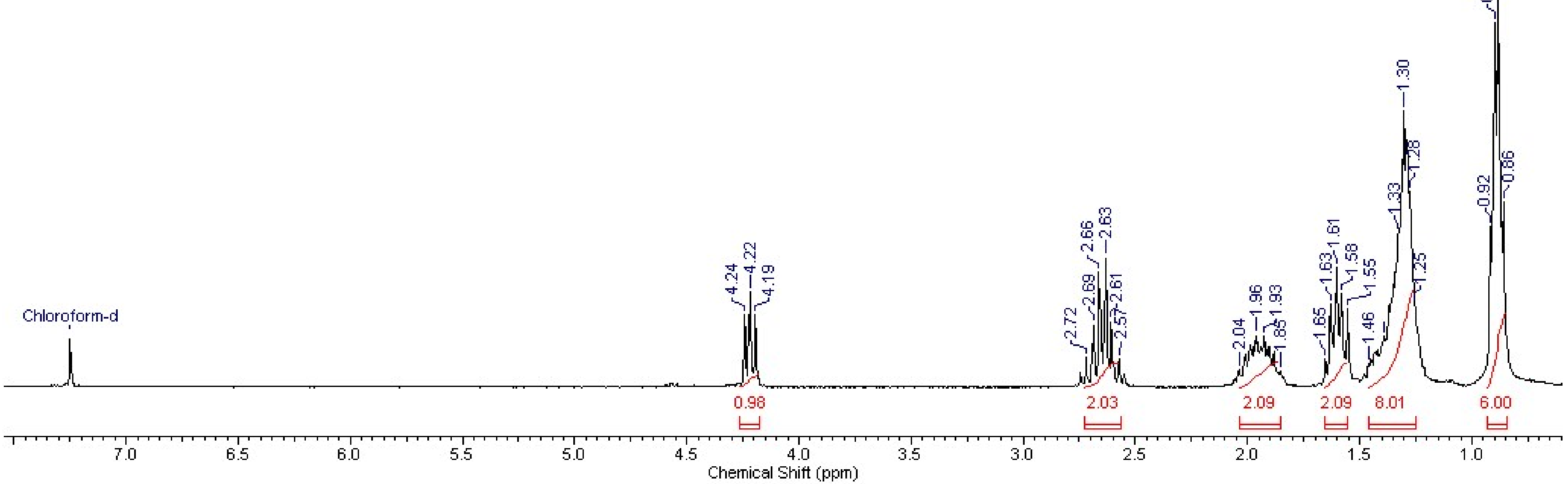

No.	(ppm)	(Hz)	Height
1	0.86	258.1	0.4292
2	0.88	264.7	1.0000
3	0.90	269.1	0.8476
4	0.92	275.7	0.3739
5	1.25	376.2	0.1949
6	1.28	382.8	0.4551
7	1.30	390.2	0.6426
8	1.33	399.7	0.3567
9	1.39	418.1	0.1203
10	1.46	438.6	0.0554
11	1.55	466.5	0.1821
12	1.58	474.5	0.2170
13	1.61	481.9	0.2779
14	1.63	489.2	0.1931
15	1.65	496.6	0.0665
16	1.85	556.0	0.0387
17	1.93	578.0	0.1182
18	1.96	587.5	0.1207
19	2.04	611.7	0.0413
20	2.57	772.4	0.0652
21	2.61	782.7	0.1524
22	2.63	790.0	0.3009
23	2.66	798.8	0.2662
24	2.69	806.9	0.1459
25	2.72	816.4	0.0694
26	4.19	1258.9	0.1673
27	4.22	1265.5	0.2208
28	4.24	1273.5	0.1681

No.	Annotation	(ppm)
1	Chloroform-d	7.25

No.	(ppm)	Value	Absolute Value
1	[0.84 ..0.93]	6.000	3.68038e+8
2	[1.25 ..1.46]	8.010	4.91373e+8
3	[1.55 ..1.65]	2.093	1.28423e+8
4	[1.85 ..2.04]	2.094	1.28475e+8
5	[2.57 ..2.73]	2.025	1.24228e+8
6	[4.17 ..4.27]	0.980	6.01175e+7

^{13}C NMR of 5-bromoundecan-6-one, **2d**

Acquisition Time (sec)	0.9006

Frequency (MHz)	75.48	Nucleus	13C	Number of Transients	230	Original Points Count	16316
Points Count	16384	Pulse Sequence	zgpg30	Solvent	CHLOROFORM-D	Sweep Width (Hz)	18115.94
Temperature (degree C)	27.900						

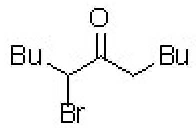

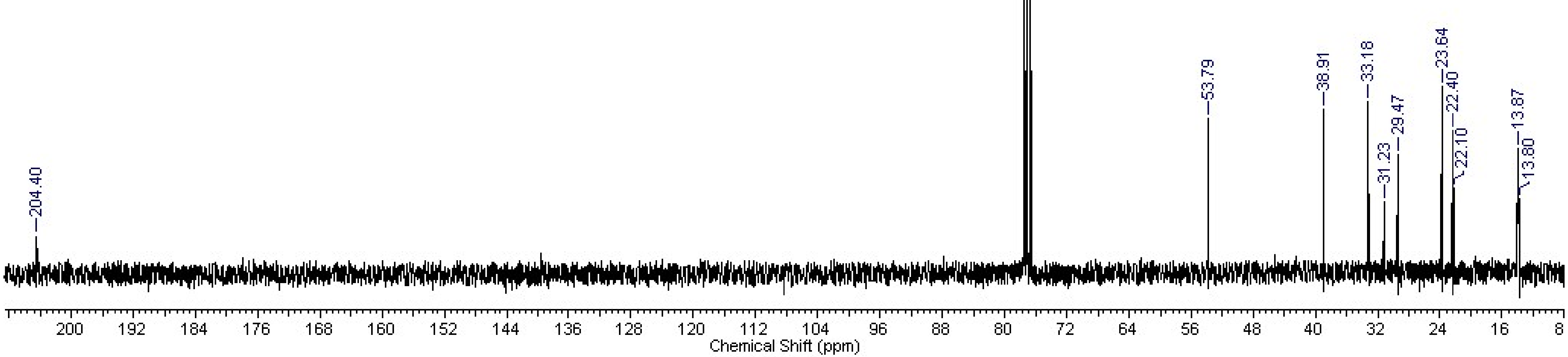

No.	(ppm)	(Hz)	Height	No.	(ppm)	(Hz)	Height
1	13.80	1041.3	0.1695	7	31.23	2357.2	0.1618
2	13.87	1046.9	0.2853	8	33.18	2504.3	0.3945
3	22.10	1668.3	0.1946	9	38.91	2936.6	0.3766
4	22.40	1690.4	0.3270	10	53.79	4060.1	0.3547
5	23.64	1784.4	0.4289	11	77.00	5811.7	1.0000
6	29.47	2224.5	0.2719	12	204.40	15427.5	0.0839

No.	Annotation	(ppm)
1	Chloroform-d	77.00

^{1}H NMR of 8-bromoheptadecan-9-one, **2e**

Acquisition Time (sec)	1.3518

Frequency (MHz)	300.13	Nucleus	1H	Number of Transients	1	Original Points Count	8124
Points Count	8192	Pulse Sequence	zg	Solvent	CHLOROFORM-D	Sweep Width (Hz)	6009.62
Temperature (degree C)	27.100						

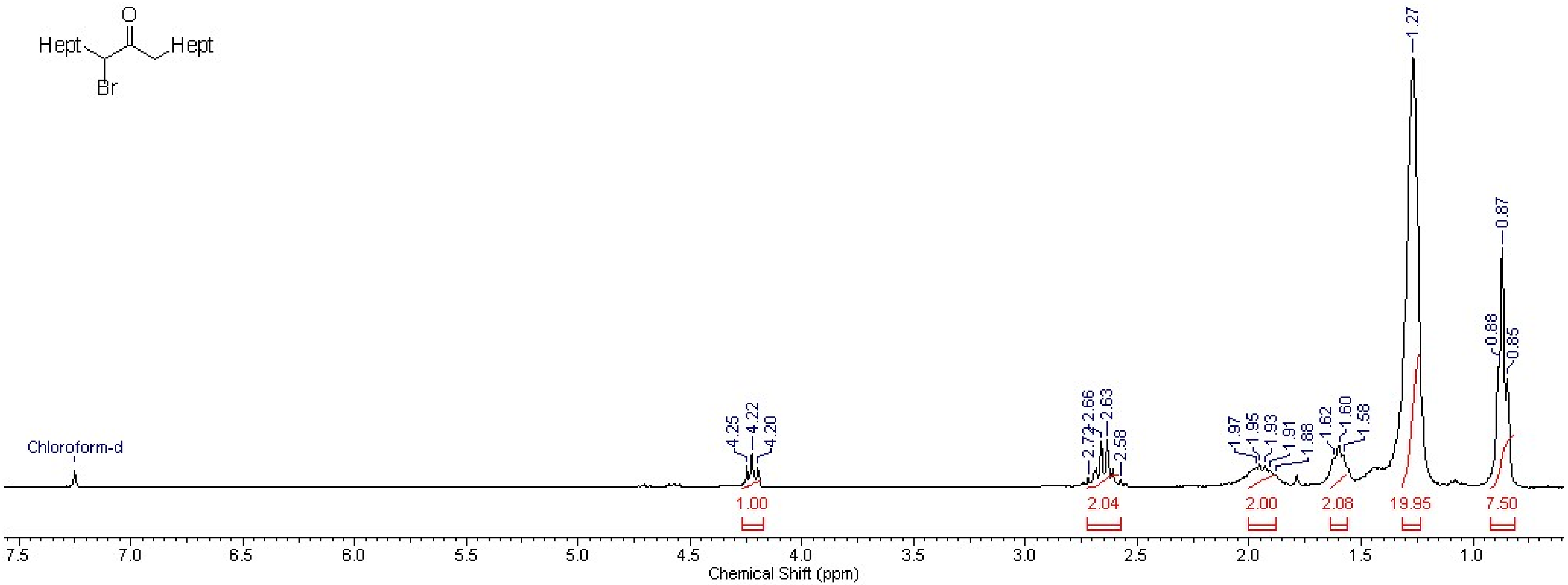

No.	(ppm)	(Hz)	Height
1	0.85	253.7	0.2537
2	0.87	260.3	0.5569
3	0.88	265.4	0.2841
4	1.27	379.9	1.0000
5	1.58	473.1	0.0770
6	1.60	479.7	0.0966
7	1.62	486.3	0.0696
8	1.88	564.8	0.0299
9	1.91	572.9	0.0407
10	1.93	579.5	0.0510

No.	(ppm)	(Hz)	Height
11	1.95	586.1	0.0544
12	1.97	591.2	0.0491
13	2.58	773.2	0.0184
14	2.63	790.8	0.1129
15	2.66	798.8	0.1083
16	2.72	815.7	0.0231
17	4.20	1259.6	0.0490
18	4.22	1266.9	0.0794
19	4.25	1274.3	0.0499

No.	Annotation	(ppm)
1	Chloroform-d	7.25

No.	(ppm)	Value	Absolute Value
1	[0.81 .. 0.92]	7.500	3.56878e+8
2	[1.23 .. 1.31]	19.954	9.49445e+8
3	[1.56 .. 1.63]	2.079	9.89329e+7
4	[1.88 .. 2.00]	2.000	9.51624e+7
5	[2.58 .. 2.72]	2.036	9.68686e+7
6	[4.17 .. 4.27]	1.000	4.75818e+7

^{13}C NMR of 8-bromoheptadecan-9-one, **2e**

Acquisition Time (sec)	0.9006

Frequency (MHz)	75.48	Nucleus	13C	Number of Transients	413	Original Points Count	16316
Points Count	262144	Pulse Sequence	zgpg30	Solvent	CHLOROFORM-D	Sweep Width (Hz)	18115.94
Temperature (degree C)	29.200						

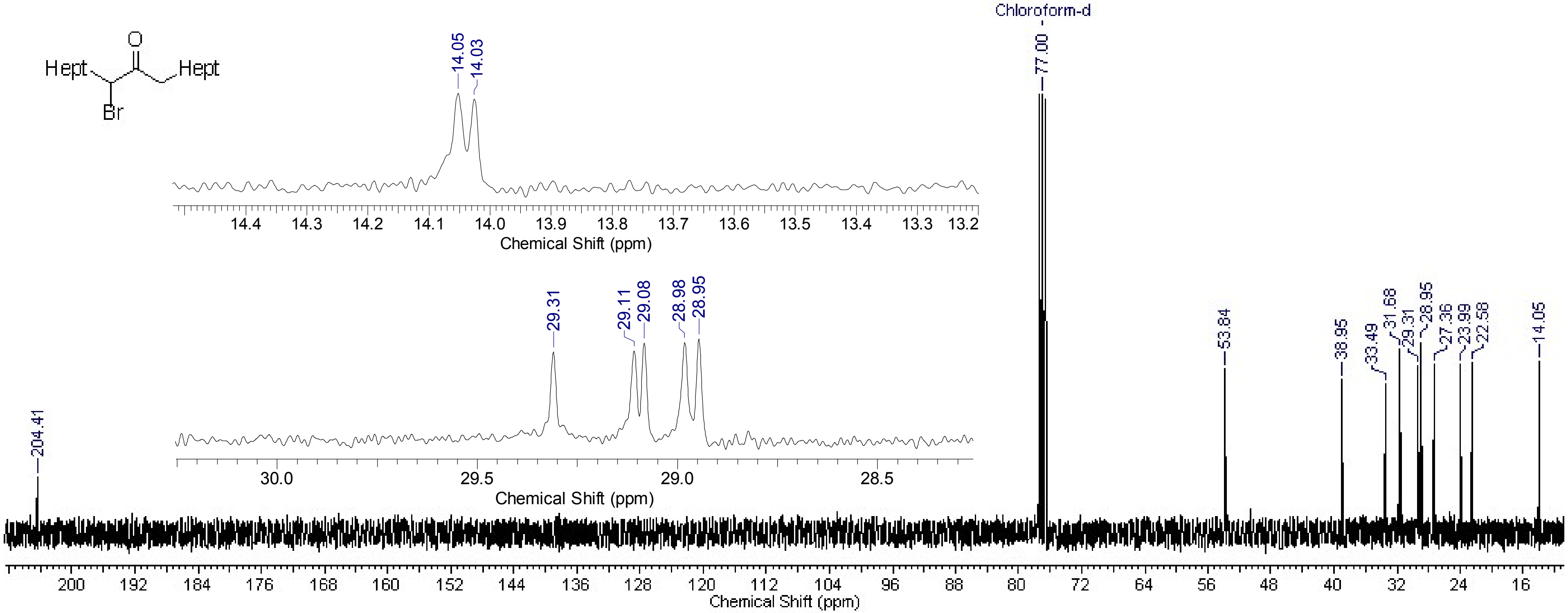

No.	(ppm)	(Hz)	Height	No.	(ppm)	(Hz)	Height
1	14.03	1058.6	0.3704	10	29.11	2197.0	0.3884
2	14.05	1060.6	0.3945	11	29.31	2212.2	0.3839
3	22.58	1704.2	0.3934	12	31.68	2391.2	0.4225
4	22.63	1707.7	0.3764	13	31.81	2400.5	0.3765
5	23.99	1810.6	0.3893	14	33.49	2527.7	0.3447
6	27.36	2064.7	0.3892	15	38.95	2939.6	0.3531
7	28.95	2184.8	0.4380	16	53.84	4063.3	0.3776
8	28.98	2187.5	0.4240	17	77.00	5811.7	0.9973
9	29.08	2195.1	0.4222	18	204.41	15427.7	0.1345

No.	Annotation	(ppm)
1	Chloroform-d	77.00

^{1}H NMR of 2-bromo-2,4-dimethylpentan-3-one, **2h**

**Acquisition Time (sec)**	1.3518						
**Frequency (MHz)**	300.13	**Nucleus**	1H	**Number of Transients**	1	**Original Points Count**	8124
**Points Count**	8192	**Pulse Sequence**	zg	**Solvent**	CHLOROFORM-D	**Sweep Width (Hz)**	6009.62
**Temperature (degree C)**	27.600						

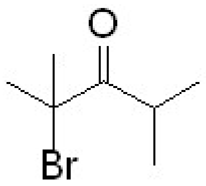

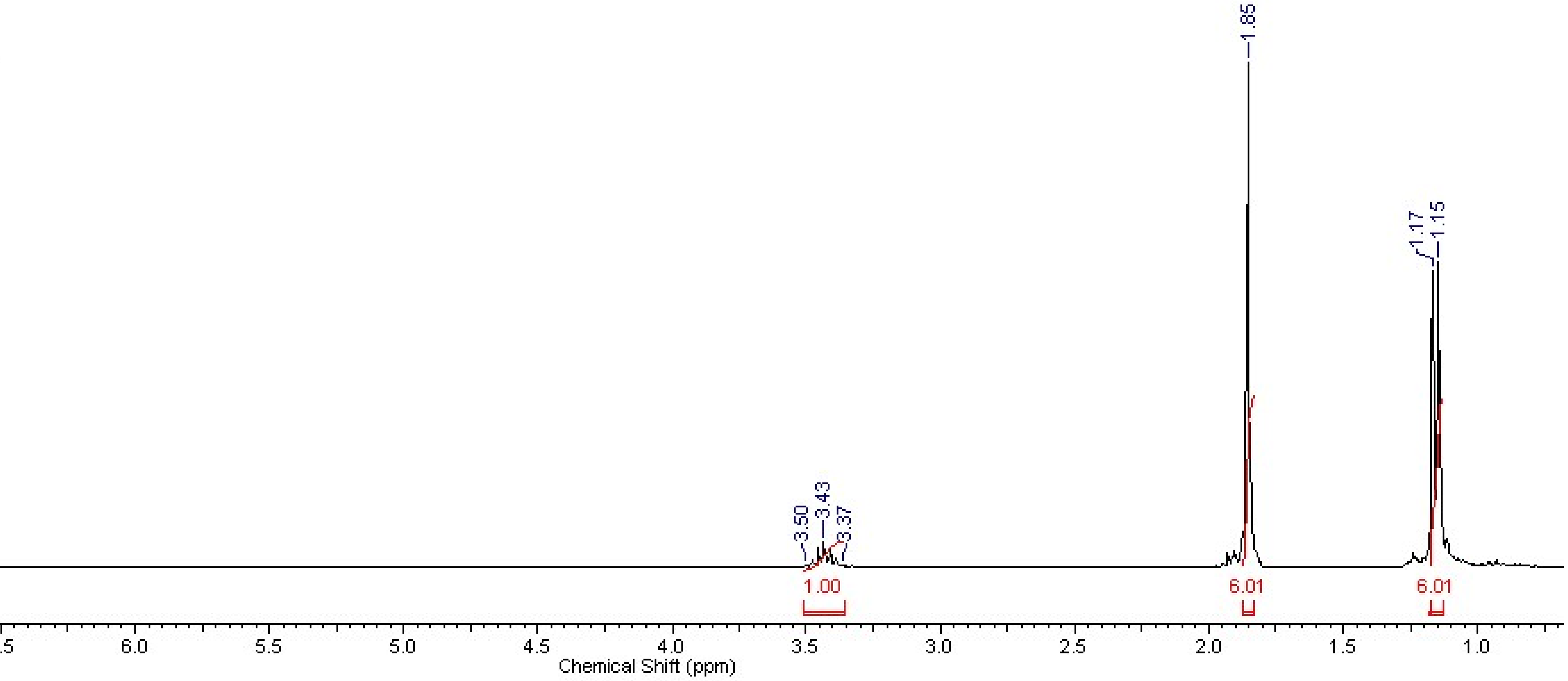

No.	(ppm)	(Hz)	Height
1	1.15	343.9	0.6056
2	1.17	350.6	0.5890
3	1.85	556.7	1.0000
4	3.37	1010.1	0.0046
5	3.43	1030.7	0.0508
6	3.50	1050.5	0.0046

No.	Annotation	(ppm)
1	Chloroform-d	7.25

No.	(ppm)	Value	Absolute Value
1	[1.13 .. 1.18]	6.013	3.93017e+8
2	[1.84 .. 1.87]	6.011	3.92933e+8
3	[3.36 .. 3.51]	1.000	6.53656e+7

^{13}C NMR of 2-bromo-2,4-dimethylpentan-3-one, **2h**

Acquisition Time (sec)	0.9006

Frequency (MHz)	75.48	Nucleus	13C	Number of Transients	307	Original Points Count	16316
Points Count	16384	Pulse Sequence	zgpg30	Solvent	CHLOROFORM-D	Sweep Width (Hz)	18115.94
Temperature (degree C)	27.500						

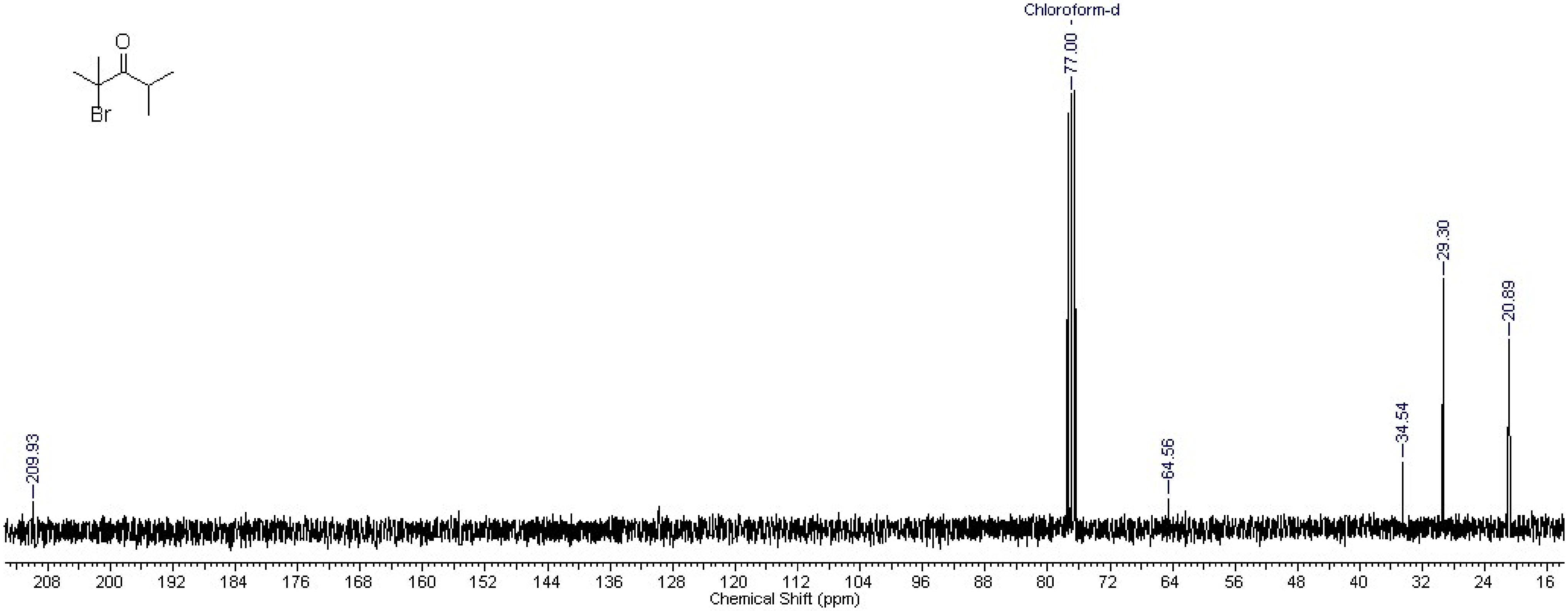

No.	(ppm)	(Hz)	Height
1	20.89	1576.5	0.4342
2	29.30	2211.2	0.5723
3	34.54	2607.1	0.1576
4	64.56	4872.9	0.0755
5	77.00	5811.7	0.9926
6	209.93	15844.4	0.0656

No.	Annotation	(ppm)
1	Chloroform-d	77.00

^{1}H NMR of mixture 3-bromononan-4-one **(2f)** и 5-bromononan-4-one **(2f’)** 1:1

Acquisition Time (sec)	1.3518

Frequency (MHz)	300.13	***Nucleus***	1H	***Number of Transients***	1	***Original Points Count***	8124
Points Count	131072	***Pulse Sequence***	zg	***Solvent***	CHLOROFORM-D	***Sweep Width (Hz)***	6009.62
Temperature (degree C)	26.700						

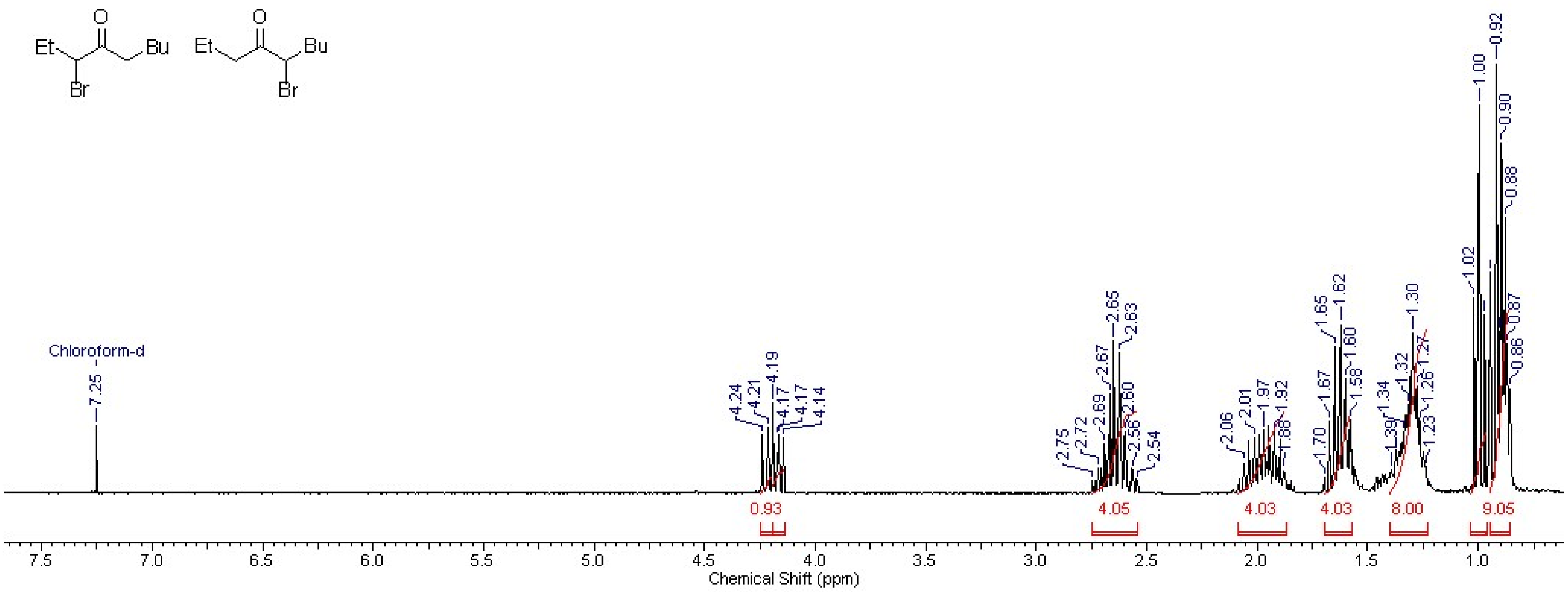

No.	(ppm)	(Hz)	Height
1	0.86	257.0	0.2431
2	0.87	261.0	0.3479
3	0.88	264.0	0.6413
4	0.90	268.7	0.8133
5	0.90	270.6	0.3775
6	0.92	276.5	1.0000
7	0.95	283.9	0.5131
8	0.97	291.6	0.4163
9	1.00	298.9	0.9031
10	1.02	306.2	0.4541
11	1.23	370.2	0.0616
12	1.26	379.5	0.1669

No.	(ppm)	(Hz)	Height
13	1.27	382.3	0.2486
14	1.30	389.5	0.3720
15	1.32	395.0	0.2074
16	1.34	401.9	0.1449
17	1.37	411.6	0.1008
18	1.39	417.6	0.0545
19	1.58	474.6	0.1712
20	1.60	480.0	0.2672
21	1.62	487.3	0.3919
22	1.65	494.6	0.3410
23	1.67	502.0	0.1682
24	1.70	509.4	0.0364

No.	(ppm)	(Hz)	Height
25	1.88	564.7	0.0529
26	1.92	576.8	0.1431
27	1.97	591.6	0.1484
28	2.01	603.7	0.1291
29	2.06	618.2	0.0715
30	2.54	762.4	0.0291
31	2.56	769.5	0.0538
32	2.60	779.6	0.1428
33	2.63	788.3	0.3253
34	2.65	795.4	0.3535
35	2.67	799.9	0.2309
36	2.69	807.3	0.1139

No.	(ppm)	(Hz)	Height
37	2.72	817.0	0.0600
38	2.75	824.4	0.0300
39	4.14	1243.8	0.1283
40	4.17	1250.2	0.1358
41	4.17	1251.8	0.1310
42	4.19	1257.7	0.2112
43	4.21	1264.0	0.1523
44	4.22	1265.5	0.1451
45	4.24	1272.0	0.1370
46	7.25	2176.0	0.1574

No.	Annotation	(ppm)
1	Chloroform-d	7.25

^{13}C NMR of mixture 3-bromononan-4-one **(2f)** и 5-bromononan-4-one **(2f')** 1:1

Acquisition Time (sec)	0.9006

Frequency (MHz)	75.48	***Nucleus***	13C	***Number of Transients***	592	***Original Points Count***	16316
Points Count	16384	***Pulse Sequence***	zgpg30	***Solvent***	CHLOROFORM-D	***Sweep Width (Hz)***	18115.94
Temperature (degree C)	26.700						

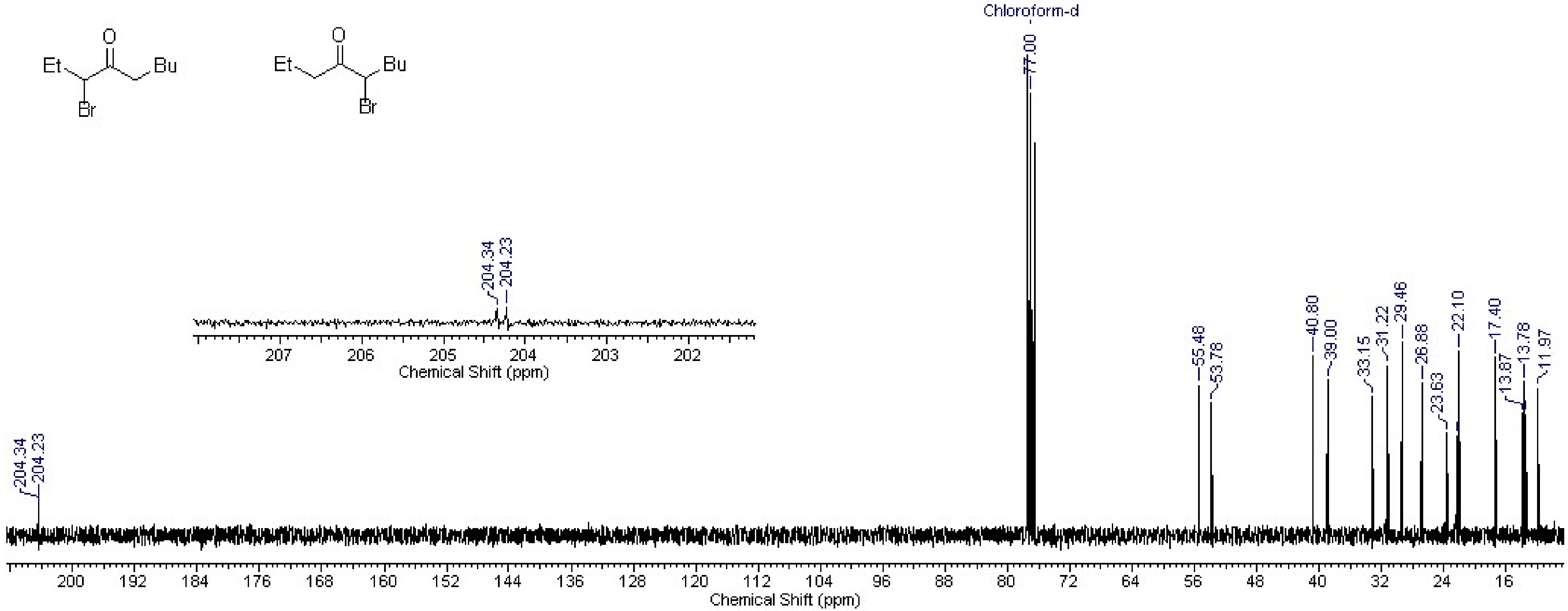

No.	(ppm)	(Hz)	Height
1	11.97	903.1	0.3044
2	13.58	1024.7	0.2510
3	13.78	1040.2	0.3174
4	13.87	1046.9	0.2526
5	17.40	1313.4	0.3686
6	22.10	1668.3	0.3816
7	22.40	1690.4	0.2065
8	23.63	1783.3	0.2131
9	26.88	2028.8	0.3152
10	29.46	2223.4	0.4005

No.	(ppm)	(Hz)	Height
11	31.22	2356.1	0.3519
12	33.15	2502.1	0.2880
13	39.00	2943.3	0.3224
14	40.80	3079.3	0.3734
15	53.78	4059.0	0.2757
16	55.48	4187.3	0.3109
17	77.00	5811.7	0.9184
18	204.23	15414.2	0.0673
19	204.34	15423.1	0.0586

No.	Annotation	(ppm)
1	Chloroform-d	77.00

^{1}H NMR of mixture 2-bromo-2-methyloctan-3-one **(2g)** и 4-bromo-2-methyloctan-3-one **(2g')** 2:1

Acquisition Time (sec)	1.3518

Frequency (MHz)	300.13	***Nucleus***	1H	***Number of Transients***	1	***Original Points Count***	8124
Points Count	65536	***Pulse Sequence***	zg	***Solvent***	CHLOROFORM-D	***Sweep Width (Hz)***	6009.62
Temperature (degree C)	26.600						

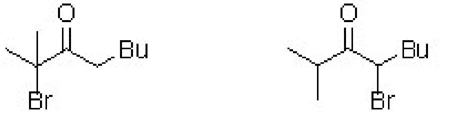

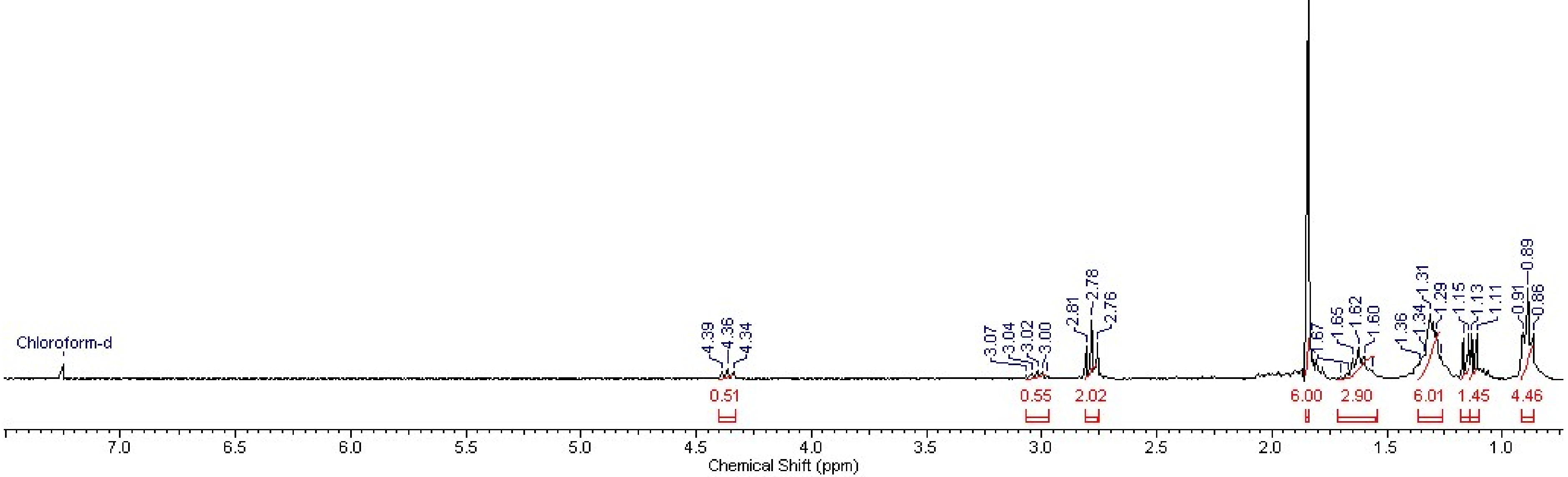

No.	(ppm)	(Hz)	Height	No.	(ppm)	(Hz)	Height	No.	(ppm)	(Hz)	Height
1	0.86	259.6	0.1040	11	1.34	402.8	0.0566	21	2.78	835.0	0.1400
2	0.89	266.4	0.2110	12	1.36	408.5	0.0360	22	2.81	842.3	0.0783
3	0.91	273.2	0.1073	13	1.56	469.7	0.0209	23	2.97	892.5	0.0080
4	1.11	332.8	0.1053	14	1.60	480.0	0.0533	24	3.00	899.3	0.0158
5	1.13	339.7	0.1047	15	1.62	487.1	0.0735	25	3.02	906.2	0.0191
6	1.15	344.0	0.1057	16	1.65	494.2	0.0496	26	3.04	913.1	0.0149
7	1.17	350.7	0.0958	17	1.67	501.3	0.0152	27	3.07	919.9	0.0087
8	1.26	379.6	0.0525	18	1.70	510.2	0.0056	28	4.34	1302.2	0.0162
9	1.29	386.5	0.1081	19	1.84	552.9	1.0000	29	4.36	1309.7	0.0262
10	1.31	393.8	0.1528	20	2.76	827.6	0.0847	30	4.39	1316.6	0.0160

No.	Annotation	(ppm)
1	Chloroform-d	7.25

^{13}C NMR of mixture 2-bromo-2-methyloctan-3-one **(2g)** и 4-bromo-2-methyloctan-3-one **(2g')** 2:1

Acquisition Time (sec)	0.9006

Frequency (MHz)	75.48	Nucleus	13C	Number of Transients	223	Original Points Count	16316
Points Count	131072	Pulse Sequence	zgpg30	Solvent	CHLOROFORM-D	Sweep Width (Hz)	18115.94
Temperature (degree C)	27.400						

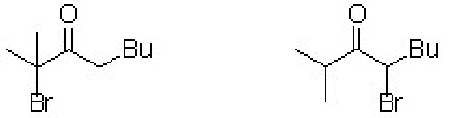

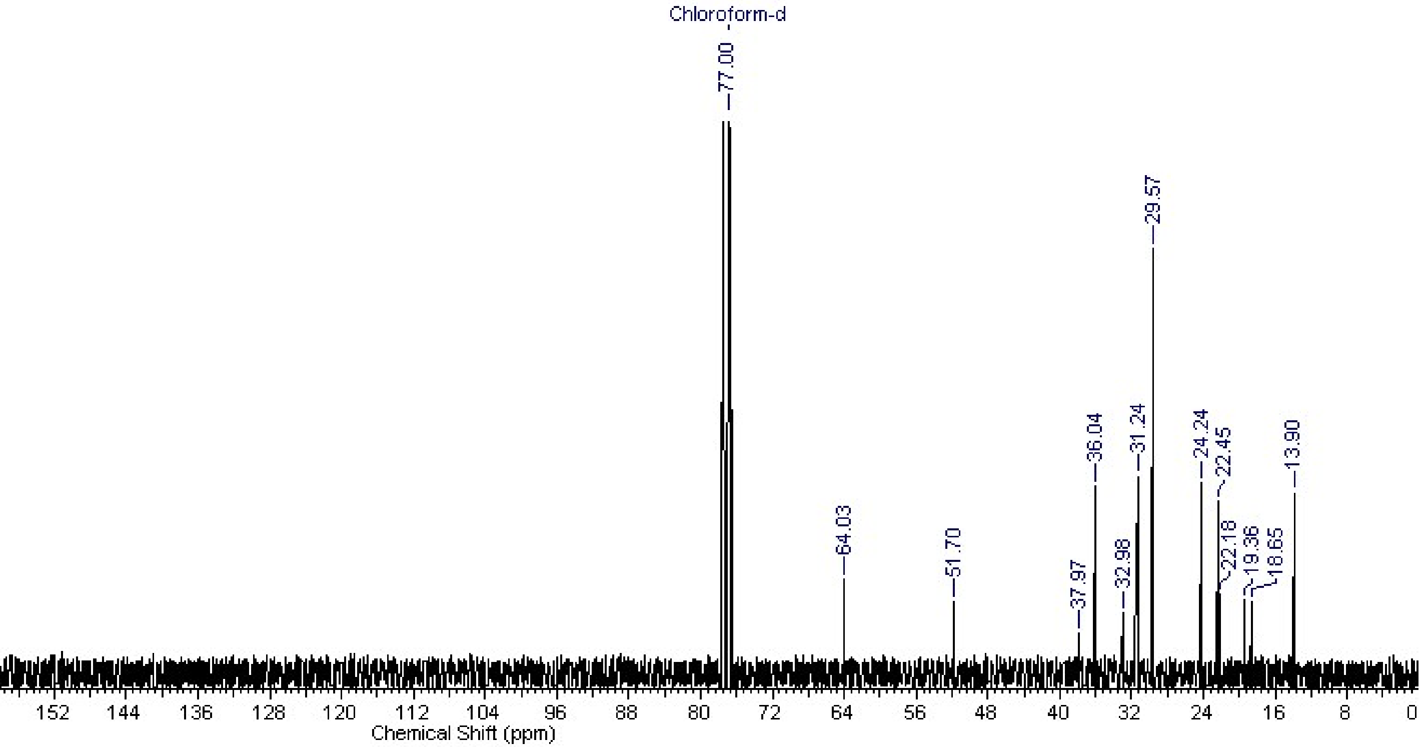

No.	(ppm)	(Hz)	Height
1	13.81	1042.0	0.1093
2	13.90	1049.3	0.3144
3	18.65	1407.3	0.1297
4	19.36	1460.9	0.1311
5	22.18	1673.9	0.1417
6	22.45	1694.5	0.3016
7	24.24	1829.7	0.3332
8	29.57	2232.2	0.7374
9	31.24	2357.8	0.3446

No.	(ppm)	(Hz)	Height
10	32.98	2489.5	0.1091
11	36.04	2719.8	0.3280
12	37.97	2865.9	0.0754
13	51.70	3902.2	0.1295
14	64.03	4832.5	0.1667
15	77.00	5811.7	1.0000
16	205.75	15529.0	0.1248
17	207.63	15670.9	0.0545

No.	Annotation	(ppm)
1	Chloroform-d	77.00

^{1}H NMR of 1-bromo-3,3-dimethylbutan-2-one, **5a**

Acquisition Time (sec)	1.3518

Frequency (MHz)	300.13	***Nucleus***	1H	***Number of Transients***	1	***Original Points Count***	8124
Points Count	8192	***Pulse Sequence***	zg	***Solvent***	CHLOROFORM-D	***Sweep Width (Hz)***	6009.62
Temperature (degree C)	26.600						

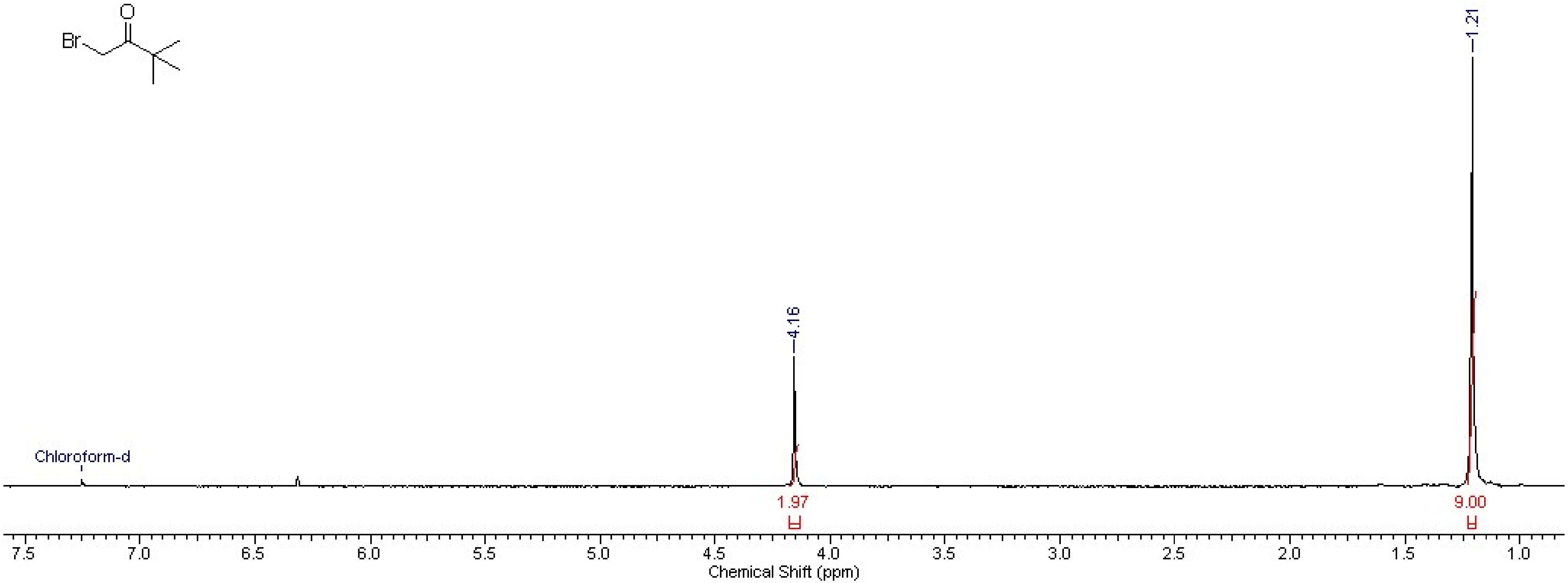

No.	(ppm)	(Hz)	Height
1	1.21	363.0	1.0000
2	4.16	1247.1	0.3018

No.	Annotation	(ppm)
1	Chloroform-d	7.25

No.	(ppm)	Value	Absolute Value
1	[1.19 .. 1.22]	9.000	9.32046e+8
2	[4.13 .. 4.17]	1.974	2.04410e+8

^{13}C NMR of 1-bromo-3,3-dimethylbutan-2-one, **5a**

**Acquisition Time (sec)**	0.9006						
**Frequency (MHz)**	75.48	**Nucleus**	13C	**Number of Transients**	111	**Original Points Count**	16316
**Points Count**	16384	**Pulse Sequence**	zgpg30	**Solvent**	CHLOROFORM-D	**Sweep Width (Hz)**	18115.94
**Temperature (degree C)**	27.500						

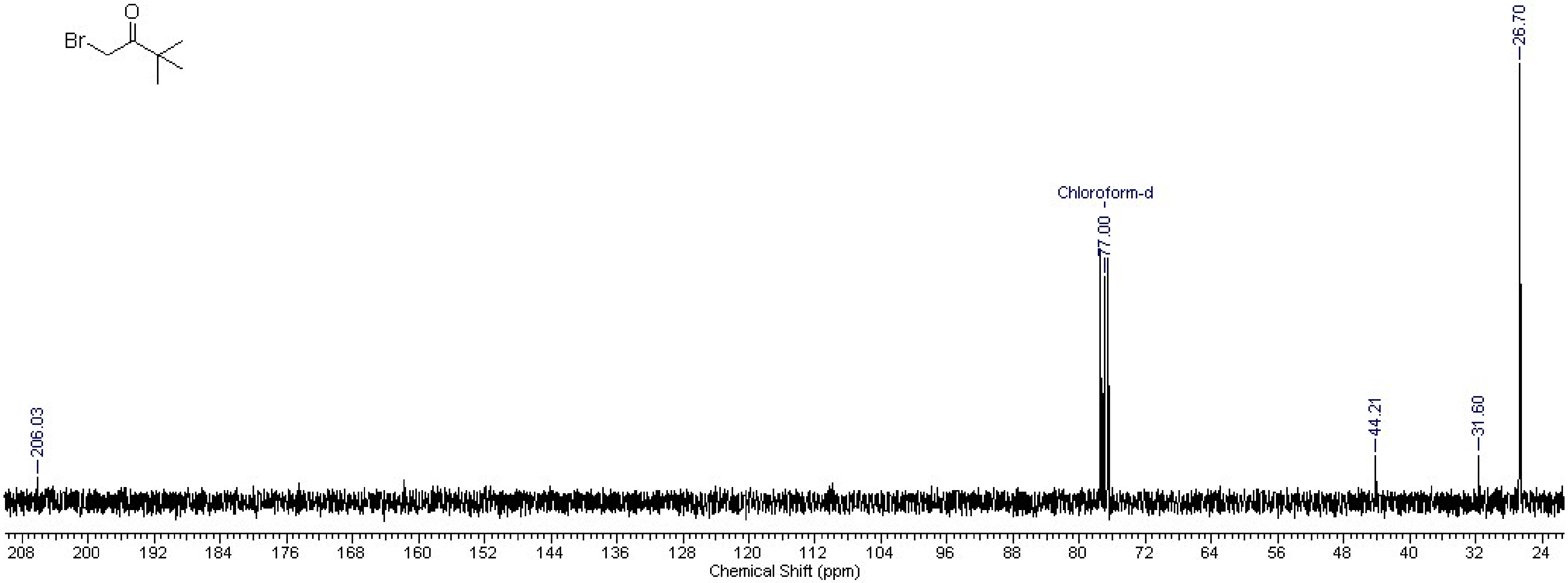

No.	(ppm)	(Hz)	Height
1	26.70	2015.5	1.0000
2	31.60	2384.9	0.1107
3	44.21	3336.9	0.1075
4	77.00	5811.7	0.5177
5	206.03	15550.2	0.0606

No.	Annotation	(ppm)
1	Chloroform-d	77.00

^{1}H NMR of 2-bromo-1-phenylethanone, **5b**

Acquisition Time (sec)	1.3518

Frequency (MHz)	300.13	Nucleus	1H	Number of Transients	1	Original Points Count	8124
Points Count	8192	Pulse Sequence	zg	Solvent	CHLOROFORM-D	Sweep Width (Hz)	6009.62
Temperature (degree C)	26.600						

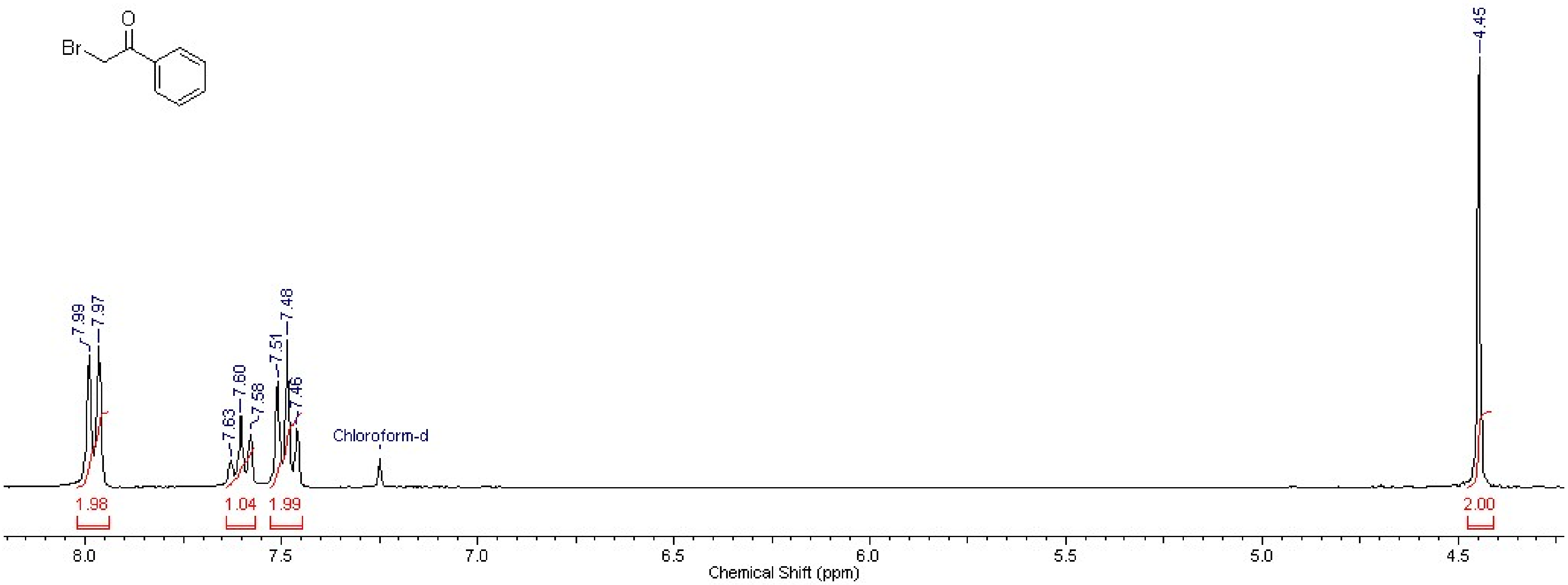

No.	(ppm)	(Hz)	Height
1	4.45	1334.4	1.0000
2	7.46	2239.1	0.1383
3	7.48	2246.4	0.3455
4	7.51	2253.7	0.2467
5	7.58	2275.0	0.1261
6	7.60	2282.3	0.1664
7	7.63	2289.7	0.0632
8	7.97	2390.9	0.3297
9	7.99	2398.3	0.3079

No.	Annotation	(ppm)
1	Chloroform-d	7.25

No.	(ppm)	Value	Absolute Value
1	[4.41 .. 4.48]	2.000	3.26962e+8
2	[7.45 .. 7.53]	1.986	3.24704e+8
3	[7.57 .. 7.64]	1.042	1.70354e+8
4	[7.94 .. 8.02]	1.985	3.24433e+8

^{13}C NMR of 2-bromo-1-phenylethanone, **5b**

Acquisition Time (sec)	0.9006

Frequency (MHz)	75.48	Nucleus	13C	Number of Transients	330	Original Points Count	16316
Points Count	16384	Pulse Sequence	zgpg30	Solvent	CHLOROFORM-D	Sweep Width (Hz)	18115.94
Temperature (degree C)	24.700						

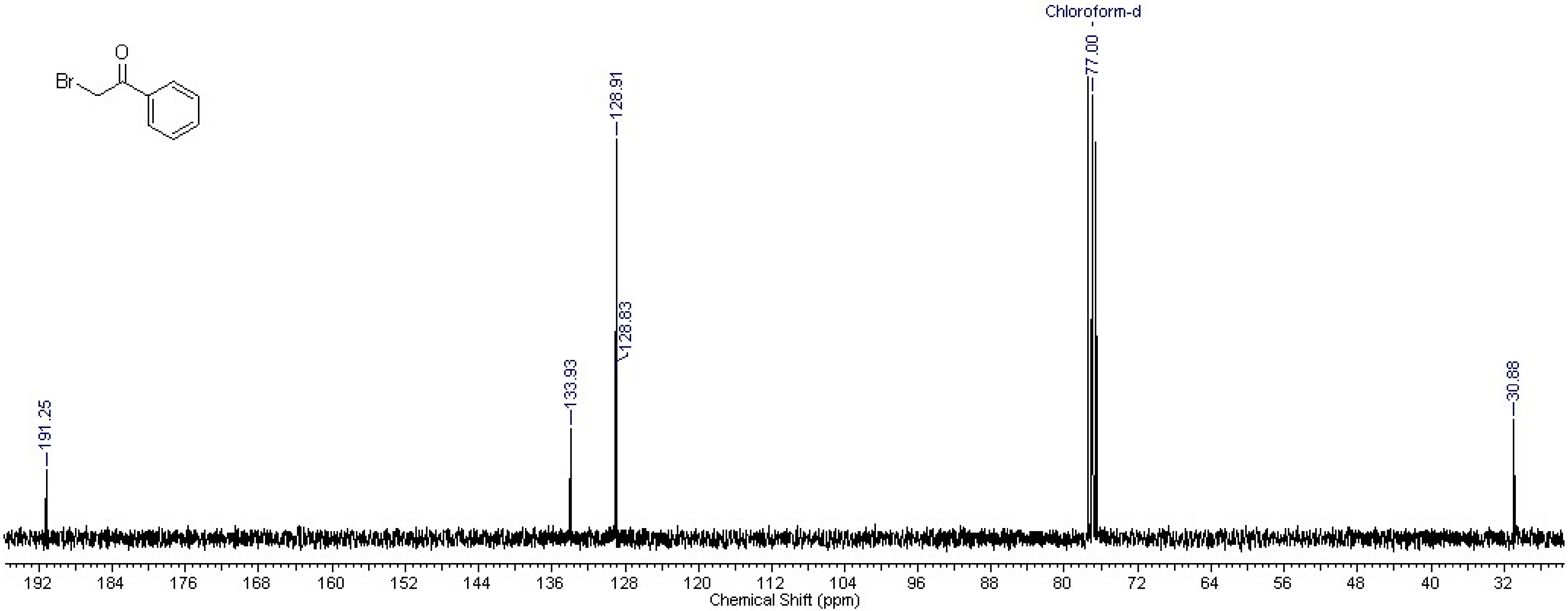

No.	(ppm)	(Hz)	Height
1	30.88	2330.7	0.2519
2	77.00	5811.7	0.9618
3	128.83	9723.9	0.3605
4	128.91	9729.4	0.8657
5	133.93	10108.7	0.2335
6	191.25	14434.5	0.1443

No.	Annotation	(ppm)
1	Chloroform-d	77.00

^{1}H NMR of 2-bromo-1-phenylhexan-1-one, **5c**

Acquisition Time (sec)	1.3518

Frequency (MHz)	300.13	Nucleus	1H	Number of Transients	1	Original Points Count	8124
Points Count	8192	Pulse Sequence	zg	Solvent	CHLOROFORM-D	Sweep Width (Hz)	6009.62
Temperature (degree C)	24.900						

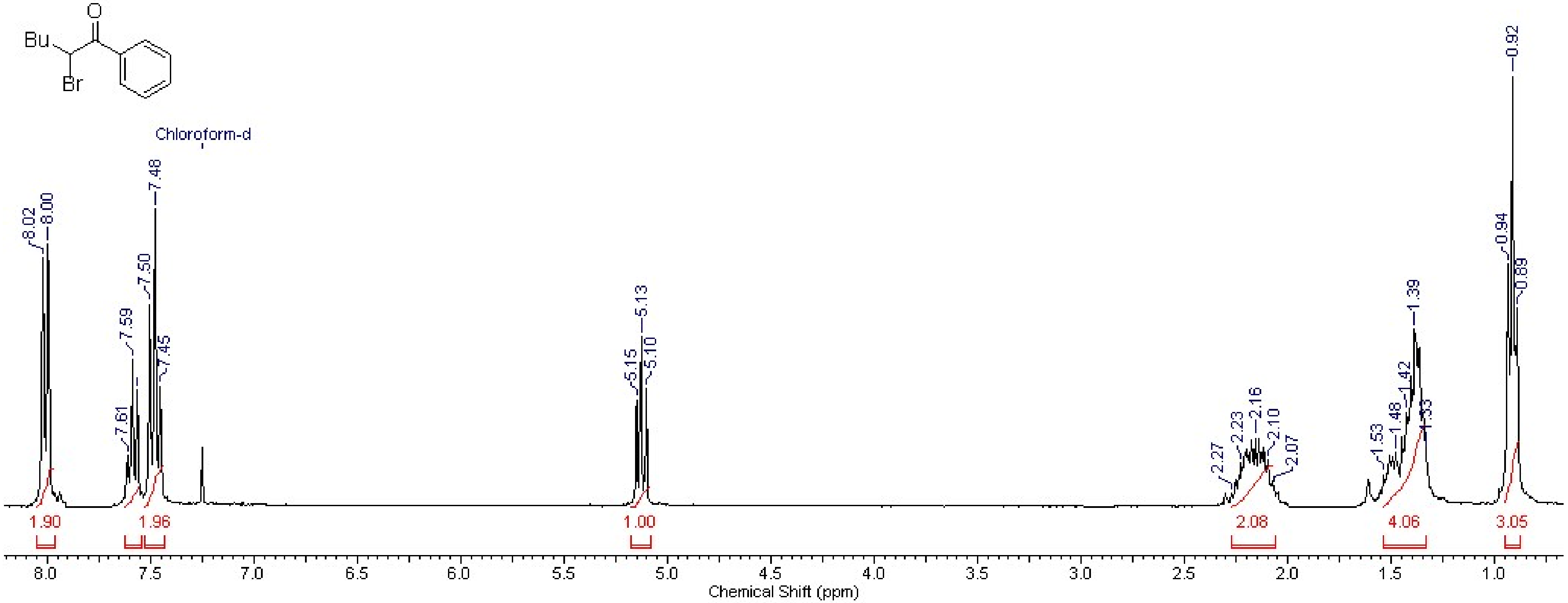

No.	(ppm)	(Hz)	Height
1	0.89	267.6	0.4603
2	0.92	275.0	1.0000
3	0.94	281.6	0.5633
4	1.33	399.7	0.1299
5	1.39	416.6	0.4103
6	1.42	427.6	0.2195
7	1.48	443.7	0.1241
8	1.53	460.6	0.0753
9	2.07	619.8	0.0487
10	2.10	629.4	0.1089
11	2.16	647.0	0.1592
12	2.23	668.2	0.1013

No.	(ppm)	(Hz)	Height
13	2.27	682.2	0.0311
14	5.10	1531.8	0.2741
15	5.13	1538.4	0.3953
16	5.15	1545.7	0.2481
17	7.45	2236.9	0.2799
18	7.48	2244.2	0.6900
19	7.50	2252.3	0.4703
20	7.56	2269.9	0.2704
21	7.59	2277.2	0.3422
22	7.61	2284.5	0.1193
23	8.00	2400.5	0.6100
24	8.02	2407.8	0.5769

No.	Annotation	(ppm)
1	Chloroform-d	7.25

No.	(ppm)	Value	Absolute Value
1	[0.88 .. 0.95]	3.049	2.62274e+8
2	[1.33 .. 1.54]	4.057	3.49017e+8
3	[2.06 .. 2.27]	2.084	1.79310e+8
4	[5.08 .. 5.18]	1.000	8.60325e+7
5	[7.43 .. 7.52]	1.965	1.69016e+8
6	[7.54 .. 7.63]	0.997	8.57328e+7
7	[7.96 .. 8.05]	1.898	1.63306e+8

^{13}C NMR of 2-bromo-1-phenylhexan-1-one, **5c**

Acquisition Time (sec)	0.9006

Frequency (MHz)	75.48	Nucleus	13C	Number of Transients	526	Original Points Count	16316
Points Count	16384	Pulse Sequence	zgpg30	Solvent	CHLOROFORM-D	Sweep Width (Hz)	18115.94
Temperature (degree C)	24.900						

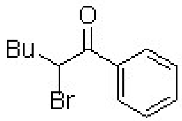

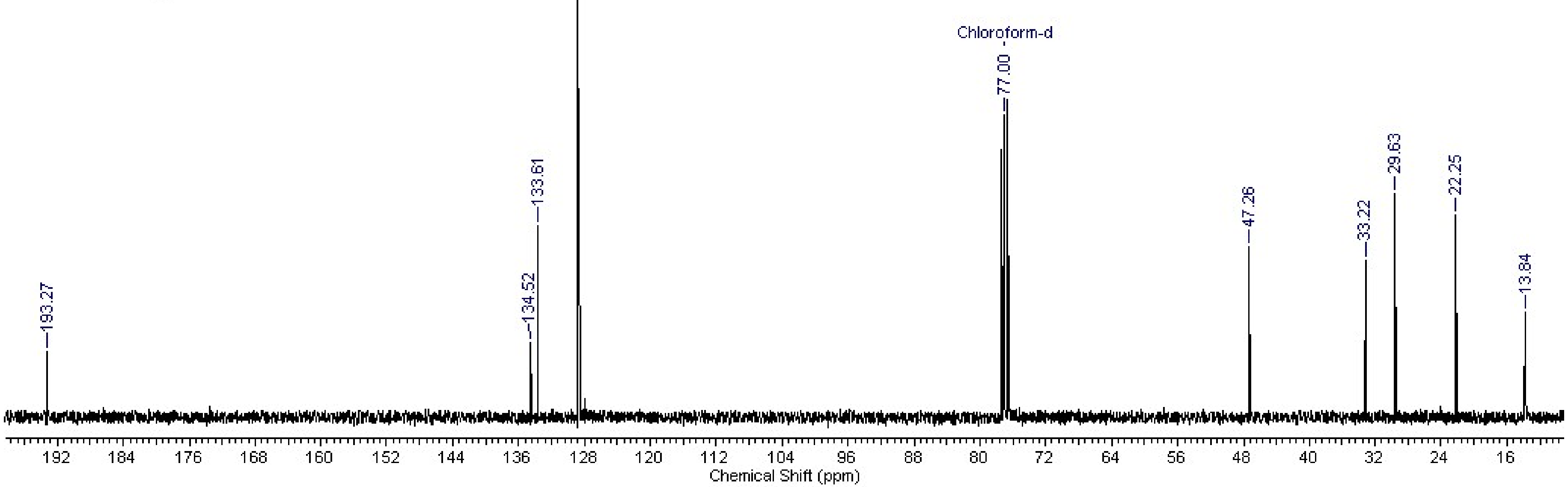

No.	(ppm)	(Hz)	Height
1	13.84	1044.7	0.2368
2	22.25	1679.4	0.4588
3	29.63	2236.7	0.5084
4	33.22	2507.6	0.3557
5	47.26	3566.9	0.3856
6	77.00	5811.7	0.6879
7	128.73	9716.2	0.4975
8	128.80	9721.7	1.0000
9	133.61	10084.4	0.4361
10	134.52	10152.9	0.1687
11	193.27	14587.1	0.1492

No.	Annotation	(ppm)
1	Chloroform-d	77.00

^{1}H NMR of 2-bromo-1-(p-tolyl)ethan-1-one, **5d**

**Acquisition Time (sec)**	2.7150

**Frequency (MHz)**	300.13	**Nucleus**	1H	**Number of Transients**	1	**Original Points Count**	16316
**Points Count**	16384	**Pulse Sequence**	zg	**Solvent**	CHLOROFORM-D	**Sweep Width (Hz)**	6009.62
**Temperature (degree C)**	27.015						

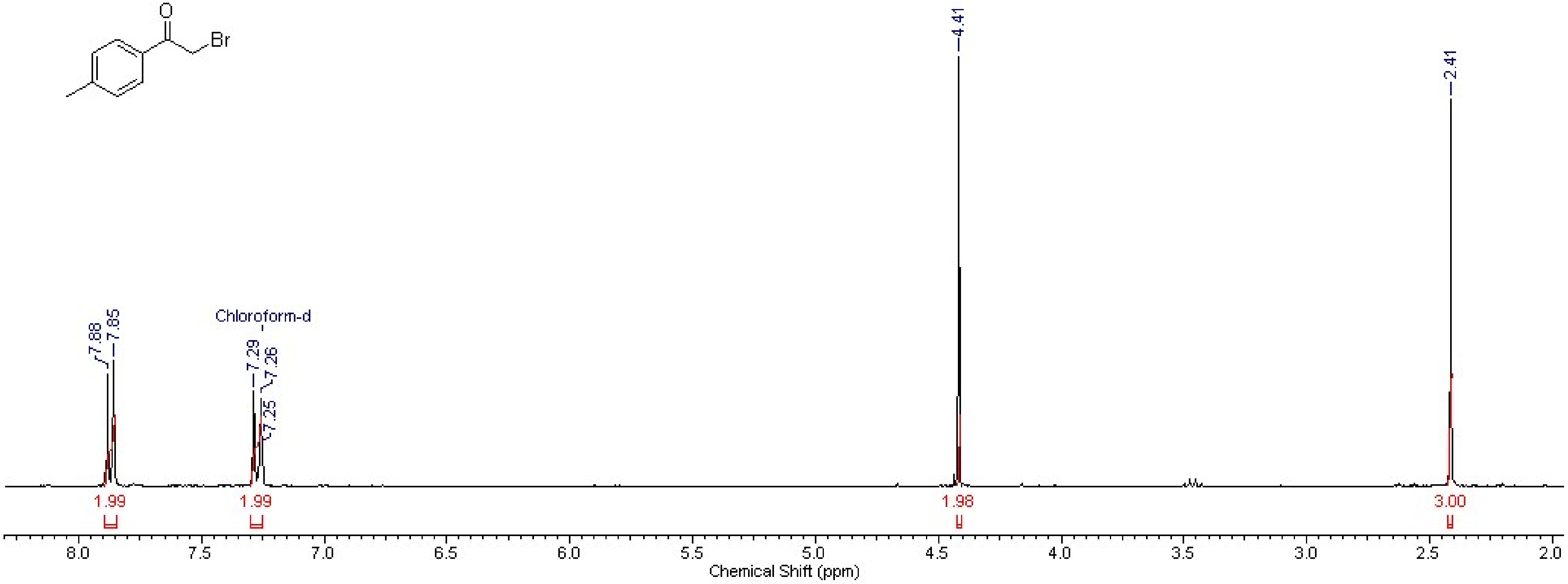

No.	(ppm)	(Hz)	Height
1	2.41	724.1	0.8993
2	4.41	1324.9	1.0000
3	7.25	2176.0	0.0813
4	7.26	2178.9	0.2045
5	7.29	2187.0	0.2205
6	7.85	2357.5	0.2922
7	7.88	2365.6	0.2618

No.	Annotation	(ppm)
1	Chloroform-d	7.25

^{13}C NMR of 2-bromo-1-(p-tolyl)ethan-1-one, **5d**

Acquisition Time (sec)	0.9050

Frequency (MHz)	75.48	Nucleus	13C	Number of Transients	64	Original Points Count	16316
Points Count	16384	Pulse Sequence	zgpg30	Solvent	CHLOROFORM-D	Sweep Width (Hz)	18028.85
Temperature (degree C)	27.108						

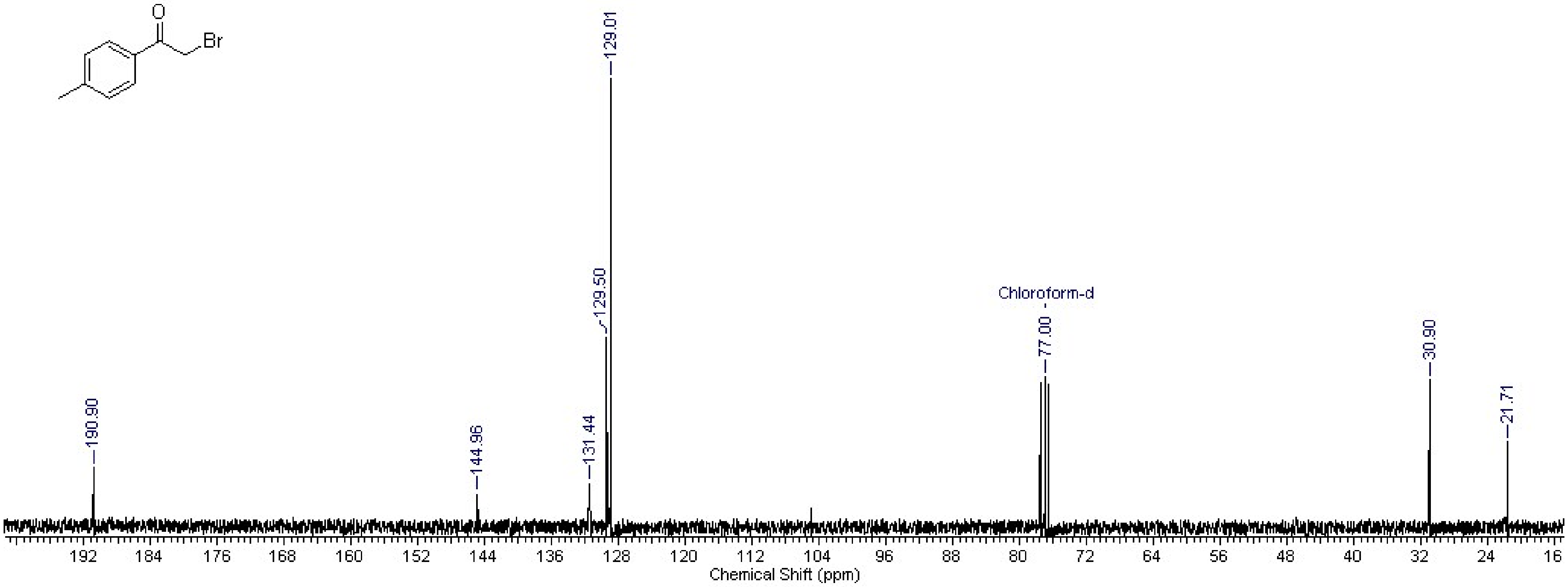

No.	(ppm)	(Hz)	Height
1	21.71	1638.7	0.1868
2	30.90	2332.0	0.3271
3	77.00	5811.6	0.3332
4	129.01	9737.0	1.0000
5	129.50	9774.4	0.4213
6	131.44	9920.7	0.0954
7	144.96	10940.9	0.0712
8	190.90	14408.4	0.1299

No.	Annotation	(ppm)
1	Chloroform-d	77.00

^{1}H NMR of 2-bromo-1-(2-chlorophenyl)ethan-1-one, **5e**

Acquisition Time (sec)	2.7150

Frequency (MHz)	300.13	Nucleus	1H	Number of Transients	1	Original Points Count	16316
Points Count	16384	Pulse Sequence	zg	Solvent	CHLOROFORM-D	Sweep Width (Hz)	6009.62
Temperature (degree C)	26.992						

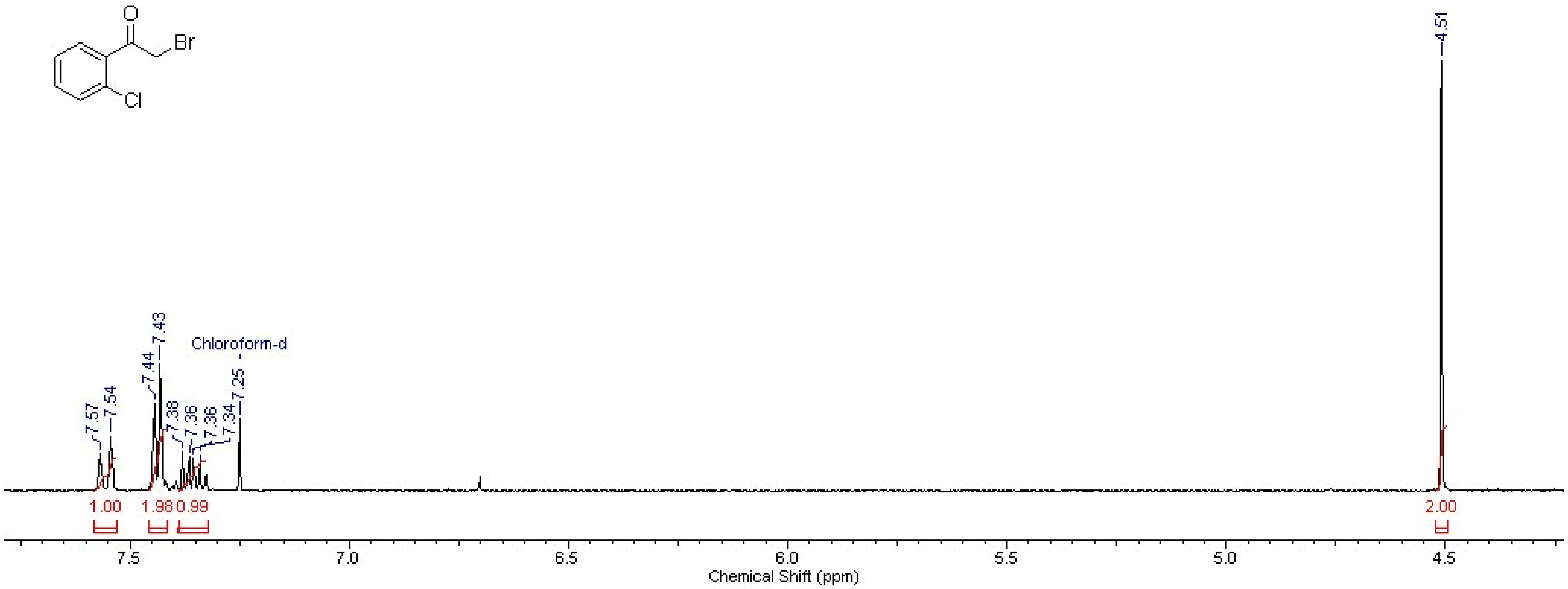

No.	(ppm)	(Hz)	Height
1	4.51	1353.2	1.0000
2	7.25	2176.0	0.1666
3	7.34	2203.1	0.0825
4	7.36	2207.5	0.0751
5	7.36	2210.4	0.0792
6	7.38	2214.8	0.0897
7	7.43	2230.6	0.2949
8	7.44	2233.9	0.2038
9	7.54	2264.0	0.1263
10	7.57	2271.7	0.0870

No.	Annotation	(ppm)
1	Chloroform-d	7.25

^{13}C NMR of 2-bromo-1-(2-chlorophenyl)ethan-1-one, **5e**

Acquisition Time (sec)	0.9050

Frequency (MHz)	75.48	Nucleus	13C	Number of Transients	256	Original Points Count	16316
Points Count	16384	Pulse Sequence	zgpg30	Solvent	CHLOROFORM-D	Sweep Width (Hz)	18028.85
Temperature (degree C)	27.021						

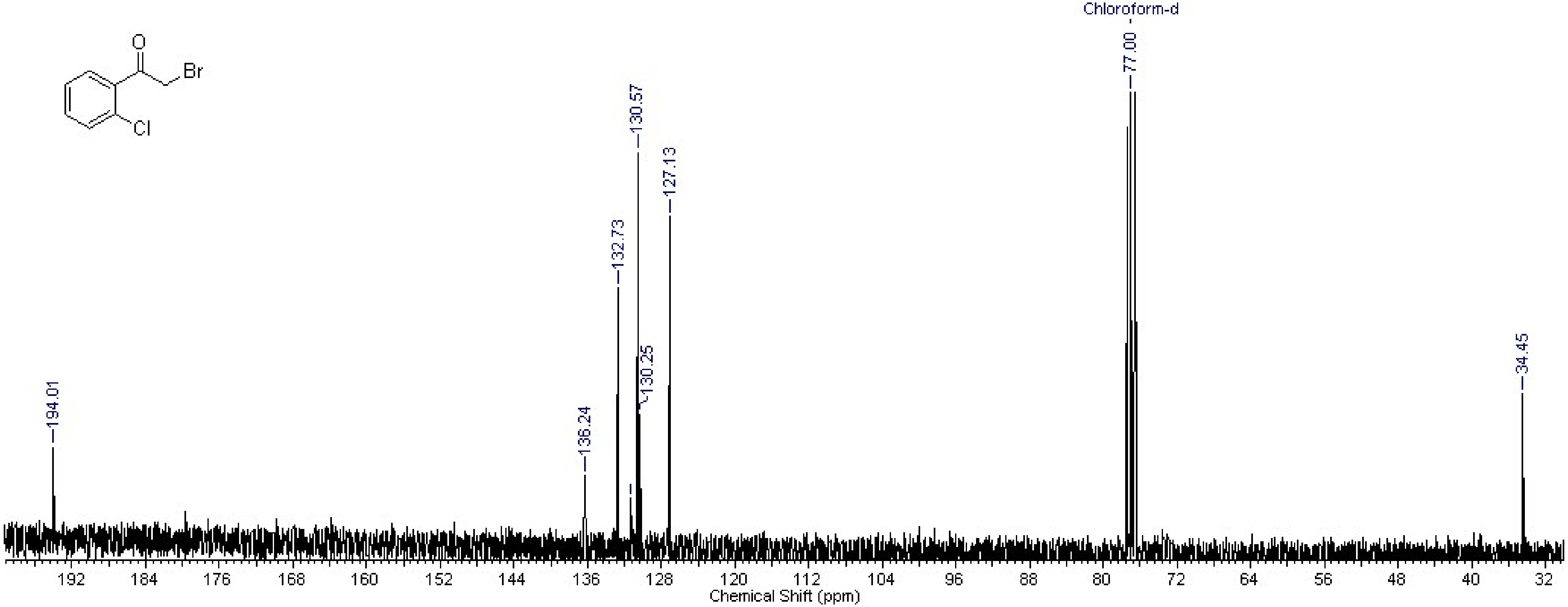

No.	(ppm)	(Hz)	Height
1	34.45	2600.5	0.3166
2	77.00	5811.6	0.9926
3	127.13	9595.0	0.6861
4	130.25	9830.5	0.2743
5	130.57	9854.7	0.8203
6	131.30	9909.7	0.0997
7	132.73	10017.6	0.5367
8	136.24	10282.8	0.1471
9	194.01	14642.8	0.2043

No.	Annotation	(ppm)
1	Chloroform-d	77.00

^{1}H NMR of 2,4-dibromopentan-3-one, **3a** (mixture of *meso*- and *rac*-isomers)

Acquisition Time (sec)	1.3518

Frequency (MHz)	300.13	Nucleus	1H	Number of Transients	1	Original Points Count	8124
Points Count	8192	Pulse Sequence	zg	Solvent	CHLOROFORM-D	Sweep Width (Hz)	6009.62
Temperature (degree C)	24.700						

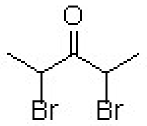

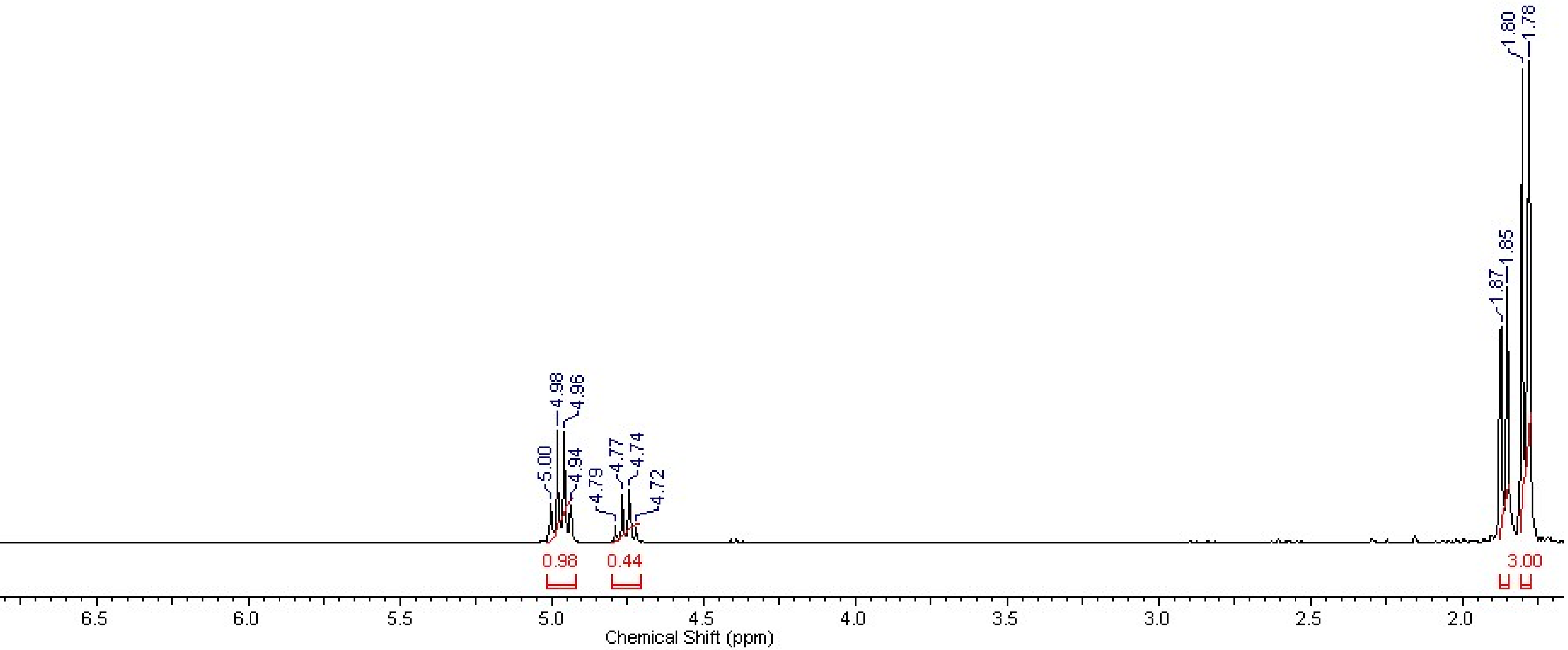

No.	(ppm)	(Hz)	Height
1	1.78	534.0	1.0000
2	1.80	541.3	0.9853
3	1.85	555.2	0.5319
4	1.87	561.9	0.4495
5	4.72	1417.3	0.0344
6	4.74	1423.9	0.1109

No.	(ppm)	(Hz)	Height
7	4.77	1430.5	0.1012
8	4.79	1437.9	0.0354
9	4.94	1481.9	0.0796
10	4.96	1488.5	0.2314
11	4.98	1495.1	0.2340
12	5.00	1501.7	0.0823

No.	Annotation	(ppm)
1	Chloroform-d	7.25

No.	(ppm)	Value	Absolute Value
1	[1.77 .. 1.81]	3.000	6.74624e+8
2	[1.85 .. 1.88]	1.380	3.10290e+8
3	[4.71 .. 4.80]	0.439	9.87598e+7
4	[4.92 .. 5.02]	0.977	2.19753e+8

^{13}C NMR of 2,4-dibromopentan-3-one, **3a** (mixture of *meso*- and *rac*-isomers)

**Acquisition Time (sec)**	0.9006

**Frequency (MHz)**	75.48	**Nucleus**	13C	**Number of Transients**	237	**Original Points Count**	16316
**Points Count**	16384	**Pulse Sequence**	zgpg30	**Solvent**	CHLOROFORM-D	**Sweep Width (Hz)**	18115.94
**Temperature (degree C)**	25.200						

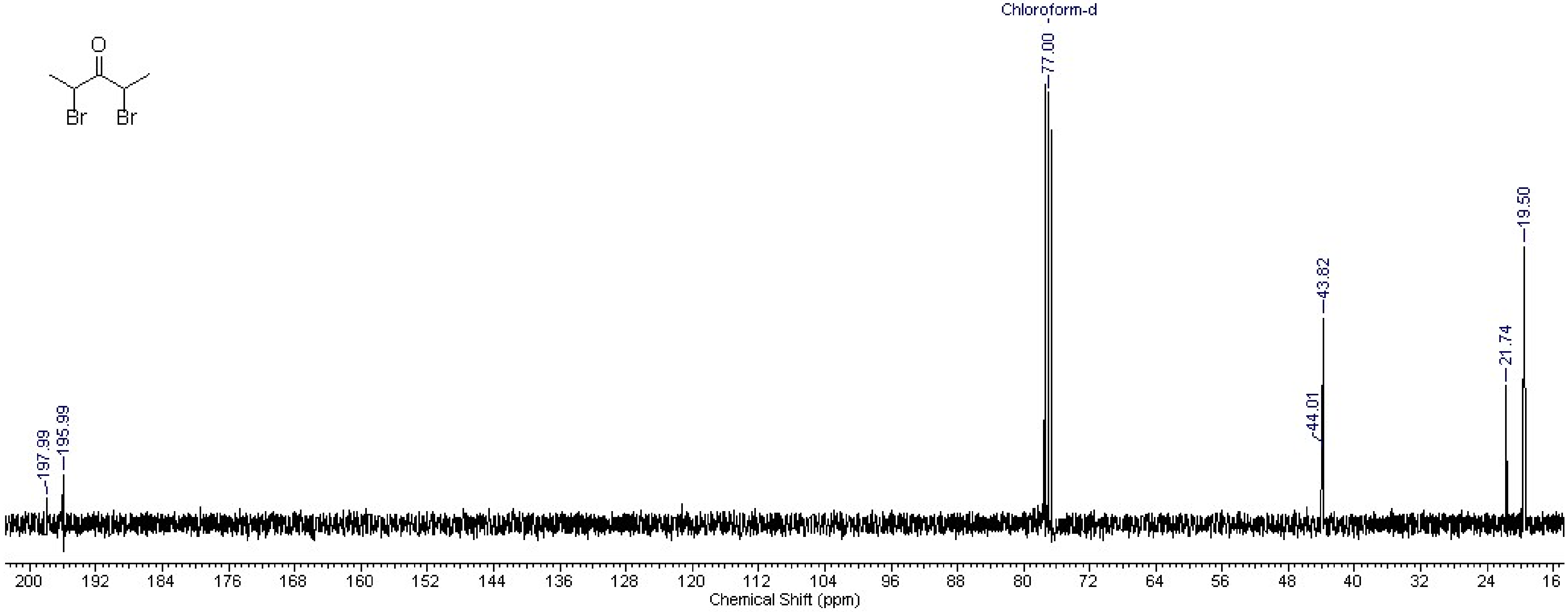

No.	(ppm)	(Hz)	Height
1	19.50	1471.5	0.6282
2	21.74	1640.7	0.3115
3	43.82	3307.1	0.4663
4	44.01	3321.4	0.1628
5	77.00	5811.7	0.9795
6	195.99	14792.8	0.1091
7	197.99	14943.2	0.0541

No.	Annotation	(ppm)
1	Chloroform-d	77.00

^{1}H NMR of 3,5-dibromoheptan-4-one, **3b**

Acquisition Time (sec)	1.3518

Frequency (MHz)	300.13	Nucleus	1H	Number of Transients	1	Original Points Count	8124
Points Count	8192	Pulse Sequence	zg	Solvent	CHLOROFORM-D	Sweep Width (Hz)	6009.62
Temperature (degree C)	30.800						

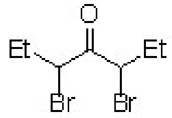

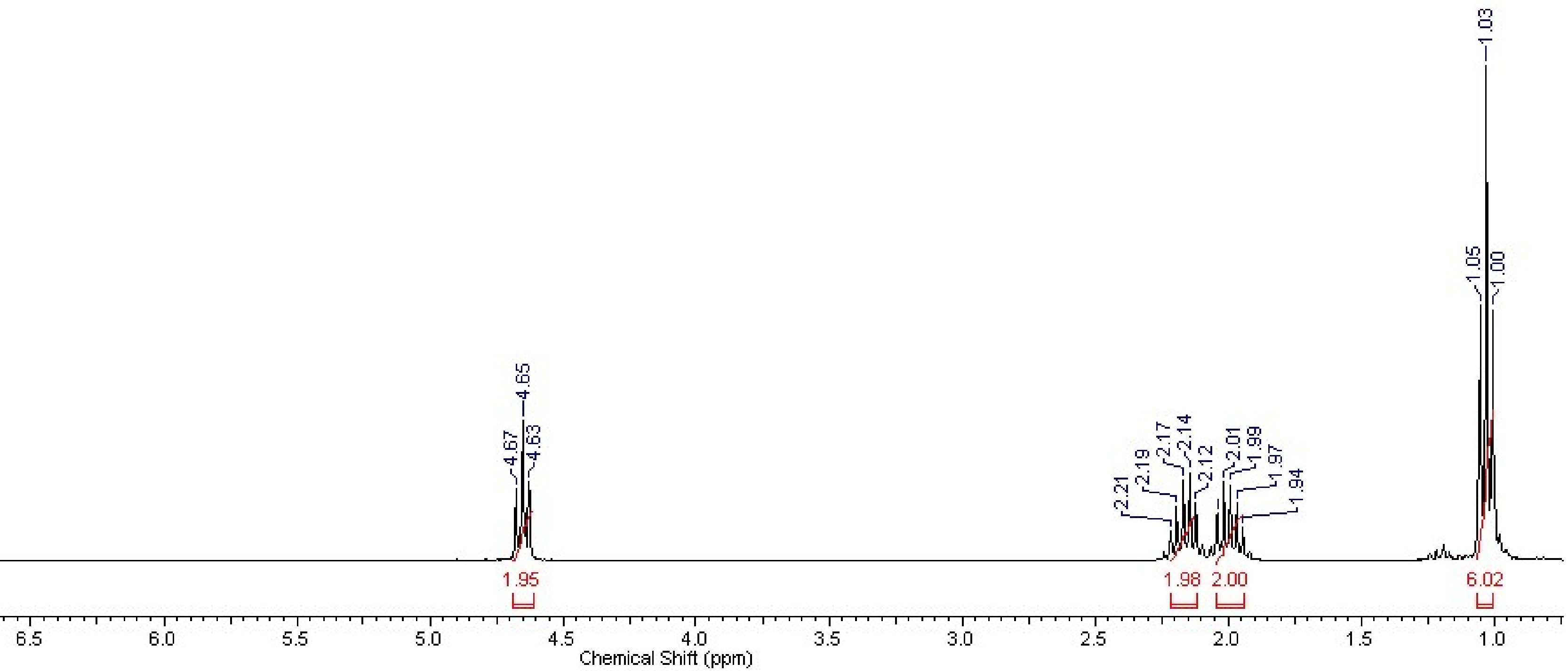

No.	(ppm)	(Hz)	Height
1	1.00	301.4	0.5081
2	1.03	308.7	1.0000
3	1.05	316.1	0.5175
4	1.94	583.1	0.0670
5	1.97	590.5	0.1185
6	1.99	597.8	0.1547
7	2.01	604.4	0.1589
8	2.04	611.7	0.0958
9	2.12	636.0	0.1158
10	2.14	643.3	0.1796
11	2.17	650.6	0.1647
12	2.19	658.0	0.1095
13	2.21	664.6	0.0590
14	4.63	1388.7	0.1587
15	4.65	1396.1	0.2817
16	4.67	1402.7	0.1453
17	7.25	2176.0	0.0591

No.	Annotation	(ppm)
1	Chloroform-d	7.25

No.	(ppm)	Value	Absolute Value
1	[1.00 .. 1.06]	6.020	5.82570e+8
2	[1.94 .. 2.04]	2.004	1.93887e+8
3	[2.12 .. 2.22]	1.981	1.91721e+8
4	[4.61 .. 4.69]	1.950	1.88728e+8

^{13}C NMR of 3,5-dibromoheptan-4-one, **3b**

Acquisition Time (sec)	0.9006

Frequency (MHz)	75.48	Nucleus	13C	Number of Transients	391	Original Points Count	16316
Points Count	16384	Pulse Sequence	zgpg30	Solvent	CHLOROFORM-D	Sweep Width (Hz)	18115.94
Temperature (degree C)	30.700						

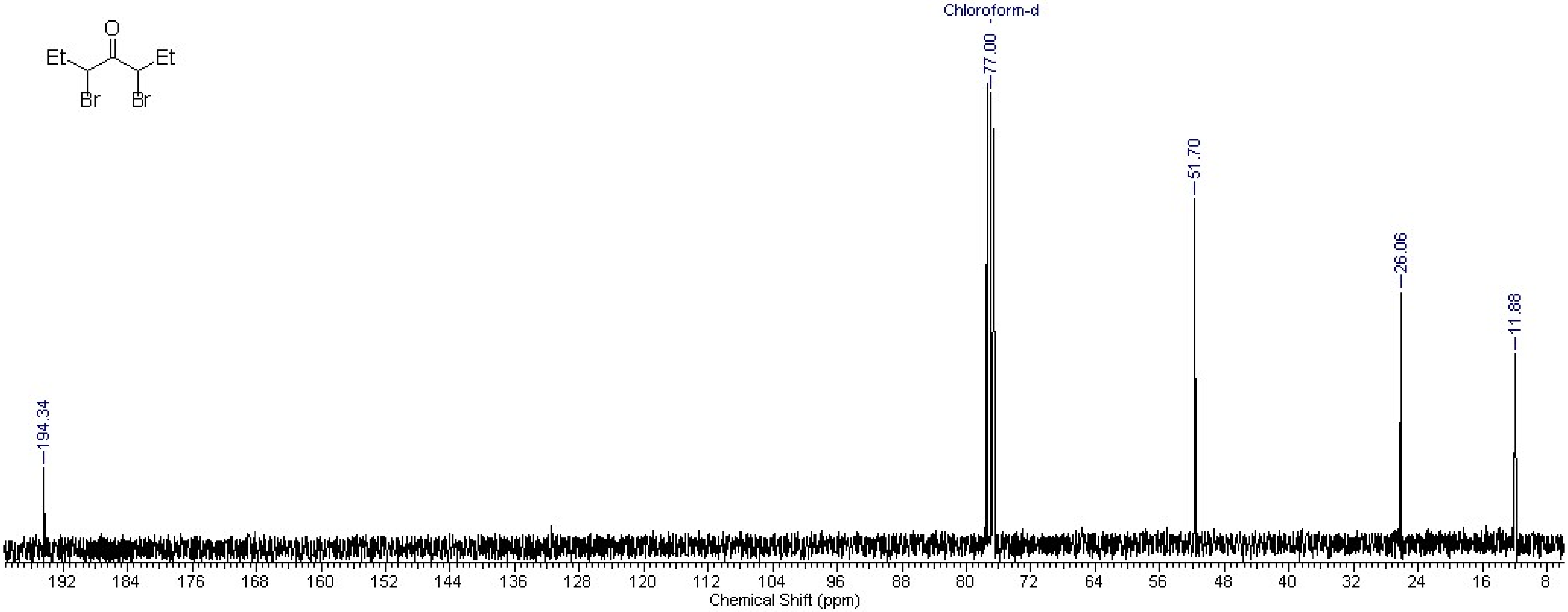

No.	(ppm)	(Hz)	Height
1	11.88	896.5	0.4164
2	26.06	1966.9	0.5491
3	51.70	3902.0	0.7500
4	77.00	5811.7	0.9776
5	194.34	14667.8	0.1700

No.	Annotation	(ppm)
1	Chloroform-d	77.00

^{1}H NMR of 4,6-dibromononane-5-one, **3c**

Acquisition Time (sec)	1.3518

Frequency (MHz)	300.13	*Nucleus*	1H	*Number of Transients*	1	*Original Points Count*	8124
Points Count	8192	*Pulse Sequence*	zg	*Solvent*	CHLOROFORM-D	*Sweep Width (Hz)*	6009.62
Temperature (degree C)	31.100						

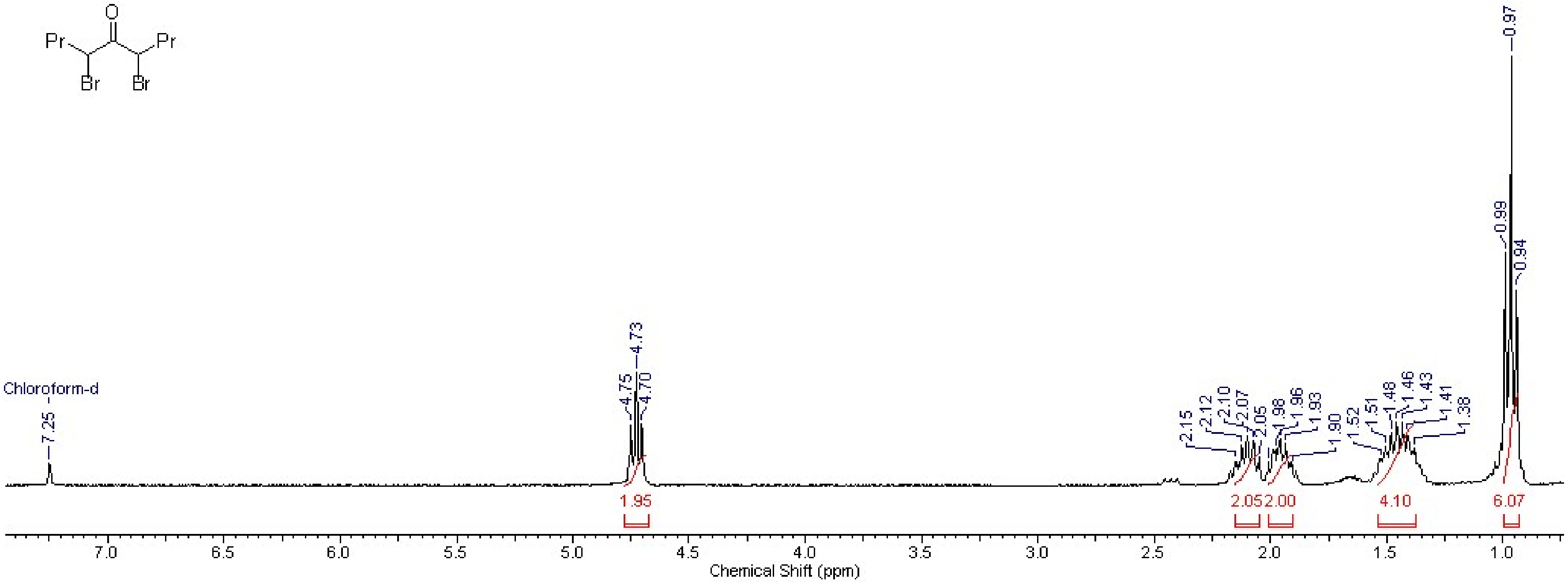

No.	(ppm)	(Hz)	Height	No.	(ppm)	(Hz)	Height
1	0.94	282.3	0.4554	13	1.96	587.5	0.1079
2	0.97	289.7	1.0000	14	1.98	593.4	0.0838
3	0.99	297.0	0.5437	15	2.01	602.9	0.0556
4	1.38	413.6	0.0883	16	2.05	614.7	0.0664
5	1.41	423.2	0.1216	17	2.07	622.0	0.1054
6	1.43	429.8	0.1465	18	2.10	630.1	0.1151
7	1.46	437.1	0.1472	19	2.12	637.4	0.0932
8	1.48	444.5	0.1242	20	2.15	645.5	0.0564
9	1.51	451.8	0.0918	21	4.70	1411.5	0.1442
10	1.52	457.7	0.0625	22	4.73	1418.8	0.2622
11	1.90	571.4	0.0480	23	4.75	1425.4	0.1412
12	1.93	580.2	0.0978	24	7.25	2176.0	0.0516

No.	Annotation	(ppm)
1	Chloroform-d	7.25

No.	(ppm)	Value	Absolute Value
1	[0.93 .. 1.00]	6.070	5.02642e+8
2	[1.38 .. 1.54]	4.097	3.39229e+8
3	[1.90 .. 2.01]	2.005	1.66016e+8
4	[2.05 .. 2.15]	2.048	1.69591e+8
5	[4.68 .. 4.78]	1.950	1.61436e+8

^{13}C NMR of 4,6-dibromononane-5-one, **3c**

Acquisition Time (sec)	0.9006						
Frequency (MHz)	75.48	***Nucleus***	13C	***Number of Transients***	100	***Original Points Count***	16316
Points Count	16384	***Pulse Sequence***	zgpg30	***Solvent***	CHLOROFORM-D	***Sweep Width (Hz)***	18115.94
Temperature (degree C)	33.200						

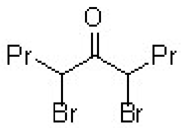

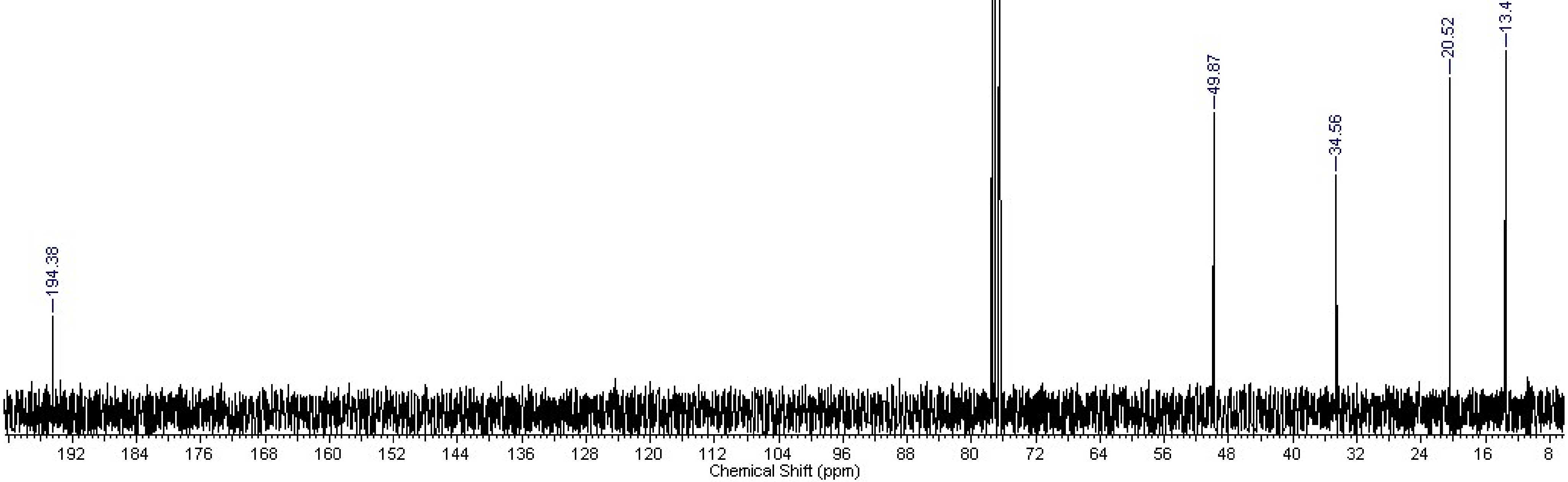

No.	(ppm)	(Hz)	Height
1	13.46	1015.9	0.6975
2	20.52	1548.9	0.6464
3	34.56	2608.2	0.4579
4	49.87	3763.8	0.5765
5	77.00	5811.7	1.0000
6	194.38	14671.1	0.1827

No.	Annotation	(ppm)
1	Chloroform-d	77.00

^{1}H NMR of 5,7-dibromoundecan-6-one, **3d**

Acquisition Time (sec)	1.3518

Frequency (MHz)	300.13	Nucleus	1H	Number of Transients	1	Original Points Count	8124
Points Count	8192	Pulse Sequence	zg	Solvent	CHLOROFORM-D	Sweep Width (Hz)	6009.62
Temperature (degree C)	28.800						

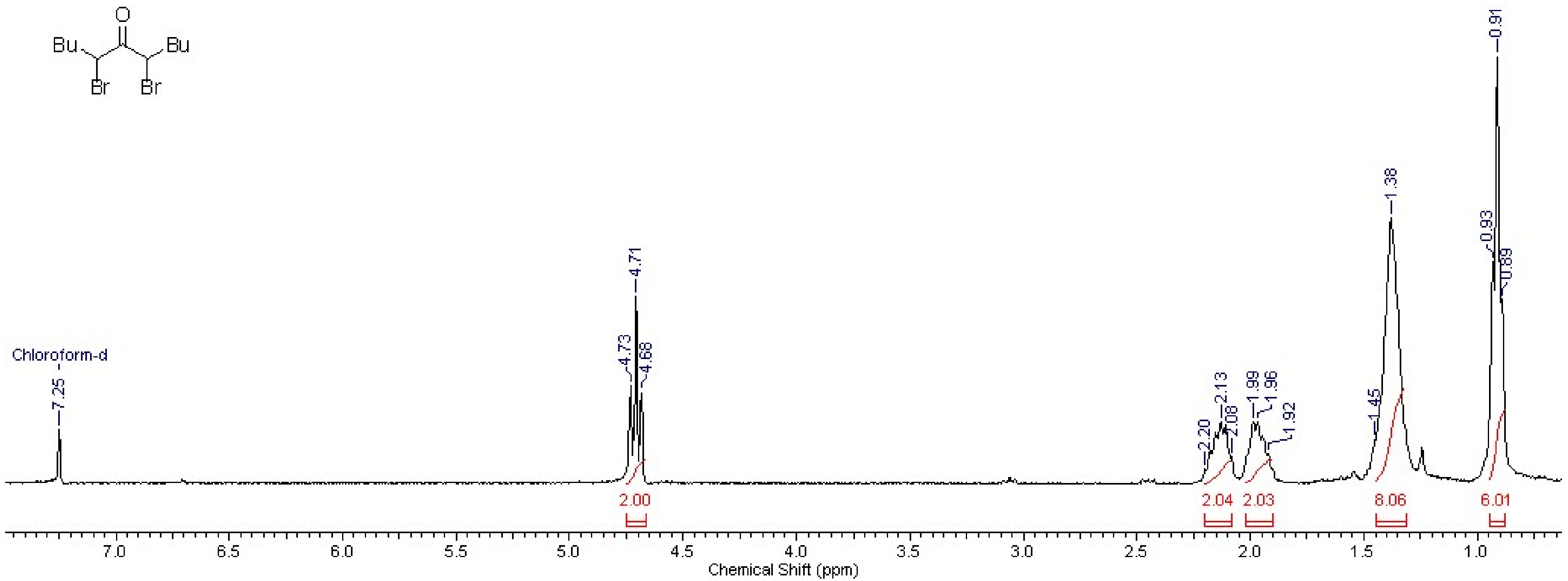

No.	(ppm)	(Hz)	Height
1	0.89	267.6	0.4320
2	0.91	274.2	1.0000
3	0.93	280.1	0.5202
4	1.38	413.6	0.6216
5	1.45	435.7	0.1049
6	1.92	576.5	0.0722
7	1.96	589.7	0.1459
8	1.99	596.3	0.1471

No.	(ppm)	(Hz)	Height
9	2.08	624.2	0.0651
10	2.13	638.9	0.1443
11	2.20	660.2	0.0342
12	4.68	1405.6	0.2143
13	4.71	1412.9	0.4392
14	4.73	1420.3	0.2315
15	7.25	2176.0	0.1282

No.	Annotation	(ppm)
1	Chloroform-d	7.25

No.	(ppm)	Value	Absolute Value
1	[0.88 .. 0.95]	6.011	3.96438e+8
2	[1.31 .. 1.44]	8.059	5.31490e+8
3	[1.90 .. 2.02]	2.032	1.33993e+8
4	[2.08 .. 2.20]	2.042	1.34679e+8
5	[4.66 .. 4.75]	2.000	1.31901e+8

^{13}C NMR of 5,7-dibromoundecan-6-one, **3d**

Acquisition Time (sec)	0.9006

Frequency (MHz)	75.48	Nucleus	13C	Number of Transients	179	Original Points Count	16316
Points Count	16384	Pulse Sequence	zgpg30	Solvent	CHLOROFORM-D	Sweep Width (Hz)	18115.94
Temperature (degree C)	29.200						

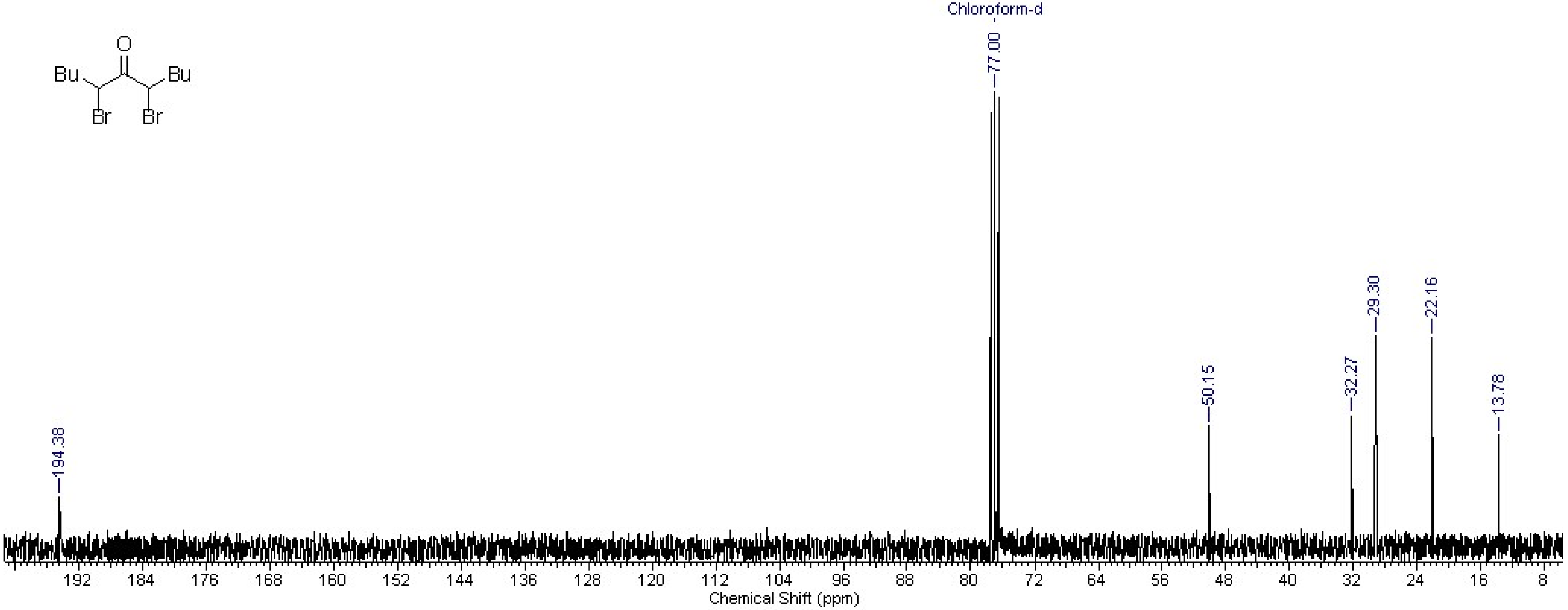

No.	(ppm)	(Hz)	Height
1	13.78	1040.2	0.2439
2	22.16	1672.7	0.4596
3	29.30	2211.2	0.4629
4	32.27	2435.7	0.2856
5	50.15	3784.8	0.2648
6	77.00	5811.7	1.0000
7	194.38	14671.1	0.1076

No.	Annotation	(ppm)
1	Chloroform-d	77.00

^{1}H NMR of 8,10-dibromoheptadecan-9-one, **3e**

Acquisition Time (sec)	1.3518

Frequency (MHz)	300.13	Nucleus	1H	Number of Transients	1	Original Points Count	8124
Points Count	8192	Pulse Sequence	zg	Solvent	CHLOROFORM-D	Sweep Width (Hz)	6009.62
Temperature (degree C)	27.100						

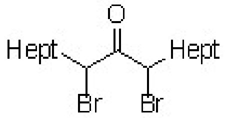

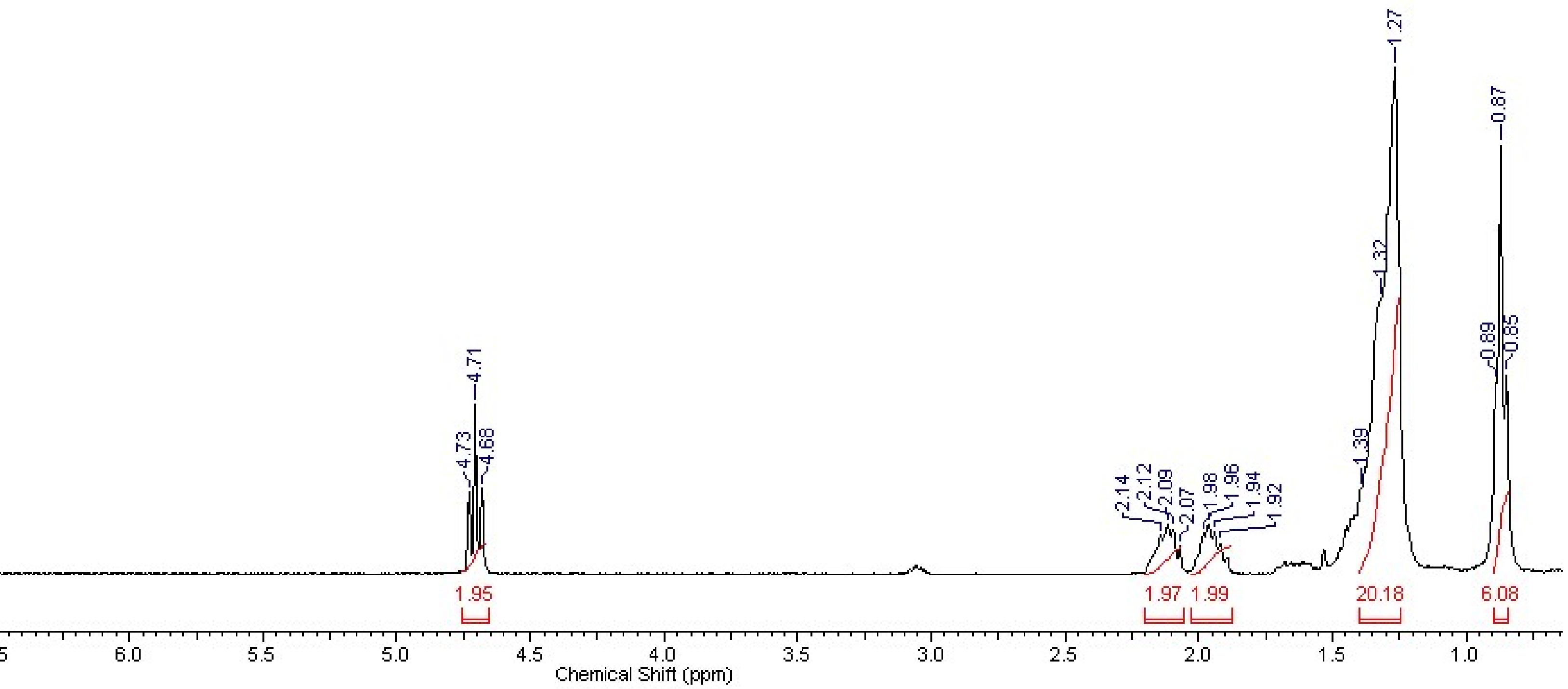

No.	(ppm)	(Hz)	Height
1	0.85	254.4	0.3944
2	0.87	261.8	0.8475
3	0.89	266.9	0.3804
4	1.27	380.6	1.0000
5	1.32	396.8	0.5447
6	1.39	418.1	0.1701
7	1.92	575.8	0.0623
8	1.94	582.4	0.0850
9	1.96	589.0	0.0997

No.	(ppm)	(Hz)	Height
10	1.98	594.1	0.0816
11	2.07	621.3	0.0559
12	2.09	628.6	0.0907
13	2.12	635.2	0.0995
14	2.14	642.6	0.0791
15	4.68	1404.9	0.1704
16	4.71	1412.2	0.3348
17	4.73	1419.5	0.1626
18	7.25	2176.0	0.0986

No.	Annotation	(ppm)
1	Chloroform-d	7.25

No.	(ppm)	Value	Absolute Value
1	[0.84 .. 0.90]	6.080	2.36951e+8
2	[1.25 .. 1.40]	20.178	7.86371e+8
3	[1.87 .. 2.03]	1.986	7.73928e+7
4	[2.06 .. 2.20]	1.975	7.69506e+7
5	[4.66 .. 4.76]	1.955	7.61783e+7

^{13}C NMR of 8,10-dibromoheptadecan-9-one, **3e**

Acquisition Time (sec)	0.9006

Frequency (MHz)	75.48	Nucleus	13C	Number of Transients	201	Original Points Count	16316
Points Count	16384	Pulse Sequence	zgpg30	Solvent	CHLOROFORM-D	Sweep Width (Hz)	18115.94
Temperature (degree C)	26.900						

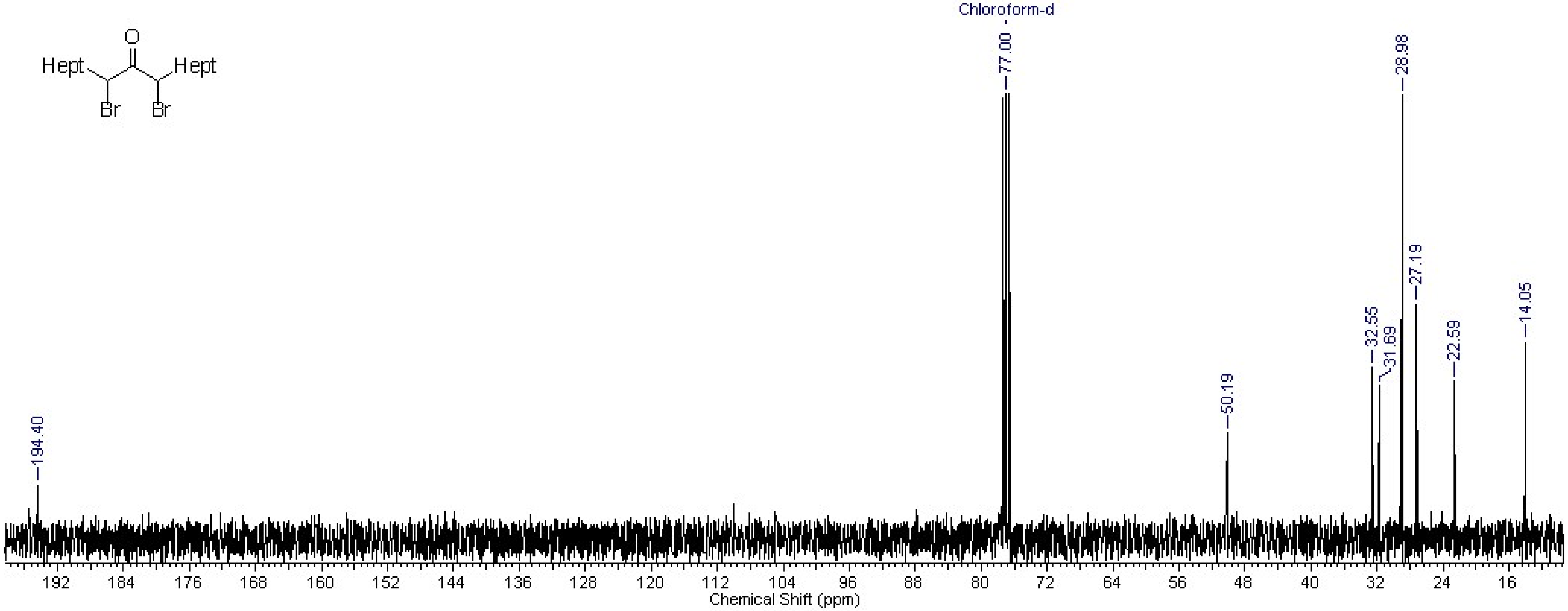

No.	(ppm)	(Hz)	Height
1	14.05	1060.1	0.4222
2	22.59	1704.8	0.3399
3	27.19	2052.0	0.5046
4	28.98	2186.9	0.9554
5	31.69	2391.5	0.3293
6	32.55	2456.7	0.3690
7	50.19	3788.1	0.2269
8	77.00	5811.7	0.9745
9	194.40	14672.2	0.1154

No.	Annotation	(ppm)
1	Chloroform-d	77.00

^{1}H NMR of 3,5-dibromononan-4-one, **3f**

Acquisition Time (sec)	1.3518						
Frequency (MHz)	300.13	Nucleus	1H	Number of Transients	1	Original Points Count	8124
Points Count	8192	Pulse Sequence	zg	Solvent	CHLOROFORM-D	Sweep Width (Hz)	6009.62
Temperature (degree C)	23.600						

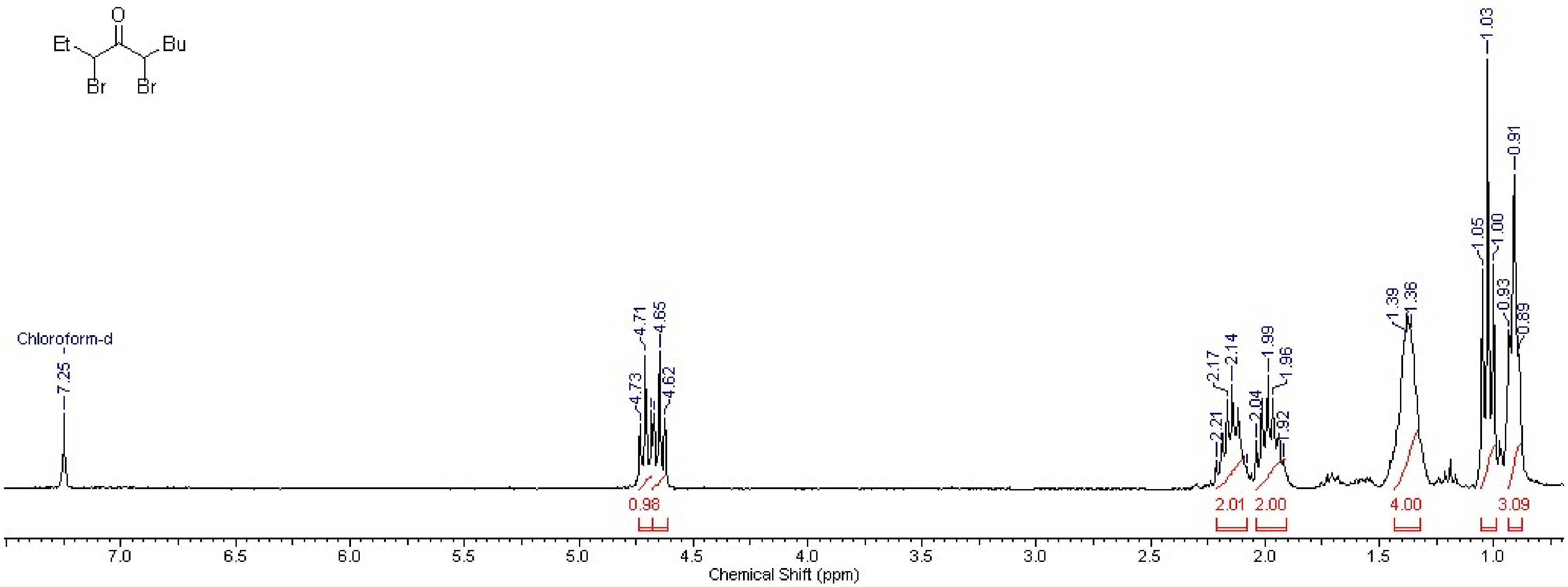

No.	(ppm)	(Hz)	Height
1	0.89	266.9	0.3145
2	0.91	273.5	0.7324
3	0.93	280.1	0.3706
4	1.00	300.7	0.5232
5	1.03	308.0	1.0000
6	1.05	315.3	0.5117
7	1.36	408.5	0.3644
8	1.39	416.6	0.3487
9	1.92	575.1	0.0656
10	1.96	589.0	0.2118
11	1.99	596.3	0.2628
12	2.04	611.0	0.1127

No.	(ppm)	(Hz)	Height
13	2.08	624.2	0.0517
14	2.14	642.6	0.2438
15	2.17	649.9	0.2062
16	2.21	663.8	0.0669
17	4.62	1387.2	0.1660
18	4.65	1394.6	0.3195
19	4.67	1401.9	0.1754
20	4.68	1405.6	0.1818
21	4.71	1412.9	0.3111
22	4.73	1420.3	0.1527
23	7.25	2176.0	0.1758

No.	Annotation	(ppm)
1	Chloroform-d	7.25

No.	(ppm)	Value	Absolute Value
1	[0.88 .. 0.94]	3.088	1.61246e+8
2	[0.99 .. 1.06]	3.010	1.57189e+8
3	[1.32 .. 1.43]	4.002	2.09014e+8
4	[1.91 .. 2.04]	2.004	1.04656e+8
5	[2.08 .. 2.21]	2.010	1.04975e+8
6	[4.61 .. 4.68]	0.972	5.07435e+7
7	[4.68 .. 4.74]	0.984	5.13641e+7

^{13}C NMR of 3,5-dibromononan-4-one, **3f**

**Acquisition Time (sec)**	0.9006

**Frequency (MHz)**	75.48	**Nucleus**	13C	**Number of Transients**	133	**Original Points Count**	16316
**Points Count**	16384	**Pulse Sequence**	zgpg30	**Solvent**	CHLOROFORM-D	**Sweep Width (Hz)**	18115.94
**Temperature (degree C)**	25.600						

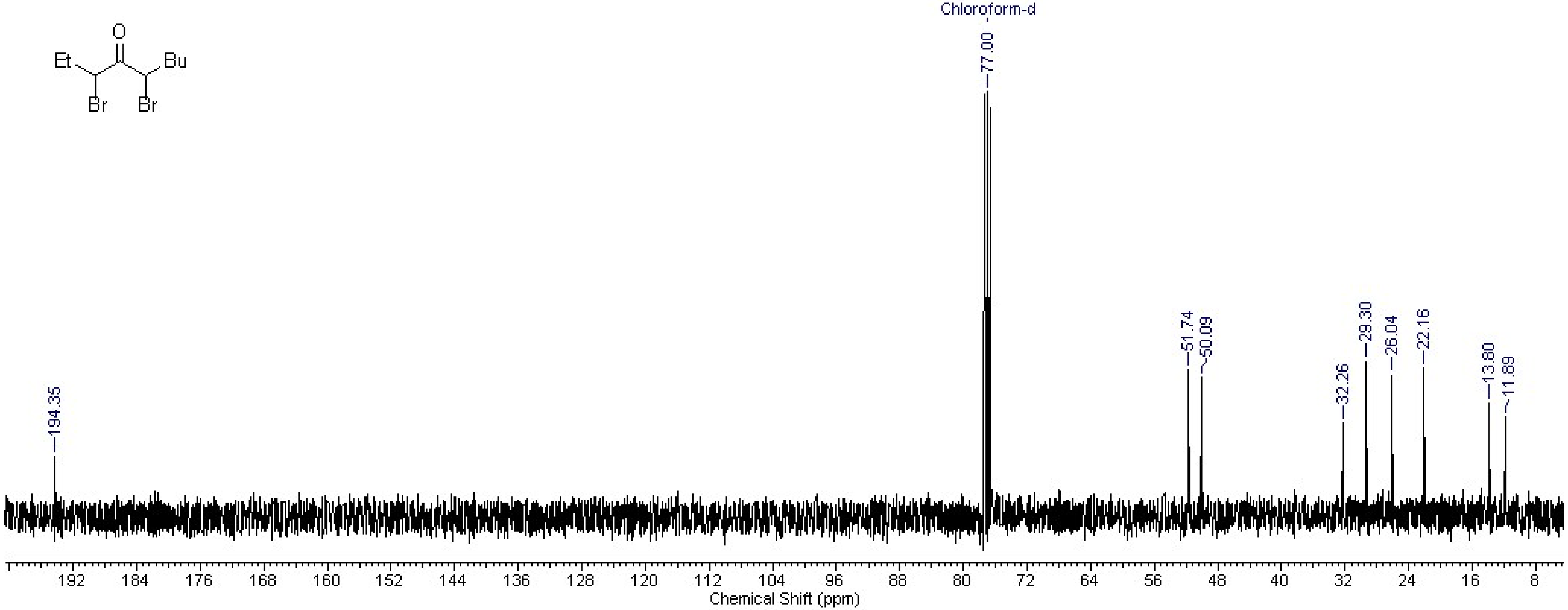

No.	(ppm)	(Hz)	Height
1	11.89	897.6	0.2315
2	13.80	1041.3	0.2639
3	22.16	1672.7	0.3443
4	26.04	1965.8	0.3299
5	29.30	2211.2	0.3622
6	32.26	2434.6	0.2162
7	50.09	3780.3	0.3254
8	51.74	3905.3	0.3417
9	77.00	5811.7	1.0000
10	194.35	14668.9	0.1387

No.	Annotation	(ppm)
1	Chloroform-d	77.00

^{1}H NMR of 2,4-dibromo-2-methyloctan-3-one, **3g**

Acquisition Time (sec)	1.3518

Frequency (MHz)	300.13	Nucleus	1H	Number of Transients	1	Original Points Count	8124
Points Count	8192	Pulse Sequence	zg	Solvent	CHLOROFORM-D	Sweep Width (Hz)	6009.62
Temperature (degree C)	26.800						

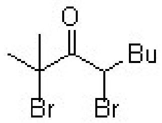

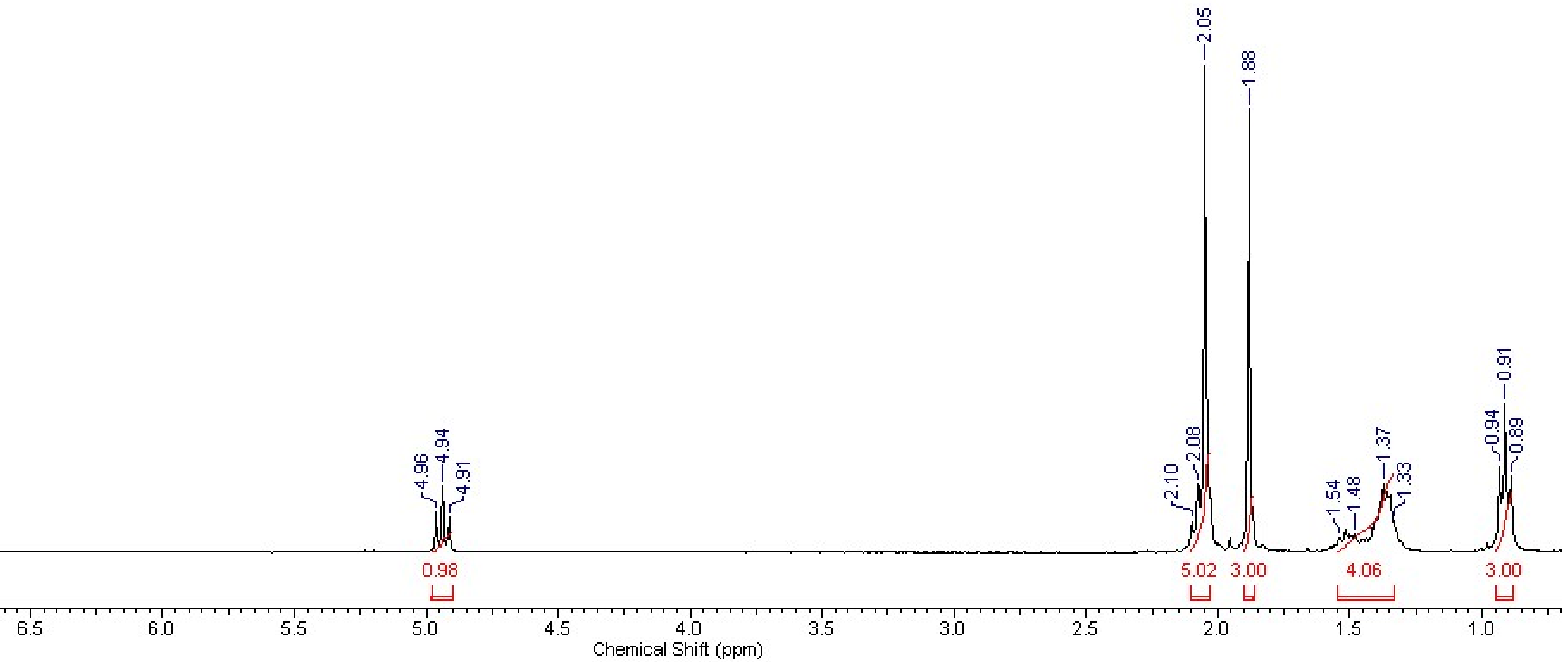

No.	(ppm)	(Hz)	Height
1	0.89	266.9	0.1568
2	0.91	274.2	0.3052
3	0.94	280.9	0.1766
4	1.33	399.7	0.0658
5	1.37	412.2	0.1413
6	1.48	445.2	0.0385
7	1.54	462.1	0.0295

No.	(ppm)	(Hz)	Height
8	1.88	564.8	0.9104
9	2.05	614.7	1.0000
10	2.08	623.5	0.1384
11	2.10	630.1	0.0628
12	4.91	1474.6	0.0726
13	4.94	1481.9	0.1370
14	4.96	1489.2	0.0821

No.	Annotation	(ppm)
1	Chloroform-d	7.25

No.	(ppm)	Value	Absolute Value
1	[0.88 .. 0.95]	3.000	3.15938e+8
2	[1.33 .. 1.55]	4.059	4.27429e+8
3	[1.86 .. 1.90]	2.999	3.15848e+8
4	[2.03 .. 2.10]	5.025	5.29145e+8
5	[4.90 .. 4.98]	0.981	1.03359e+8

^{13}C NMR of 2,4-dibromo-2-methyloctan-3-one, **3g**

Acquisition Time (sec)	0.9006

Frequency (MHz)	75.48	Nucleus	13C	Number of Transients	112	Original Points Count	16316
Points Count	16384	Pulse Sequence	zgpg30	Solvent	CHLOROFORM-D	Sweep Width (Hz)	18115.94
Temperature (degree C)	26.900						

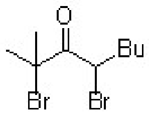

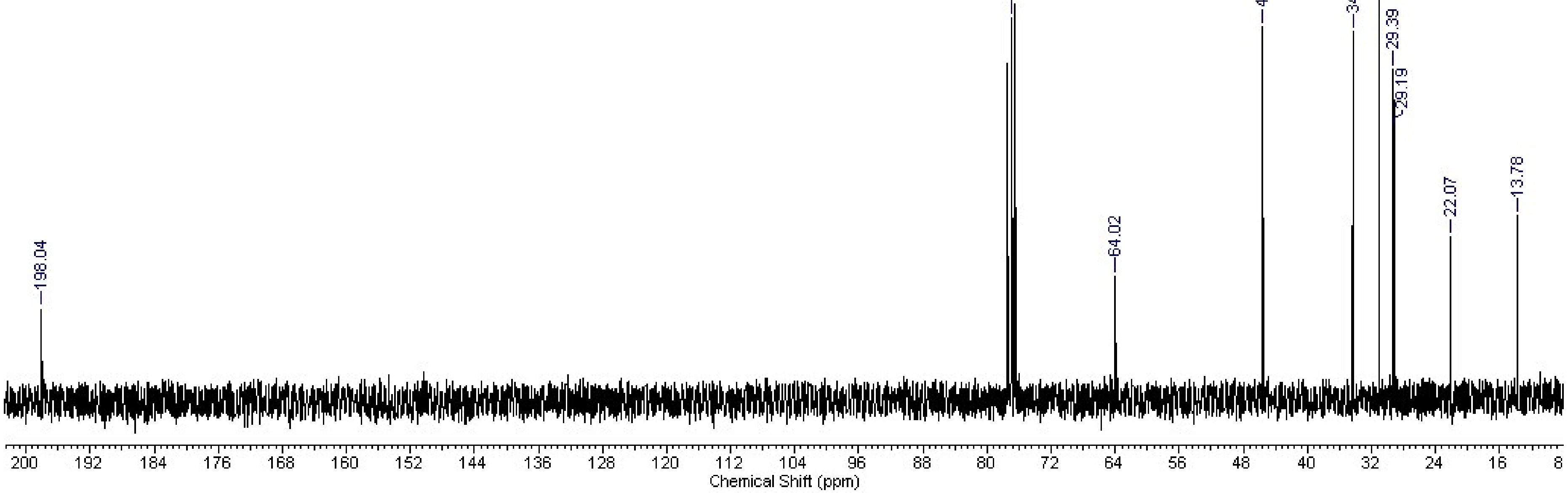

No.	(ppm)	(Hz)	Height
1	13.78	1040.2	0.4335
2	22.07	1666.1	0.3843
3	29.19	2203.5	0.6396
4	29.39	2217.9	0.7771
5	31.00	2339.5	1.0000
6	34.32	2590.5	0.8641
7	45.65	3445.3	0.8754
8	64.02	4831.9	0.2937
9	77.00	5811.7	0.8980
10	198.04	14947.6	0.2161

No.	Annotation	(ppm)
1	Chloroform-d	77.00

^{1}H NMR of 1,1-dibromo-3,3-dimethylbutan-2-one, **6a**

Acquisition Time (sec)	1.3518

Frequency (MHz)	300.13	Nucleus	1H	Number of Transients	1	Original Points Count	8124
Points Count	8192	Pulse Sequence	zg	Solvent	CHLOROFORM-D	Sweep Width (Hz)	6009.62
Temperature (degree C)	27.400						

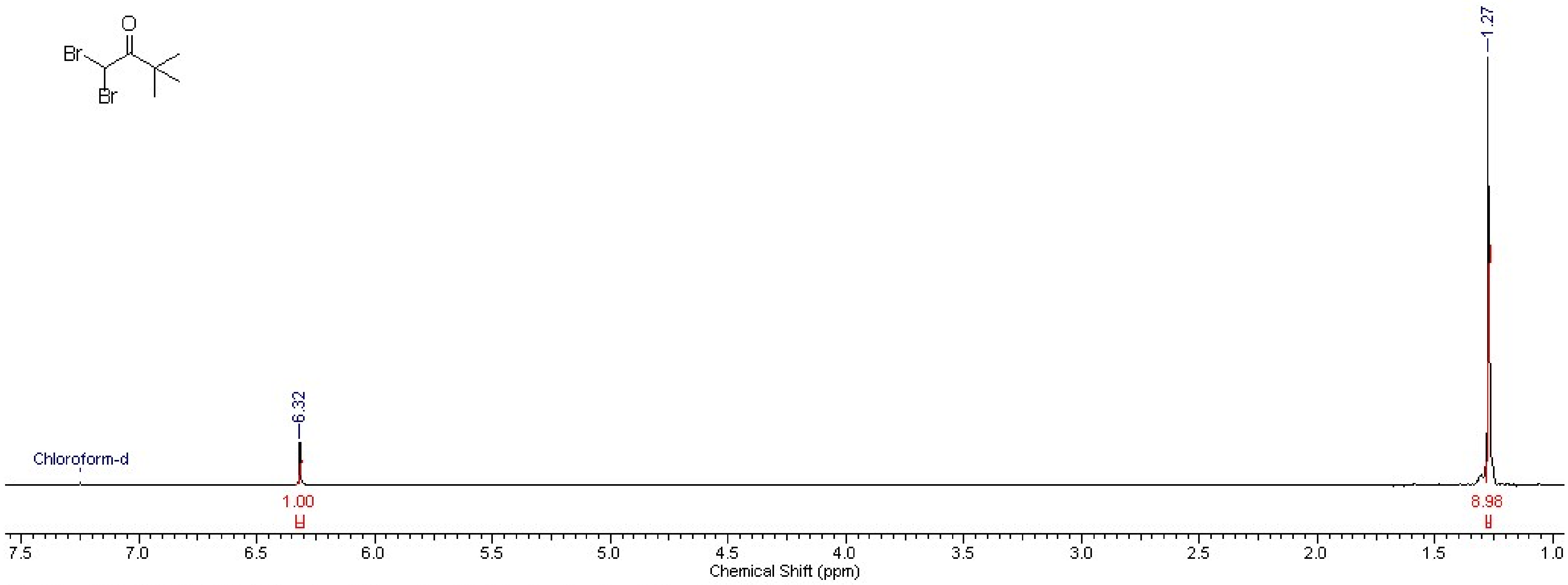

No.	(ppm)	(Hz)	Height
1	1.27	382.1	1.0000
2	6.32	1896.4	0.1008

No.	Annotation	(ppm)
1	Chloroform-d	7.25

No.	(ppm)	Value	Absolute Value
1	[1.26 .. 1.29]	8.985	1.15932e+9
2	[6.30 .. 6.33]	1.000	1.29031e+8

^{13}C NMR of 1,1-dibromo-3,3-dimethylbutan-2-one, **6a**

Acquisition Time (sec)	0.9006

Frequency (MHz)	75.48	Nucleus	13C	Number of Transients	117	Original Points Count	16316
Points Count	16384	Pulse Sequence	zgpg30	Solvent	CHLOROFORM-D	Sweep Width (Hz)	18115.94
Temperature (degree C)	27.400						

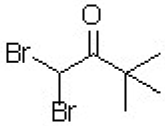

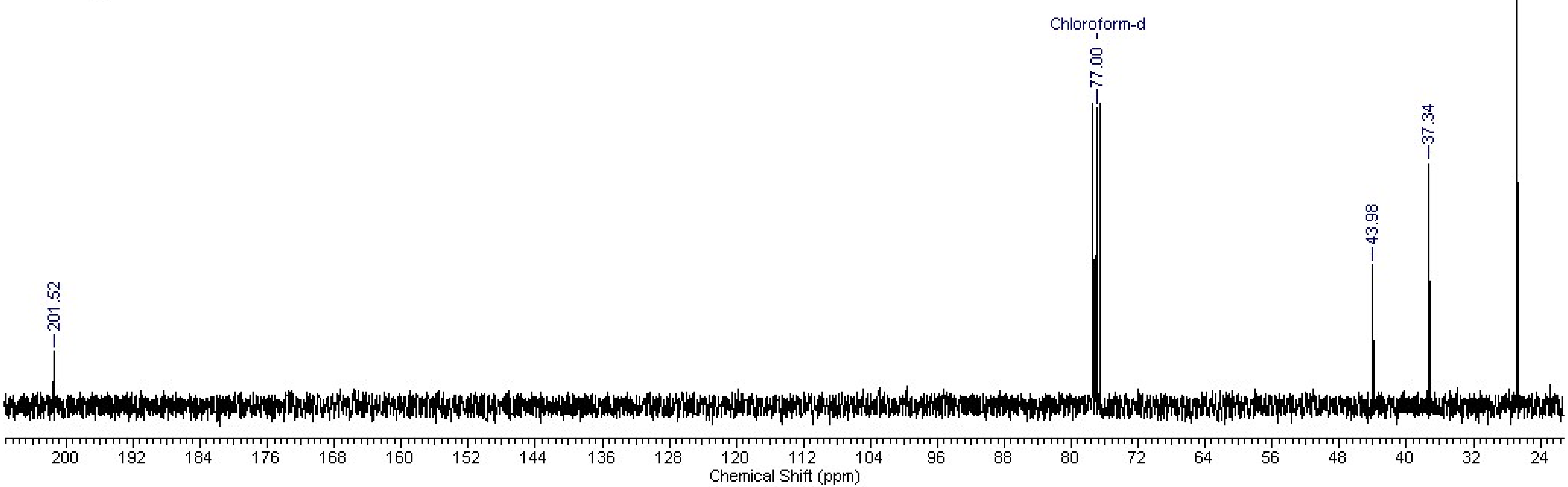

No.	(ppm)	(Hz)	Height
1	26.79	2022.2	1.0000
2	37.34	2818.3	0.5524
3	43.98	3319.2	0.3209
4	77.00	5811.7	0.6810
5	201.52	15209.7	0.1223

No.	Annotation	(ppm)
1	Chloroform-d	77.00

^{1}H NMR of 2,2-dibromo-1-phenylethanone, **6b**

Acquisition Time (sec)	1.3518

Frequency (MHz)	300.13	Nucleus	1H	Number of Transients	1	Original Points Count	8124
Points Count	8192	Pulse Sequence	zg	Solvent	CHLOROFORM-D	Sweep Width (Hz)	6009.62
Temperature (degree C)	24.700						

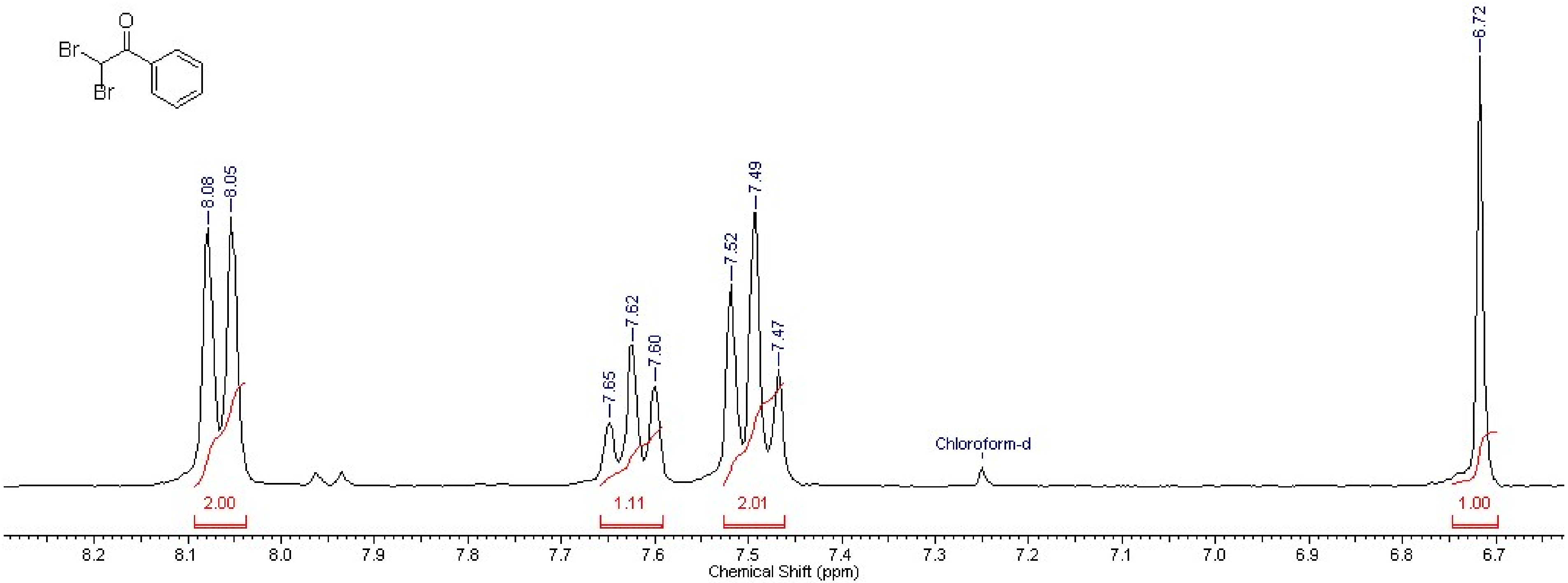

No.	(ppm)	(Hz)	Height
1	6.72	2016.0	1.0000
2	7.47	2241.3	0.2717
3	7.49	2248.6	0.6373
4	7.52	2256.7	0.4721
5	7.60	2280.9	0.2355
6	7.62	2288.2	0.3279
7	7.65	2295.6	0.1503
8	8.05	2417.3	0.6282
9	8.08	2424.7	0.6018

No.	Annotation	(ppm)
1	Chloroform-d	7.25

No.	(ppm)	Value	Absolute Value
1	[6.70 .. 6.75]	1.000	2.64646e+8
2	[7.46 .. 7.53]	2.014	5.32906e+8
3	[7.59 .. 7.66]	1.106	2.92664e+8
4	[8.04 .. 8.09]	1.999	5.29115e+8

^{13}C NMR of 2,2-dibromo-1-phenylethanone, **6b**

Acquisition Time (sec)	0.9006

Frequency (MHz)	75.48	Nucleus	13C	Number of Transients	147	Original Points Count	16316
Points Count	16384	Pulse Sequence	zgpg30	Solvent	CHLOROFORM-D	Sweep Width (Hz)	18115.94
Temperature (degree C)	24.800						

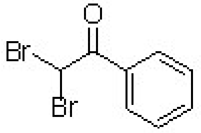

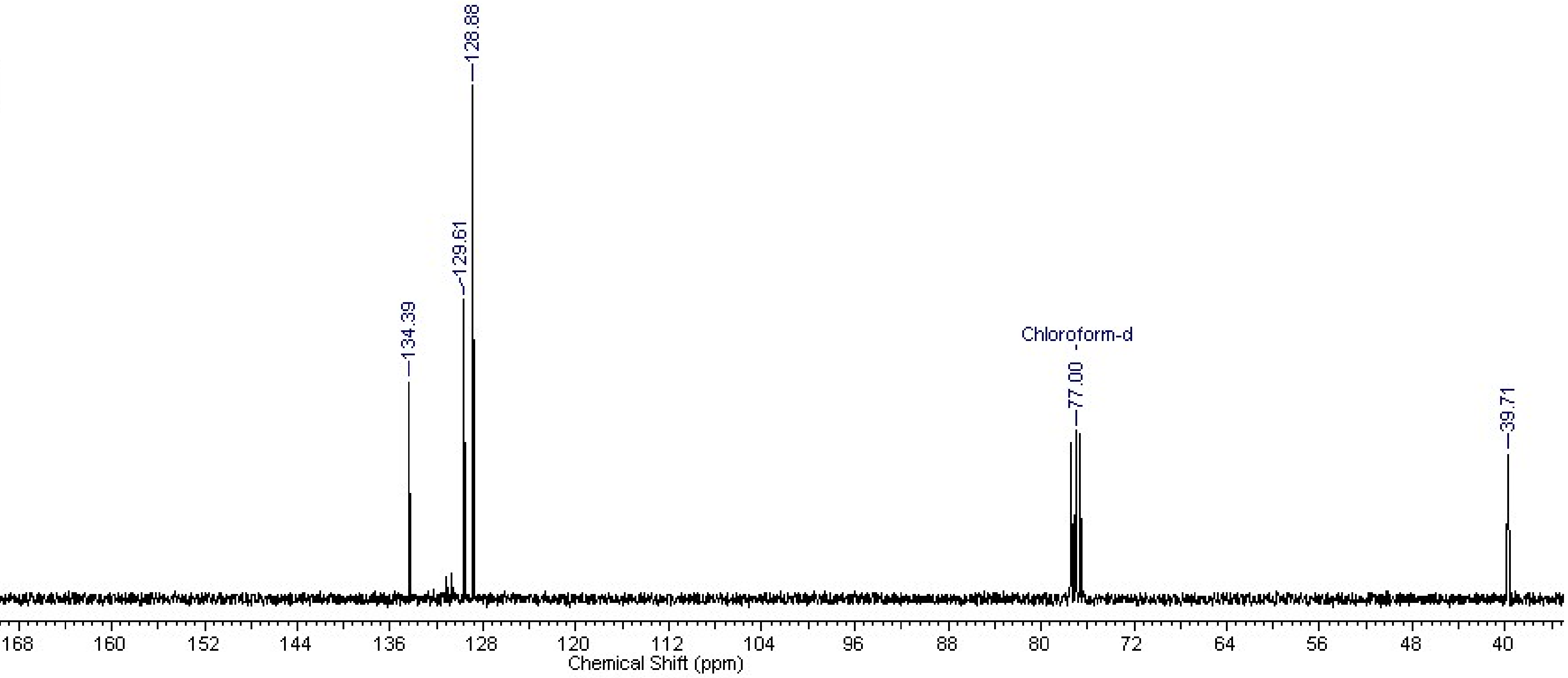

No.	(ppm)	(Hz)	Height
1	39.71	2997.5	0.2802
2	77.00	5811.7	0.3262
3	128.88	9727.2	1.0000
4	129.61	9782.5	0.5834
5	134.39	10143.0	0.4224
6	185.88	14029.8	0.0669

No.	Annotation	(ppm)
1	Chloroform-d	77.00

^{1}H NMR of 2,2-dibromo-1-(p-tolyl)ethan-1-one, **6d**

Acquisition Time (sec)	2.7150

Frequency (MHz)	300.13	Nucleus	1H	Number of Transients	1	Original Points Count	16316
Points Count	16384	Pulse Sequence	zg	Solvent	CHLOROFORM-D	Sweep Width (Hz)	6009.62
Temperature (degree C)	26.994						

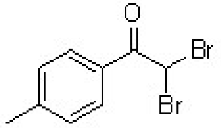

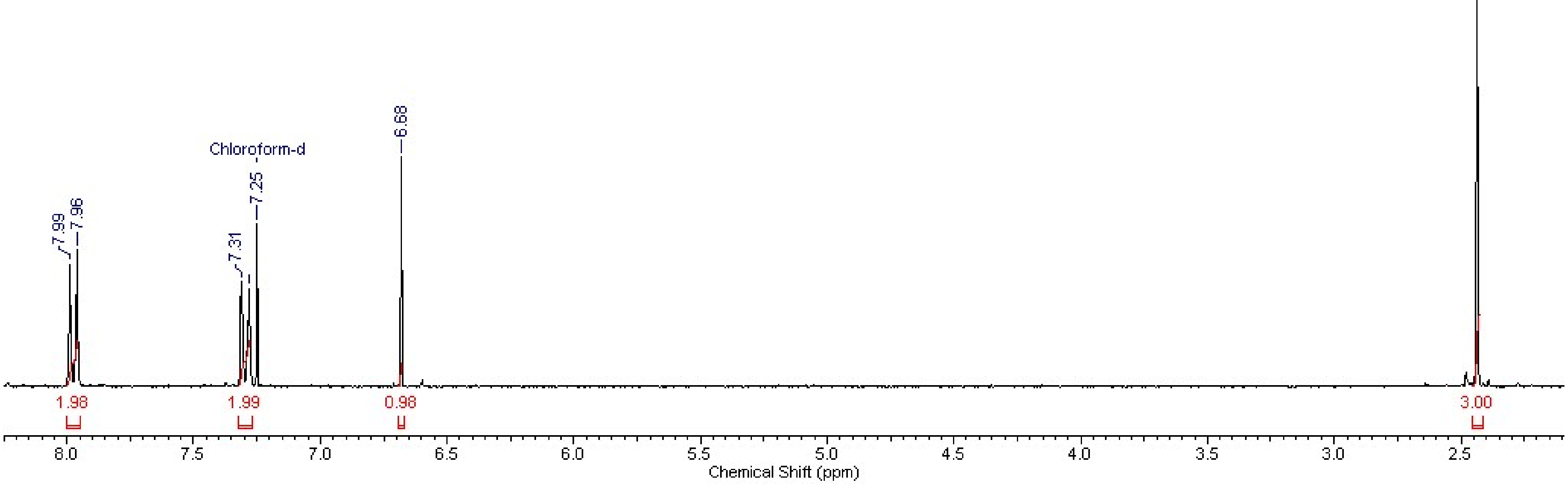

No.	(ppm)	(Hz)	Height
1	2.44	731.1	1.0000
2	6.68	2005.0	0.5349
3	7.25	2176.0	0.3806
4	7.28	2185.9	0.2286
5	7.31	2194.3	0.2445
6	7.96	2389.1	0.3200
7	7.99	2397.5	0.2861

No.	Annotation	(ppm)
1	Chloroform-d	7.25

^{13}C NMR of 2,2-dibromo-1-(p-tolyl)ethan-1-one, **6d**

Acquisition Time (sec)	0.9050

Frequency (MHz)	75.48	Nucleus	13C	Number of Transients	512
Original Points Count	16316	Points Count	16384	Pulse Sequence	zgpg30
Solvent	CHLOROFORM-D	Sweep Width (Hz)	18028.85	Temperature (degree C)	27.009

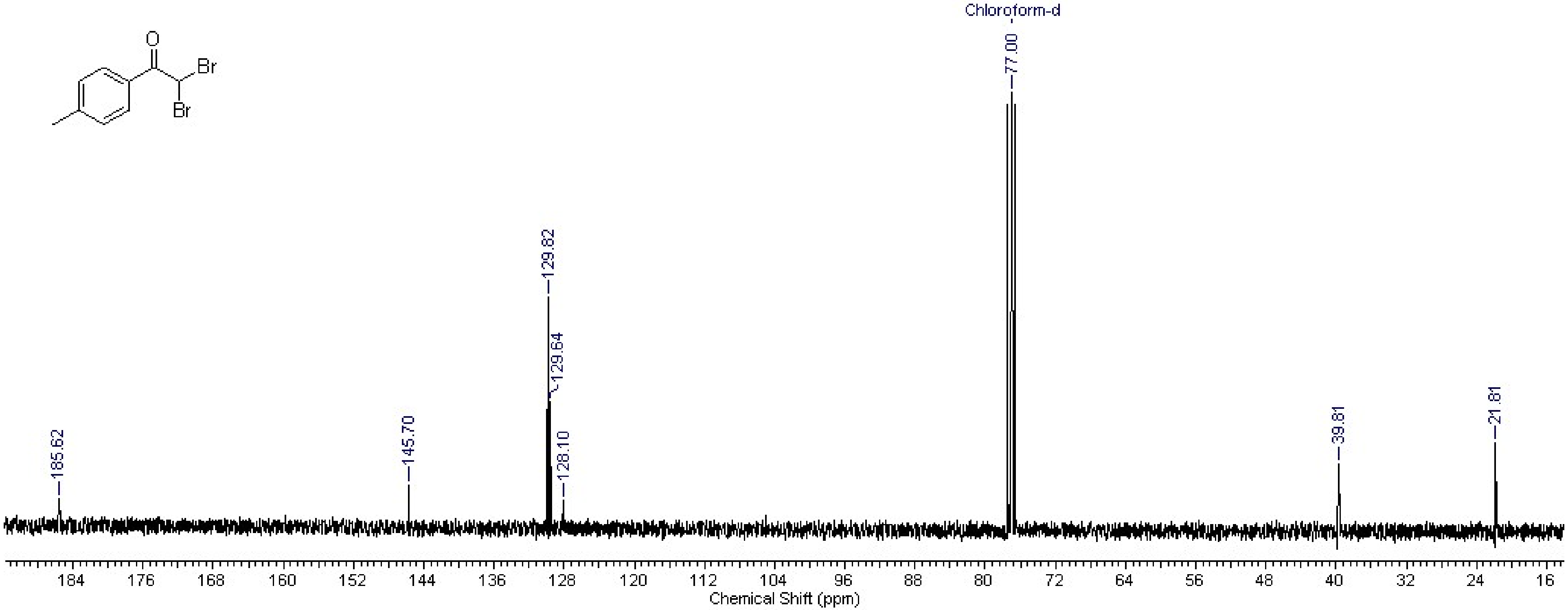

No.	(ppm)	(Hz)	Height
1	21.81	1646.4	0.1888
2	39.81	3004.4	0.1410
3	77.00	5811.6	1.0000
4	128.10	9668.7	0.0566
5	129.64	9784.3	0.2844
6	129.82	9798.6	0.5257
7	145.70	10997.0	0.0927
8	185.62	14010.1	0.0597

No.	Annotation	(ppm)
1	Chloroform-d	77.00

^{1}H NMR of 2,2-dibromo-1-(2-chlorophenyl)ethan-1-one, **6e**

Acquisition Time (sec)	2.7150

Frequency (MHz)	300.13	Nucleus	1H	Number of Transients	1	Original Points Count	16316
Points Count	16384	Pulse Sequence	zg	Solvent	CHLOROFORM-D	Sweep Width (Hz)	6009.62
Temperature (degree C)	27.005						

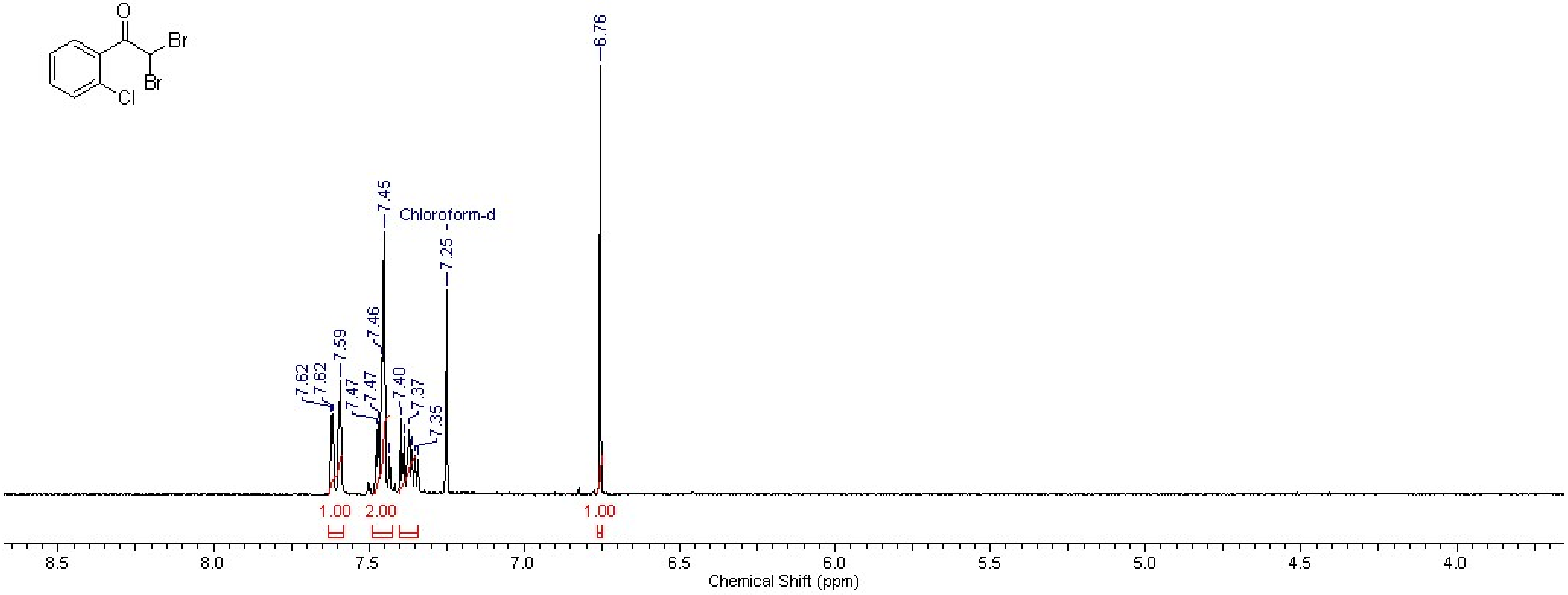

No.	(ppm)	(Hz)	Height
1	6.76	2027.4	1.0000
2	7.25	2176.0	0.4770
3	7.34	2203.8	0.0810
4	7.35	2206.8	0.0891
5	7.36	2209.7	0.1037
6	7.37	2212.3	0.1531
7	7.39	2217.4	0.1320
8	7.40	2220.0	0.1757

No.	(ppm)	(Hz)	Height
9	7.43	2231.0	0.0875
10	7.45	2236.1	0.6107
11	7.46	2239.1	0.3184
12	7.47	2242.0	0.1696
13	7.47	2243.5	0.1524
14	7.59	2279.0	0.2639
15	7.62	2286.0	0.1855
16	7.62	2287.5	0.1832

No.	Annotation	(ppm)
1	Chloroform-d	7.25

^{13}C NMR of 2,2-dibromo-1-(2-chlorophenyl)ethan-1-one, **6e**

Acquisition Time (sec)	0.9050

Frequency (MHz)	75.48	Nucleus	13C	Number of Transients	256	Original Points Count	16316
Points Count	16384	Pulse Sequence	zgpg30	Solvent	CHLOROFORM-D	Sweep Width (Hz)	18028.85
Temperature (degree C)	27.129						

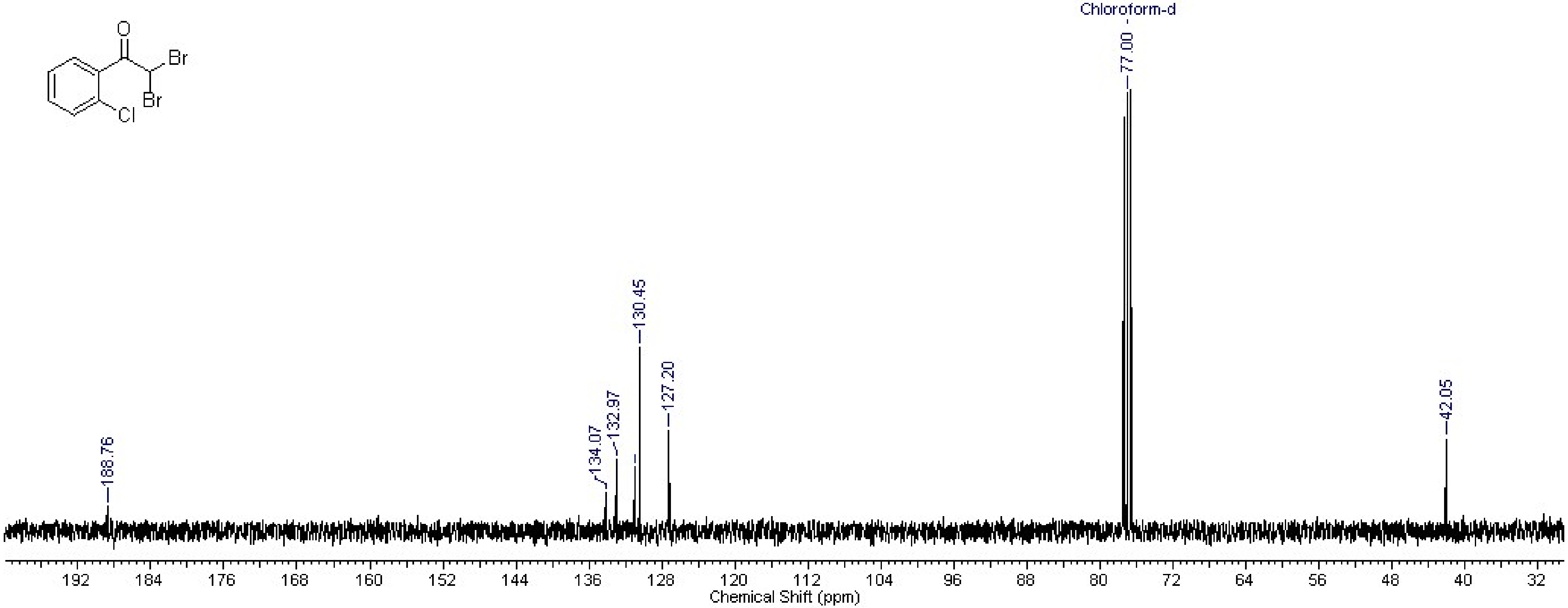

No.	(ppm)	(Hz)	Height
1	42.05	3173.8	0.1968
2	77.00	5811.6	0.9912
3	127.20	9600.5	0.2164
4	130.45	9845.9	0.4065
5	130.90	9880.0	0.1316
6	130.95	9883.3	0.0599
7	132.97	10036.3	0.1503
8	134.07	10118.8	0.0731
9	188.76	14246.7	0.0432

No.	Annotation	(ppm)
1	Chloroform-d	77.00

^{1}H NMR of pentan-3-one, **7a**

Acquisition Time (sec)	1.3518

Frequency (MHz)	300.13	Nucleus	1H	Number of Transients	1	Original Points Count	8124
Points Count	8192	Pulse Sequence	zg	Solvent	CHLOROFORM-D	Sweep Width (Hz)	6009.62
Temperature (degree C)	29.200						

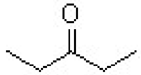

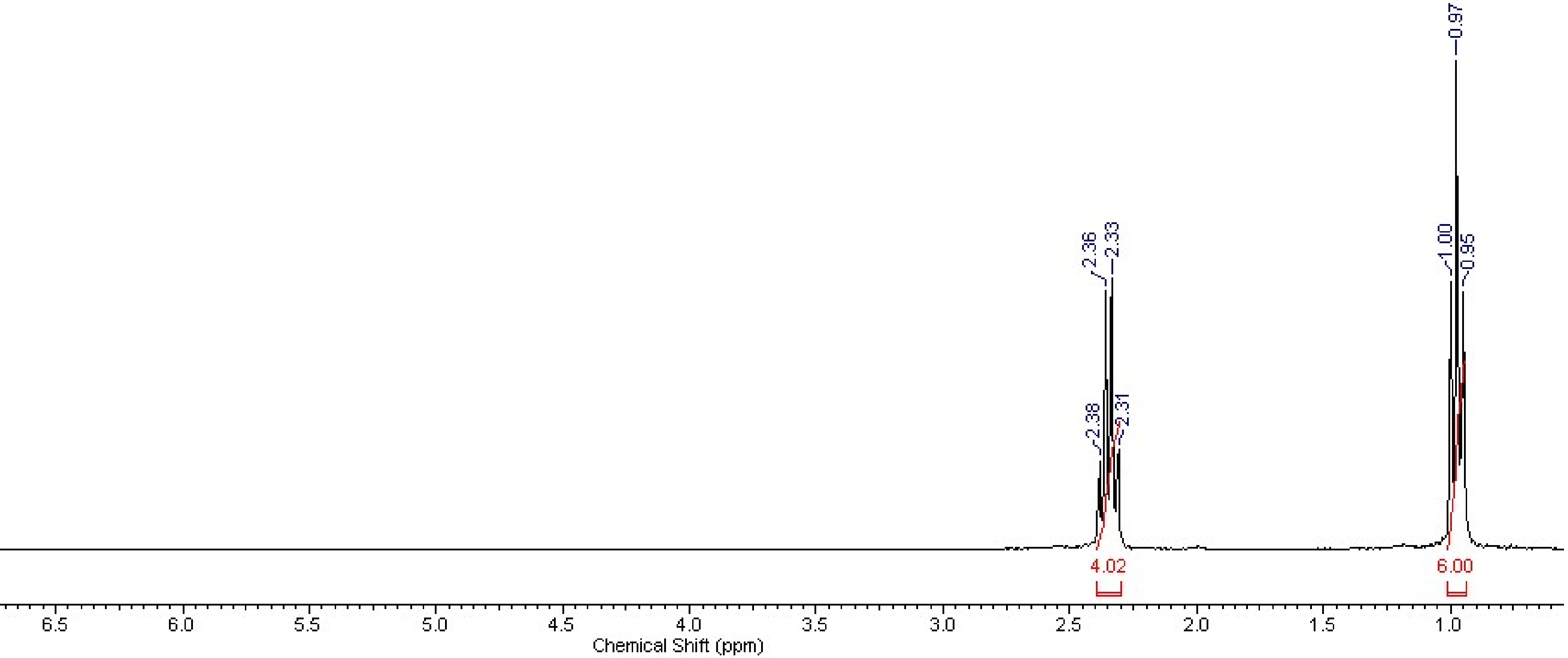

No.	(ppm)	(Hz)	Height
1	0.95	284.5	0.5269
2	0.97	292.6	1.0000
3	1.00	299.9	0.5479
4	2.31	693.2	0.2037
5	2.33	700.5	0.5524
6	2.36	707.9	0.5283
7	2.38	715.2	0.1806

No.	Annotation	(ppm)
1	Chloroform-d	7.25

No.	(ppm)	Value	Absolute Value
1	[0.94 .. 1.01]	6.000	7.42857e+8
2	[2.30 .. 2.40]	4.015	4.97143e+8

HRMS of 8-bromoheptadecan-9-one, **2e**

Display Report

Analysis Info

Analysis Name	D:\Data\Kolotyrkina\2018\Kapustina\0213013.d	Acquisition Date	13.02.2018 12:12:13
Method	tune_low.m	Operator	BDAL@DE
Sample Name	/KAPN 149	Instrument / Ser#	micrOTOF 10248
Comment	C17H33BrO mH 333.1789 calibrant added		

Acquisition Parameter

Source Type	ESI	Ion Polarity	Positive	Set Nebulizer	0.4 Bar
Focus	Not active			Set Dry Heater	180 °C
Scan Begin	50 m/z	Set Capillary	4500 V	Set Dry Gas	4.0 l/min
Scan End	3000 m/z	Set End Plate Offset	-500 V	Set Divert Valve	Waste

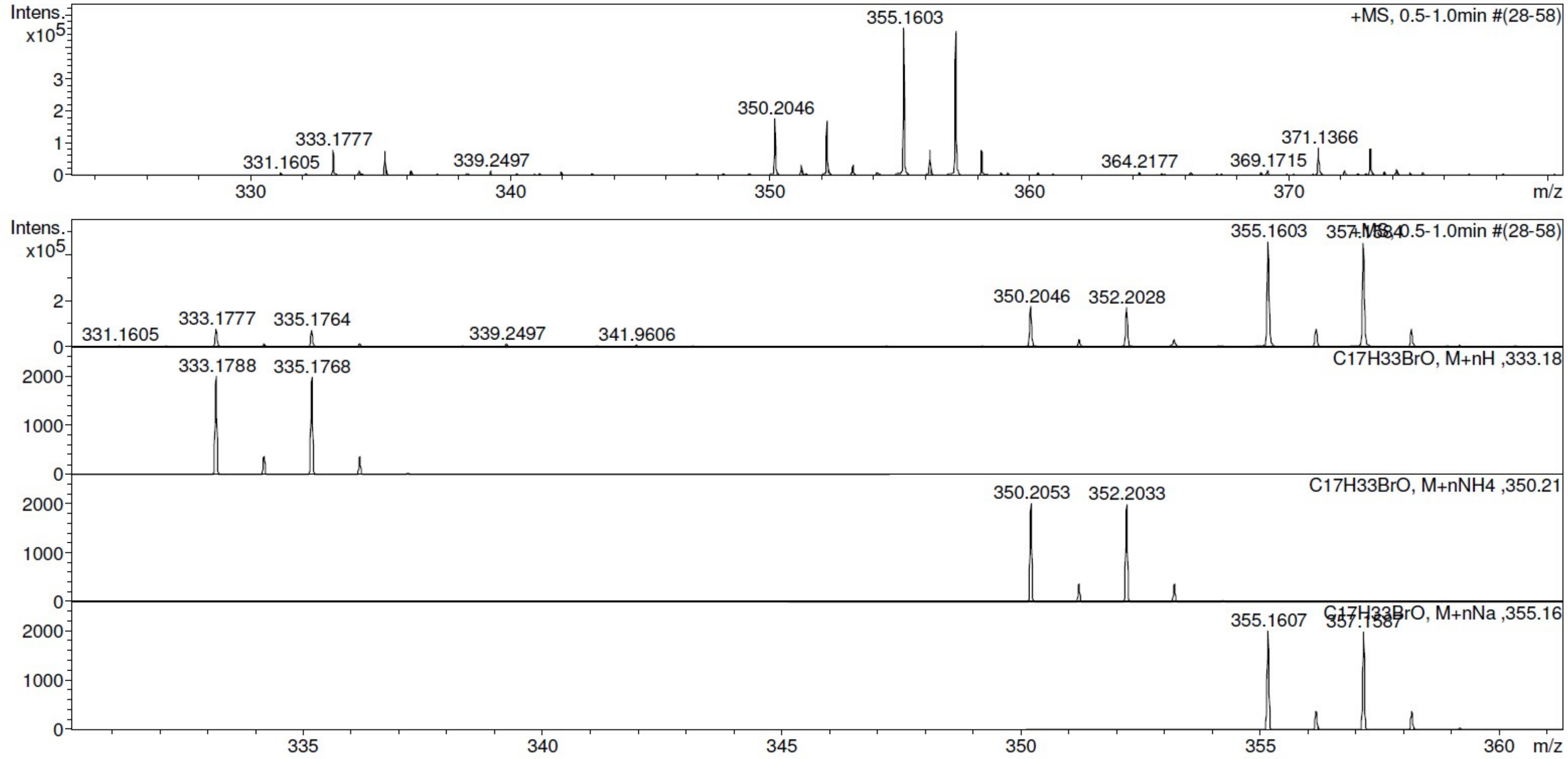

HRMS of смесь 2-bromo-2-methyloctan-3-one **(2g)** и 4-bromo-2-methyloctan-3-one **(2g’)**

Display Report

Analysis Info

Analysis Name	D:\Data\Kolotyrkina\2018\Kapustina\0313046.d	Acquisition Date	13.03.2018 17:52:10
Method	tune_low.m	Operator	BDAL@DE
Sample Name	/KAPN 183	Instrument / Ser#	micrOTOF 10248
Comment	C9H17BrO mH 221.0535 /clb added		

Acquisition Parameter

Source Type	ESI	Ion Polarity	Positive	Set Nebulizer	0.4 Bar
Focus	Not active			Set Dry Heater	180 °C
Scan Begin	50 m/z	Set Capillary	4500 V	Set Dry Gas	4.0 l/min
Scan End	3000 m/z	Set End Plate Offset	-500 V	Set Divert Valve	Waste

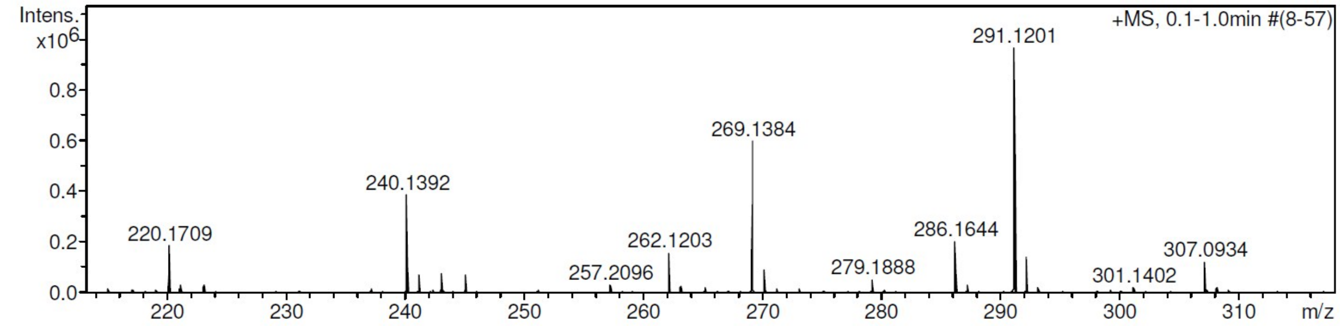

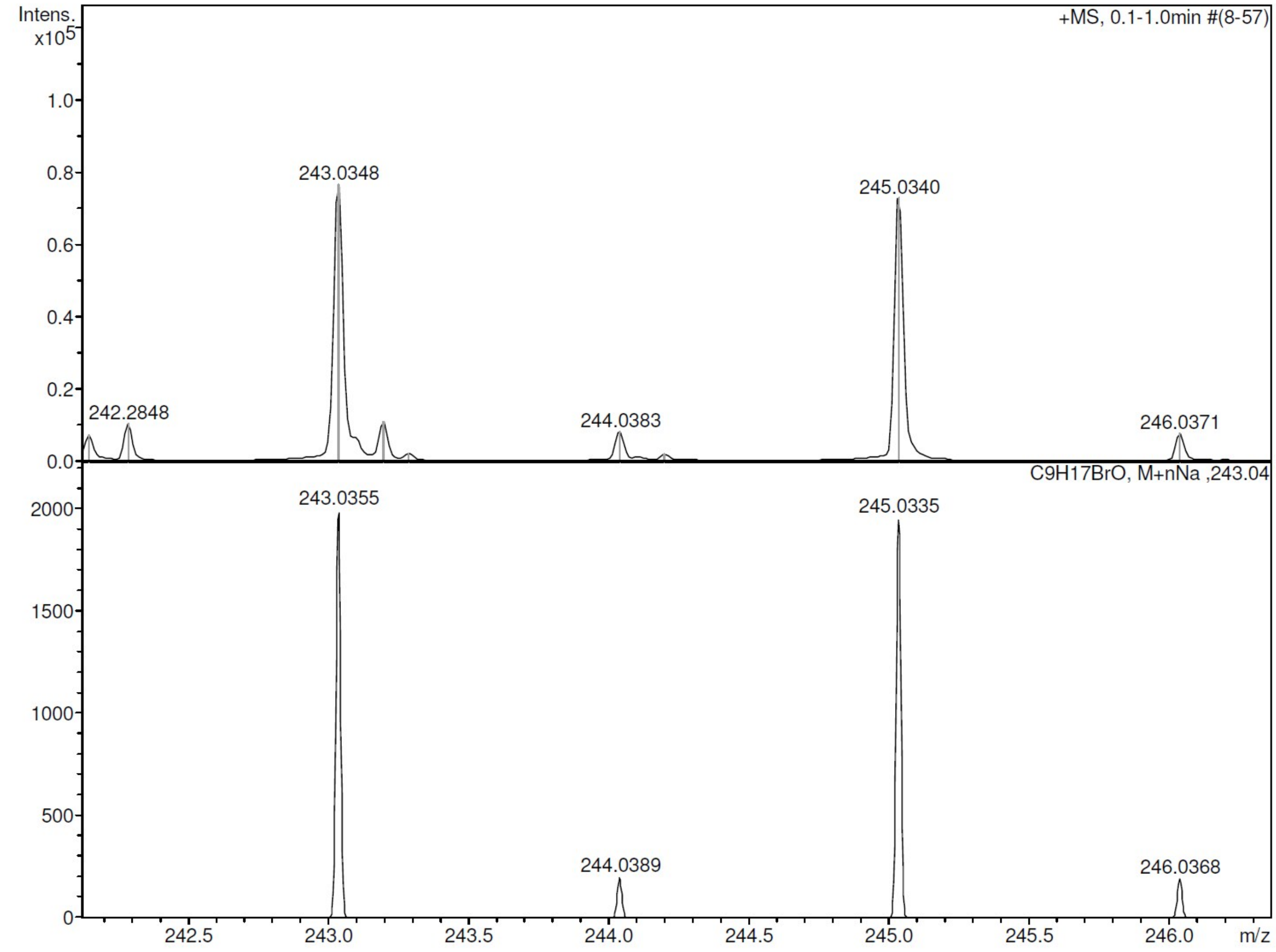

HRMS of 5,7-dibromoundecan-6-one, **3d**

Display Report

Analysis Info

Analysis Name	D:\Data\Kolotyrkina\2018\Kapustina\0228026.d	Acquisition Date	28.02.2018 21:17:31
Method	tune_low.m	Operator	BDAL@DE
Sample Name	/KAPN 141	Instrument / Ser#	micrOTOF 10248
Comment	C11H20Br2O mH 326.9953/ clb dded CH3OH		

Acquisition Parameter

Source Type	ESI	Ion Polarity	Positive	Set Nebulizer	0.4 Bar
Focus	Not active			Set Dry Heater	180 °C
Scan Begin	50 m/z	Set Capillary	4500 V	Set Dry Gas	4.0 l/min
Scan End	3000 m/z	Set End Plate Offset	-500 V	Set Divert Valve	Waste

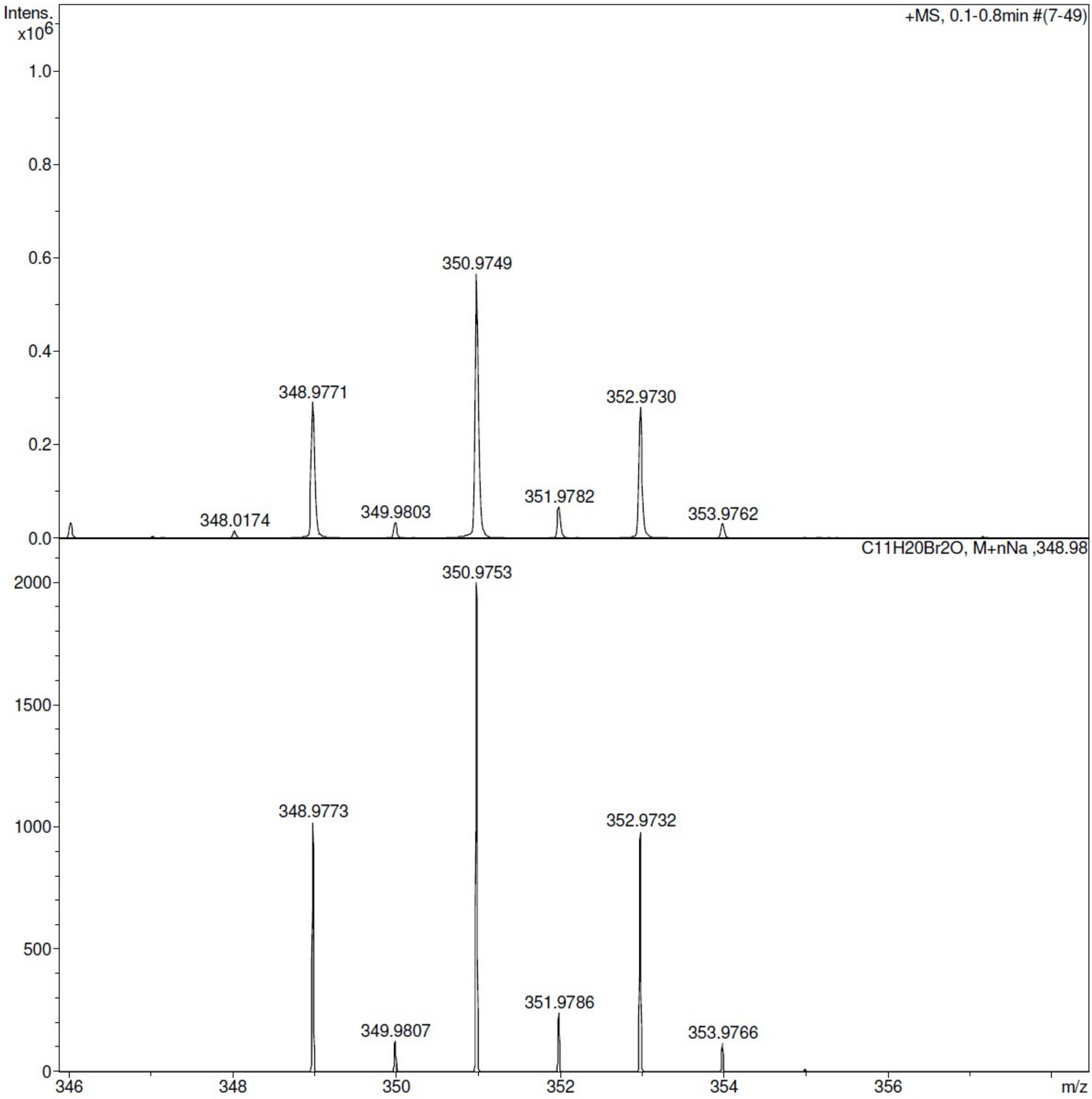

Bruker Compass DataAnalysis 4.0 printed: 28.02.2018 21:20:48 Page 1 of 1

HRMS of 8,10-dibromoheptadecan-9-one, **3e**

Display Report

**Analysis Info**		Acquisition Date	13.02.2018 12:04:00
Analysis Name	D:\Data\Kolotyrkina\2018\Kapustina\0213012.d		
Method	tune_low.m	Operator	BDAL@DE
Sample Name	/KAPN 149	Instrument / Ser#	micrOTOF 10248
Comment	C17H32Br2O mH 411.0892 calibrant added		

Acquisition Parameter

Source Type	ESI	Ion Polarity	Positive	Set Nebulizer	0.4 Bar
Focus	Not active			Set Dry Heater	180 °C
Scan Begin	50 m/z	Set Capillary	4500 V	Set Dry Gas	4.0 l/min
Scan End	3000 m/z	Set End Plate Offset	-500 V	Set Divert Valve	Waste

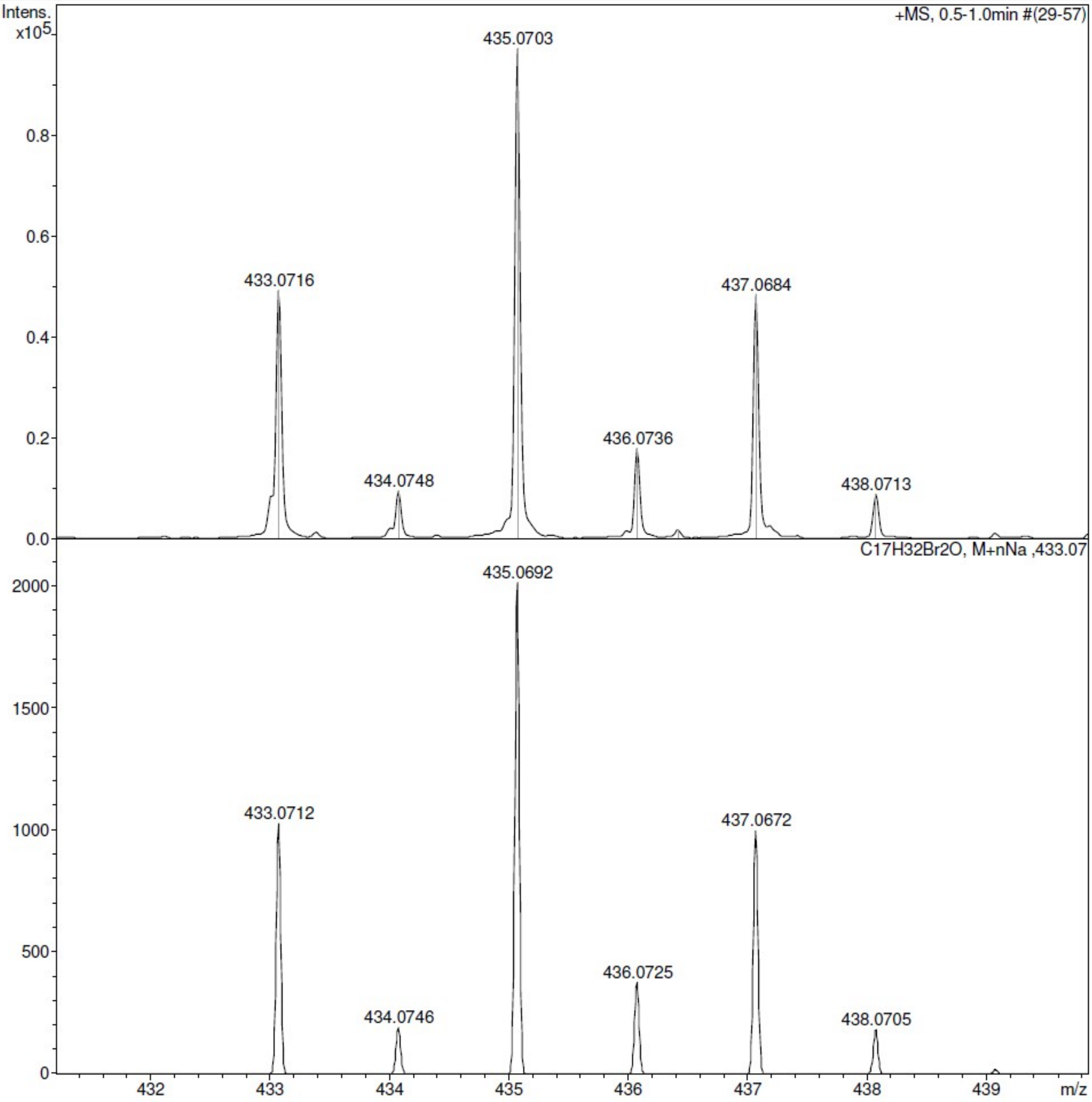

HRMS of 3,5-dibromononan-4-one, **3f**

Display Report

Analysis Info

Analysis Name	D:\Data\Kolotyrkina\2018\Kapustina\0314027.d	Acquisition Date	14.03.2018 18:36:27
Method	tune_low.m	Operator	BDAL@DE
Sample Name	/KAPN 50	Instrument / Ser#	micrOTOF 10248
Comment	C9H16Br2O mH 298.9640/ clb added CH3OH		

Acquisition Parameter

Source Type	ESI	Ion Polarity	Positive	Set Nebulizer	0.4 Bar
Focus	Not active			Set Dry Heater	180 °C
Scan Begin	50 m/z	Set Capillary	4500 V	Set Dry Gas	4.0 l/min
Scan End	3000 m/z	Set End Plate Offset	-500 V	Set Divert Valve	Waste

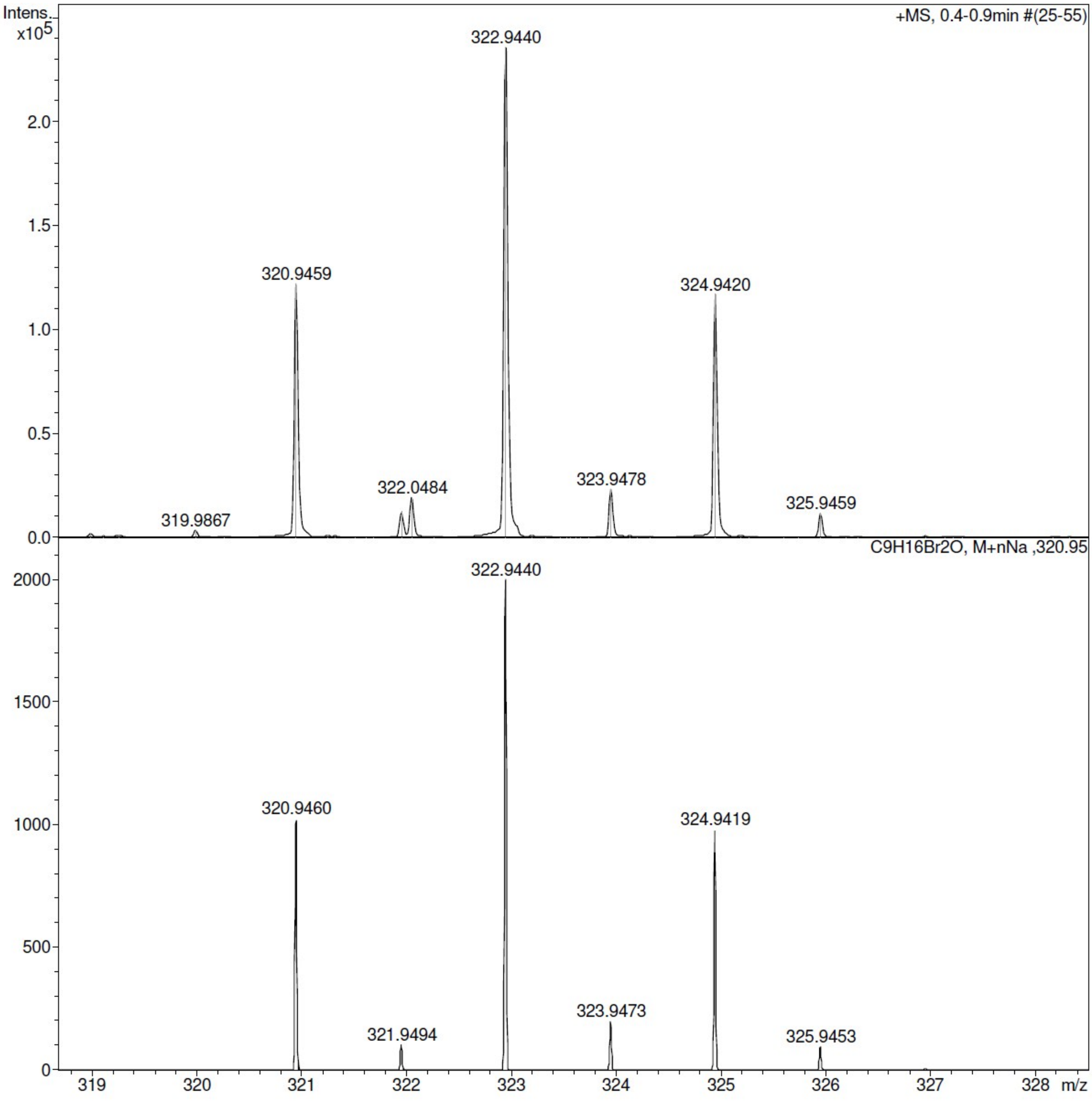

HRMS of 2,4-dibromo-2-methyloctan-3-one, **3g**

Display Report

**Analysis Info**		Acquisition Date	22.03.2018 15:24:46
Analysis Name	D:\Data\Kolotyrkina\2018\Kapustina\0322026.d		
Method	tune_low.m	Operator	BDAL@DE
Sample Name	/KAPN 68_90	Instrument / Ser#	micrOTOF 10248
Comment	C9H16Br2O mH 298.9640/ clb added,		

Acquisition Parameter

Source Type	ESI	Ion Polarity	Positive	Set Nebulizer	0.4 Bar
Focus	Not active			Set Dry Heater	180 °C
Scan Begin	50 m/z	Set Capillary	4500 V	Set Dry Gas	4.0 l/min
Scan End	3000 m/z	Set End Plate Offset	-500 V	Set Divert Valve	Waste

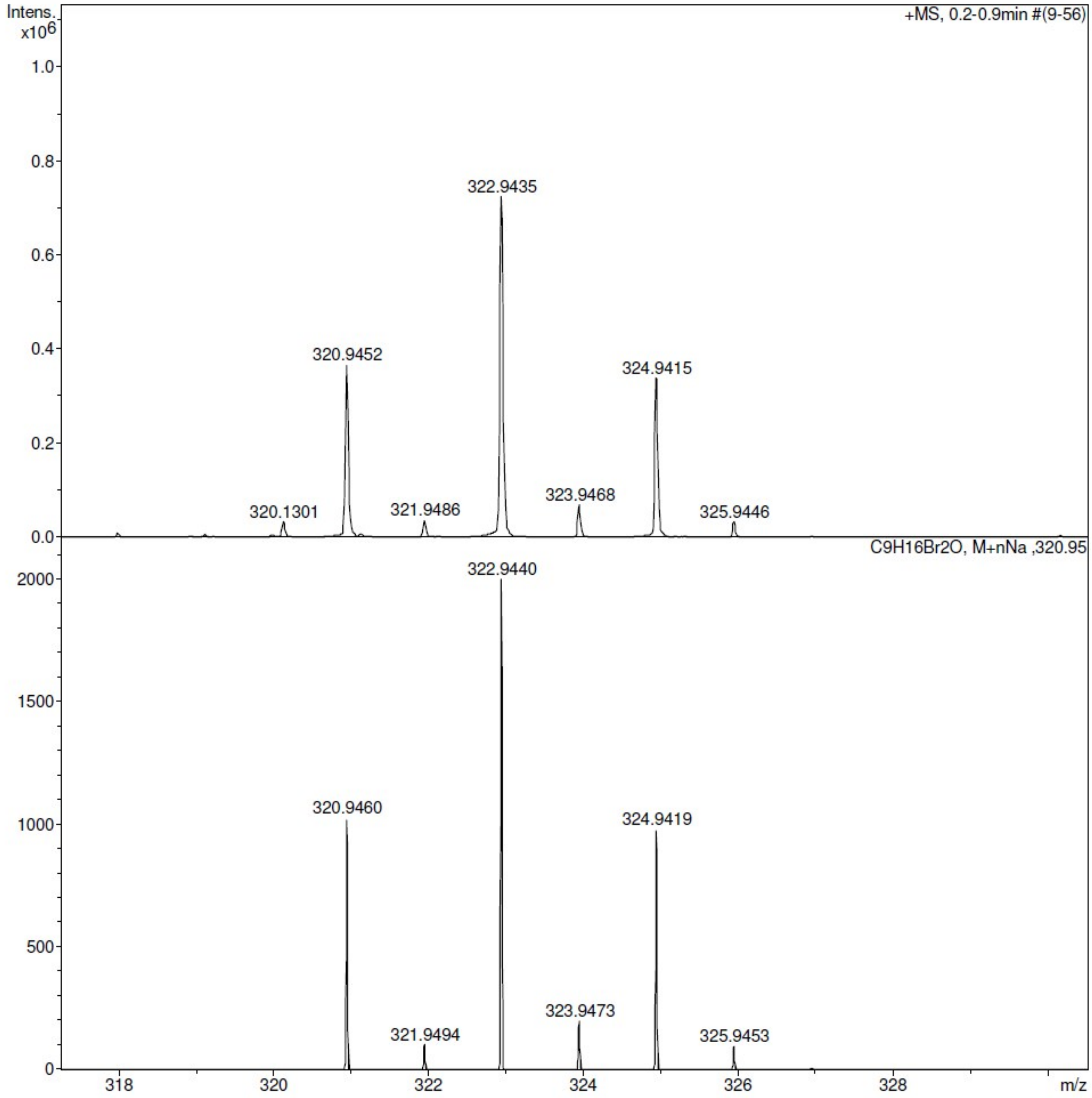

Supplement: RA-008-C8RA04885A-s001 [file RA-008-C8RA04885A-s001.pdf]
